# Computational modeling of cardiac electrophysiology and arrhythmogenesis: toward clinical translation

**DOI:** 10.1152/physrev.00017.2023

**Published:** 2023-12-28

**Authors:** Natalia A. Trayanova, Aurore Lyon, Julie Shade, Jordi Heijman

**Affiliations:** ^1^Department of Biomedical Engineering, Johns Hopkins University, Baltimore, Maryland, United States; ^2^Alliance for Cardiovascular Diagnostic and Treatment Innovation, Johns Hopkins University, Baltimore, Maryland, United States; ^3^Department of Biomedical Engineering, CARIM School for Cardiovascular Diseases, Maastricht University, Maastricht, The Netherlands; ^4^Division of Heart and Lungs, Department of Medical Physiology, University Medical Centre Utrecht, Utrecht, The Netherlands; ^5^Department of Cardiology, CARIM School for Cardiovascular Diseases, Maastricht University, Maastricht, The Netherlands

**Keywords:** arrhythmias, atrial fibrillation, cardiac electrophysiology, computational modeling, sudden cardiac death

## Abstract

The complexity of cardiac electrophysiology, involving dynamic changes in numerous components across multiple spatial (from ion channel to organ) and temporal (from milliseconds to days) scales, makes an intuitive or empirical analysis of cardiac arrhythmogenesis challenging. Multiscale mechanistic computational models of cardiac electrophysiology provide precise control over individual parameters, and their reproducibility enables a thorough assessment of arrhythmia mechanisms. This review provides a comprehensive analysis of models of cardiac electrophysiology and arrhythmias, from the single cell to the organ level, and how they can be leveraged to better understand rhythm disorders in cardiac disease and to improve heart patient care. Key issues related to model development based on experimental data are discussed, and major families of human cardiomyocyte models and their applications are highlighted. An overview of organ-level computational modeling of cardiac electrophysiology and its clinical applications in personalized arrhythmia risk assessment and patient-specific therapy of atrial and ventricular arrhythmias is provided. The advancements presented here highlight how patient-specific computational models of the heart reconstructed from patient data have achieved success in predicting risk of sudden cardiac death and guiding optimal treatments of heart rhythm disorders. Finally, an outlook toward potential future advances, including the combination of mechanistic modeling and machine learning/artificial intelligence, is provided. As the field of cardiology is embarking on a journey toward precision medicine, personalized modeling of the heart is expected to become a key technology to guide pharmaceutical therapy, deployment of devices, and surgical interventions.

## Graphical abstract

**Figure:**
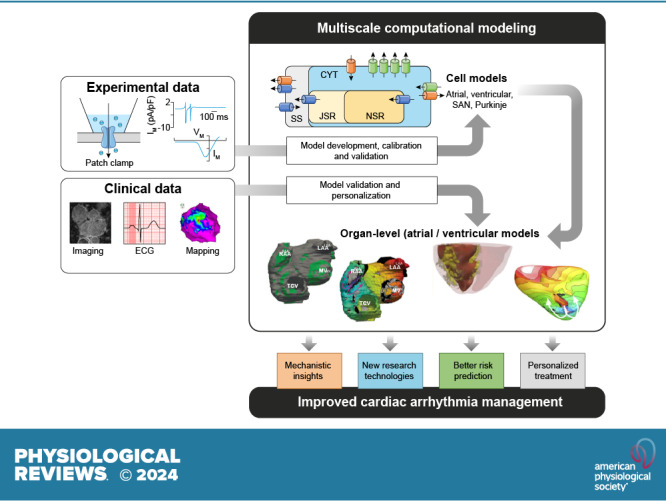

CLINICAL HIGHLIGHTS

- Computational modeling of the heart has become an important research tool in cardiac electrophysiology and arrhythmias, as it provides a link between effects at the subcellular level, including genetic mutations and protein function, and emergent electrophysiological phenomena at the organ level.
- Electrophysiological models of the heart are currently being employed in real-world applications in mechanistic research, safety pharmacology, and personalized therapy.
- Computational modeling of the heart has been used to support new research technologies such as cardiac optogenetics and cardiac regenerative therapies.
- Patient-specific computational models of the heart reconstructed from noninvasive patient clinical imaging and other patient data have demonstrated excellent capabilities in predicting risk of sudden cardiac death in patients with both ischemic and nonischemic cardiomyopathies.
- Personalized atrial and ventricular models have made significant contributions to guiding optimal treatment of heart rhythm disorders.

## 1. INTRODUCTION: CARDIAC ELECTROPHYSIOLOGY AND ARRHYTHMOGENESIS

### 1.1. Cardiac Electrophysiology and Arrhythmogenesis

The heart is a fascinatingly complex organ. Each of the ∼3 billion heartbeats that occur during an average person’s lifetime requires the synchronized contraction of billions of heart muscle cells (cardiomyocytes) to promote the rhythmic pumping of blood that is essential for life. This synchronized activity is orchestrated by an intricate electrical system (1–3).

Each heartbeat is initiated through spontaneous electrical activation of pacemaker cells in the sinoatrial node (SAN) (4). The resulting electrical impulse spreads across the upper chambers of the heart (atria) through a self-regenerating process in which activation of each cardiomyocyte generates an electrical action potential (AP) that contributes to the activation of neighboring cardiomyocytes via electrical cell-to-cell connections, primarily mediated by gap junctions (3). After reaching the atrioventricular (AV) node, the electrical impulse is delayed to provide sufficient time for atrial contraction and for the ventricles to fill with blood. Thereafter, it rapidly conducts along the His–Purkinje system to provide a synchronized and rapid electrical activation of both ventricles, promoting the synchronous contraction underlying the heart’s efficient pump function. The exact electrophysiological properties of each cardiac region are highly dynamic and modulated by numerous neurohumoral and mechanical factors to adjust the heart’s electrical and mechanical properties for varying demands (5–7). Furthermore, these properties depend on the interaction with the structural substrate, including the direction of the muscle fibers in the heart and the presence of structural remodeling in the diseased heart (8, 9).

Heart rhythm disturbances (cardiac arrhythmias) are a major cause of cardiovascular morbidity and mortality (10). For example, atrial fibrillation (AF) is a highly prevalent cardiac arrhythmia, affecting an estimated 55 million individuals worldwide. Although not directly life threatening, AF is associated with increased risk of stroke and heart failure (HF), doubling cardiovascular morbidity and mortality (11) and contributing directly or indirectly to 1–3% of all health care expenses (12). Ventricular tachyarrhythmias (VTs) can be immediately life threatening, particularly when degenerating into ventricular fibrillation (VF), as they prevent normal cardiac contraction and impair the supply of oxygen-rich blood to the brain. VTs/VFs account for more than half of all sudden cardiac deaths (SCDs), which in turn make up ∼20% of all deaths in the industrialized world (13).

Despite significant technological, scientific, and medical advances over the past 50 years, the modern management of cardiac arrhythmias remains suboptimal. The development of novel personalized approaches, taking into account patient-specific (risk) profiles and arrhythmia mechanisms, will likely be essential to overcome current challenges in cardiac arrhythmia management (14, 15). The complexity of cardiac electrophysiology and arrhythmogenesis, involving dynamic changes in numerous components across multiple spatial (from ion channel to organ) and temporal (from milliseconds to days) scales, makes an intuitive or empirical analysis of the system challenging. On the other hand, its quantitative nature, controlled by physical laws and biochemical concepts that can be reliably and, to some extent, noninvasively quantified in patients, is highly suitable for mechanistic and data-driven computational modeling approaches. Computational models of cardiac electrophysiology provide precise control over individual parameters, and their reproducibility enables a comprehensive assessment of arrhythmia mechanisms. As such, there has been a growing interest in the use of computational modeling for both mechanistic studies and clinical management of cardiac arrhythmias over the past few decades (reviewed in Refs. 16–23). In this review, we provide a comprehensive overview of mechanistic computational models of cardiac electrophysiology and arrhythmias, from the single cell to the organ level, and how they can be leveraged to better understand rhythm disorders in cardiac disease and to improve heart patient care. We start by providing the reader with a brief introduction to arrhythmia mechanisms (sect. 1.2) and a historic overview of computational modeling (sect. 1.3). Next, the review is organized into two major sections. The first (sect. 2) reviews human cardiomyocyte models for all different cell types; we detail key methodological issues and major families of cell models. We discuss their application in elucidating mechanisms of pacemaking and cellular electrical dysfunction determinants, in predicting the effects of genetic mutations in cardiac ion channels, and in evaluating the effects of antiarrhythmic drugs and their role in cardiac safety testing. The second major section of this review (sect. 3) addresses the advances in organ-level multiscale computational modeling of cardiac electrophysiology and arrhythmias. Multiscale organ-level models incorporate mechanisms at the cell and tissue levels as well as the geometrical and structural remodeling factors that might play a significant role in arrhythmogenesis. As tissue-level mechanisms have been either confirmed at the organ level or not proven to be essential at the organ level, where disease remodeling also plays a major role, we found it most advantageous to review the organ-level achievements of computational modeling in electrophysiology and arrhythmias (aside from the inclusion of tissue-level models in the brief historical overview in sect. 1.2). This choice also enabled us to focus sect. 3, to a large degree, on clinical applications in personalized arrhythmia risk assessment and patient-specific therapy. Finally, we conclude the review with future perspectives (sect. 4).

### 1.2. Conceptual Overview of Cardiac Arrhythmogenesis

The current state of the art in our understanding of the mechanisms underlying cardiac arrhythmogenesis has been summarized in detail in dedicated reviews (1–3, 24). In brief, most cardiac arrhythmias result from abnormal impulse formation, abnormal impulse conduction, or a combination of the two. In the healthy heart, SAN cells are responsible for pacemaking through spontaneous AP generation, a process termed automaticity. Impaired SAN automaticity can lead to excessively slow or irregular impulse formation and sinus node dysfunction. Abnormal impulse formation (also known as ectopy) can also occur in the atria or ventricles, either because of abnormal automaticity of cardiomyocytes that normally do not generate spontaneous APs or because of triggered activity. Triggered activity refers to the triggering of additional APs independent of the surrounding tissue resulting from properties of the preceding AP. Triggered activity can arise from secondary depolarizations of the membrane potential occurring during the preceding AP (so-called early afterdepolarizations, EADs) or after the preceding AP (so-called delayed afterdepolarizations, DADs). If these depolarizations are sufficiently strong to overcome the electrotonic load of the surrounding myocardium, a new AP can be triggered. EADs typically occur in the setting of excessive prolongation of repolarization, whereas DADs are associated with abnormalities in intracellular Ca^2+^ handling (2, 25).

Abnormal automaticity and triggered activity can by themselves give rise to tachyarrhythmias when occurring repetitively at high rate. However, tachyarrhythmias are more commonly maintained by so-called reentrant activity, in which an electrical impulse continuously finds excitable tissue by “chasing its own tail.” Reentry occurs when abnormal automaticity or triggered activity elicits ectopic activity in a vulnerable substrate. Depending on the timing of the triggered activity and the properties of the substrate, the electrical impulse may block in one direction but conduct in another direction, allowing the impulse to travel across the myocardium around the site of block until this region has become excitable again, causing reactivation of the original site of triggered activity and starting the next cycle of the reentrant activity (26). Reentry is therefore promoted by slow conduction velocities (e.g., due to fibrosis or impaired electrical cell-to-cell coupling) and short effective refractory periods (e.g., resulting from shortening of AP duration), as these promote the availability of reexcitable tissue. In addition, heterogeneities in excitability (e.g., due to the presence of scar) or refractoriness provide a substrate for the occurrence of conduction block and pathways for reentrant activity. Reentry can occur around a fixed anatomical obstacle (e.g., an area of dense scar tissue) but can also occur in the absence of structural abnormalities. In the latter case, reentry typically takes the form of a spiral wave circulating around a stationary or meandering core (27, 28).

The complex interaction between trigger and vulnerable substrate, each dynamically regulated by multiple molecular mechanisms that often have both pro- and antiarrhythmic effects, makes an intuitive assessment of arrhythmogenic risk or optimal antiarrhythmic therapy challenging. Although the abilities to phenotype the arrhythmogenic substrate in humans are expanding, an in-depth molecule-to-organ characterization under specific conditions relevant for arrhythmogenesis (e.g., taking into account dynamic modulators such as the autonomic nervous system, inflammation, or electrolyte disturbances) is currently not possible. Computer models of human cardiac electrophysiology provide a platform for integrating data, understanding complex arrhythmia mechanisms, assessing arrhythmogenic risk, and optimizing antiarrhythmic therapies, as detailed in the remainder of this review.

### 1.3. Computational Modeling of Cardiac Electrophysiology: a Brief History

The seminal experimental work on electrical activity in the squid giant axon by Hodgkin and Huxley in 1952 (29) inspired the first mechanistic electrophysiological model. This model demonstrated that the experimentally observed AP could be quantitatively reproduced by an electrical circuit with three ionic currents. Subsequently, numerous mathematical models of both neural and cardiac electrophysiology have been developed, each designed to address specific research questions. In 1962, Denis Noble (30) for the first time utilized the Hodgkin–Huxley equations to study mechanisms underlying the long plateau of the cardiac AP. A similar approach was employed by Beeler and Reuter (31) to develop the first ventricular cardiomyocyte model in 1977. Subsequent experimental advances, notably the development of the patch-clamp methodology by Sakmann and Neher (32) and the discovery of pharmacological tools to isolate individual ion currents, provided a wealth of information on biophysical properties and regulation of the ionic determinants of the cardiac AP, which enabled the development of detailed mechanistic models. For example, the 1985 model by DiFrancesco and Noble (33) was the first to also consider changes in intracellular and extracellular ion concentrations during the AP, including Ca^2+^ uptake and release from the intracellular stores of the sarcoplasmic reticulum (SR), based on newly acquired experimental data. Subsequent studies in the 1990s and 2000s provided models for specific cardiac regions (e.g., SAN, atrial cardiomyocytes, ventricular cardiomyocytes) and species (e.g., mouse, guinea pig, rabbit, dog), with a number of key models such as the 1994 model of the guinea pig ventricular cardiomyocyte by Luo and Rudy (34) forming the basis for hundreds of subsequent modeling studies.

Additional advances in molecular biology, including (superresolution) confocal microscopy and fluorescent reporters for Ca^2+^ and other intracellular signaling molecules in the 2000s and 2010s, together with growing computational power, provided the basis for more complex cardiomyocyte models. For example, spatial representations of intracellular Ca^2+^ handling were incorporated in so-called local control models to simulate the physiological characteristics of local Ca^2+^ release from the sarcoplasmic reticulum (SR) as well as spontaneous Ca^2+^ release events (35, 36). Subsequently, these models were used to explore the effects of subcellular differences between atrial and ventricular cardiomyocytes, with the former having a less well-developed t-tubular structure, creating larger heterogeneities in intracellular ionic concentrations (37). Furthermore, signaling pathways underlying posttranslational ion channel regulation, e.g., by the sympathetic nervous system via β-adrenergic receptors and Ca^2+^/calmodulin-dependent protein kinase II (CaMKII) signaling, have been incorporated in several models to study the dynamic regulation of cardiac electrophysiology that contributes to arrhythmogenesis (38–43).

Importantly, since the first models of the human atrial cardiomyocyte by Nygren et al. (44) and Courtemanche et al. (45) in 1998, several models of human cardiac cellular electrophysiology have been developed based on experimental data from explanted hearts or samples from patients undergoing open heart surgery (summarized in sect. 2.3). These models have provided important insights into the cellular determinants of cardiac arrhythmias under a wide range of conditions and are increasingly being used for clinical and commercial applications (sect. 2.4). Nevertheless, cardiac arrhythmias are inherently tissue-level phenomena, and tissue- or organ-level models are needed to study the incidence and behavior of cardiac arrhythmias.

Already in 1964, Moe et al. (46) developed a model of impulse conduction in virtual nonuniform two-dimensional (2-D) tissue. This model could simulate self-sustained turbulent activity resembling AF and provided insights into the determinants of AF maintenance, which subsequently gave rise to the Cox maze procedure, a surgical procedure that electrically separated the atria into compartments that were too small to sustain reentrant activity (47). Advances in computational power enabled the transition from such network-based (automata) models to the simulation of large numbers of electrotonically coupled cardiomyocyte models. Initial work in the late 1980s and early 1990s employed one-dimensional strands or two-dimensional virtual tissue to assess the electrophysiological effects of electrotonic coupling and the determinants of reentrant electrical activity (48, 49). These models have subsequently been used to investigate the effects of ionic remodeling and pharmacological interventions on reentry stability (50, 51).

Since the early 2000s, morphologically realistic organ-level models, incorporating the major anatomic structures, have also been developed (52). Advances in noninvasive clinical imaging, primarily computed tomography (CT) and cardiac magnetic resonance (CMR) imaging, and computational resources have subsequently enabled the development of patient-specific models (53, 54). These models have been employed for risk prediction and treatment optimization of atrial and ventricular arrhythmias (discussed in sect. 3) and have given rise to the first prospective randomized clinical trials comparing simulation-guided and usual care (ClinicalTrials.gov ID NCT04101539; Ref. 55).

**FIGURE 1.**
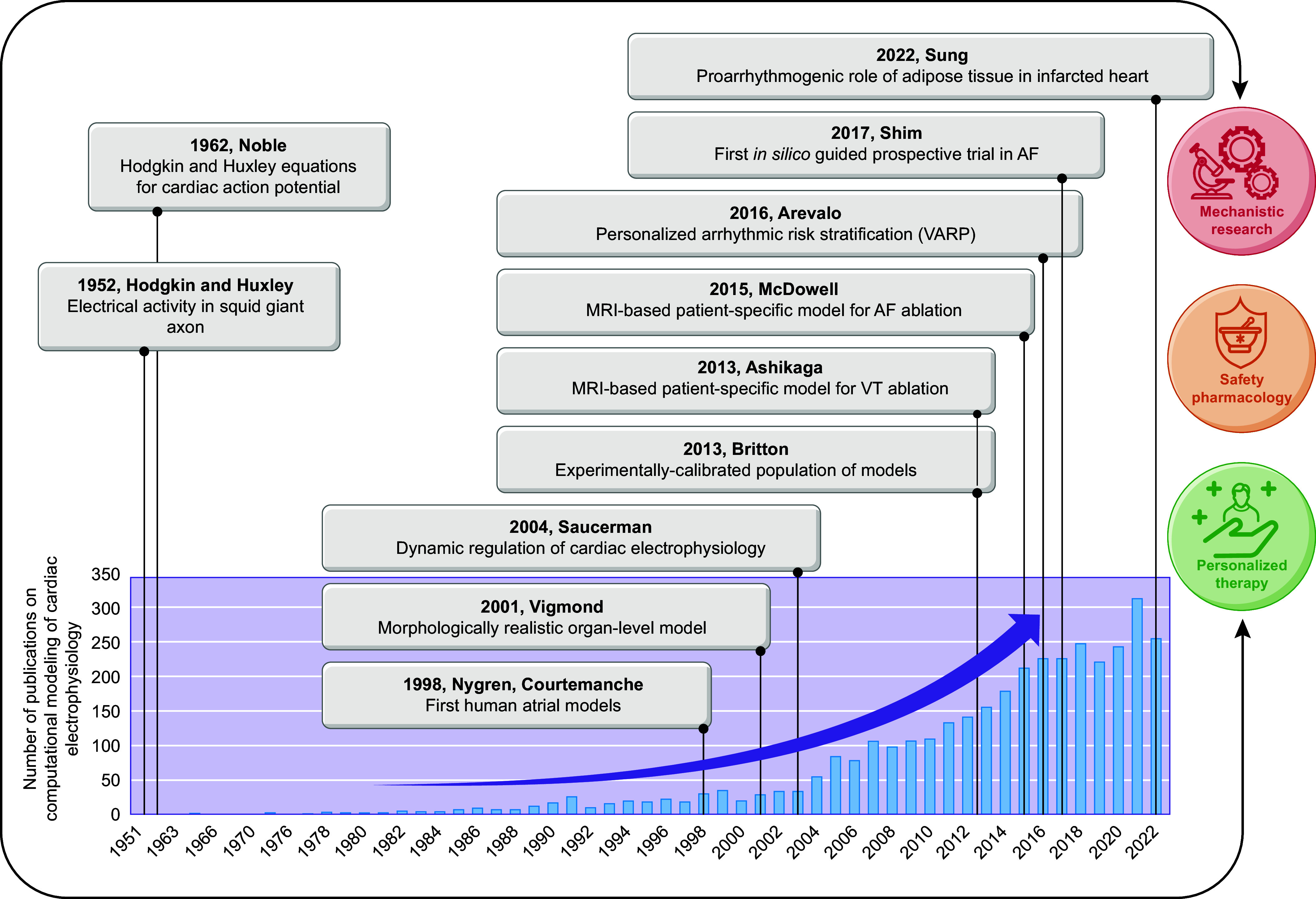
The growing importance of computational modeling of cardiac electrophysiology. The bar chart shows the number of publications on computational modeling of cardiac electrophysiology since the 1950s indexed in PubMed. Key milestones discussed in the historical overview have been highlighted in boxes. Together, these studies have formed the foundation for recent and ongoing work with real-world implications for mechanistic research, safety pharmacology and personalized therapy. AF, atrial fibrillation; VARP, virtual heart arrhythmia predictor; VT, ventricular tachycardia.

Taken together, in the past 60 years tremendous advances have been made in the computational modeling of cardiac electrophysiology. It has become an established field with methodological standards and a growing number of applications (FIGURE 1). In this review, we showcase achievements made by cellular and organ-level modeling of cardiac electrophysiology and arrhythmias. For each scale, key methodological considerations are provided and important models are discussed. Significant emphasis is placed on the various applications of cellular and organ-level models, ranging from improved understanding of the fundamental mechanisms underlying cardiac arrhythmias to applications in the pharmaceutical industry and clinical arrhythmia management. Of note, there have also been significant advances in simulating cardiac mechanics and hemodynamics, which are closely linked to cardiac electrophysiology and arrhythmogenesis but are beyond the scope of the present review. Instead, the interested reader is referred to dedicated reviews on cardiac electromechanics and mechanoelectrical feedback (7, 56).

## 2. COMPUTATIONAL MODELING OF CARDIAC CELLULAR ELECTROPHYSIOLOGY

### 2.1. A Brief Summary of Cardiac Cellular Electrophysiology

#### 2.1.1. The cardiac action potential and excitation-contraction coupling.

**FIGURE 2.**
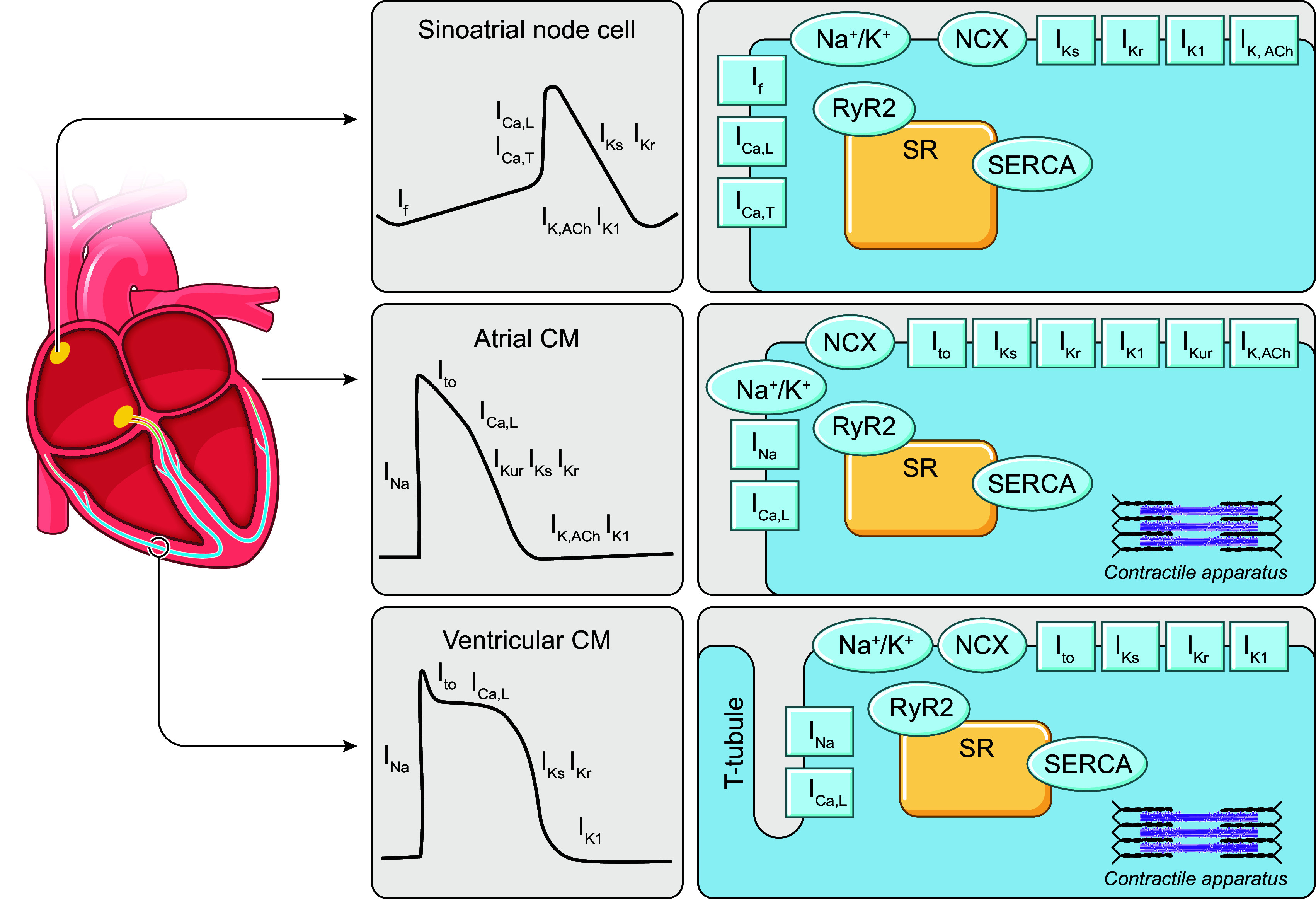
Schematic overview of cardiac cellular electrophysiology in different regions of the heart, including action potentials and underlying ion currents (gray panels), as well as schematic representation of subcellular structures in sinoatrial node cells (*top*), atrial cardiomyocytes (CMs; *middle*), and ventricular CMs (*bottom*). *I*_Ca,L_, L-type Ca^2+^ current; *I*_Ca,T_, T-type Ca^2+^ current; *I*_f_, hyperpolarization-activated cyclic nucleotide-gated “funny” current; *I*_K1_, basal inward-rectifier K^+^ current; *I*_K,ACh_, acetylcholine-activated inward-rectifier K^+^ current; *I*_Kr_, rapid delayed-rectifier K^+^ current; *I*_Ks_, slow delayed-rectifier K^+^ current; *I*_Kur_, ultra-rapid delayed-rectifier K^+^ current; *I*_Na_, Na^+^ current; *I*_to_, transient outward K^+^ current; NCX, Na^+^/Ca^2+^-exchanger; RyR2, ryanodine receptor channel type 2; SERCA, sarco(endo)plasmic reticulum Ca^2+^-ATPase; SR, sarcoplasmic reticulum.

The cardiac AP represents the change in membrane potential (i.e., the charge difference between the inside and outside of the cell) over time during a heartbeat. It results from the dynamic gating of cardiac ion channels, transmembrane proteins that, in their open state, allow specific ions to enter or exit the cell along the electrochemical gradient (FIGURE 2). Moreover, as most cardiac ion channels are voltage dependent because of charged amino acids in their transmembrane domains, this creates a feedback system shaping cardiac electrophysiology on a beat-to-beat basis. The mechanisms underlying cardiac cellular and organ-level electrophysiology, as well as their molecular basis, have recently been highlighted in other reviews in *Physiological Reviews* (2, 7, 57, 58). Key aspects are summarized below.

At rest, the working myocardium has a stable membrane potential below −70 mV. The cardiac AP is initiated by activation of voltage-gated Na^+^ channels in response to a slight depolarization by an external stimulus or electrotonic interaction with neighboring cardiomyocytes. The resulting Na^+^ current (*I*_Na_) promotes a rapid depolarization that subsequently activates other voltage-dependent ion channels. Among these, activation of L-type Ca^2+^ channels (LTCCs) produces a depolarizing current (*I*_Ca,L_) that triggers a much larger release of Ca^2+^ from the intracellular stores of the SR, a process known as Ca^2+^-induced Ca^2+^ release. The resulting Ca^2+^ transient is responsible for the initiation of cardiomyocyte contraction via Ca^2+^-dependent activation of the myofilaments, thus underlying the excitation-contraction coupling process that is fundamental for the pump function of the heart. In parallel, several types of repolarizing K^+^ channels with distinct kinetics are activated, which control repolarization of the membrane potential and thereby modulate refractoriness of the cardiomyocyte (i.e., the interval during which no new AP can be generated). Subsequently, ionic homeostasis is restored by reuptake of Ca^2+^ into the SR via the SR Ca^2+^-ATPase (SERCA) and by Ca^2+^ extrusion from the cell via the Na^+^/Ca^2+^ exchanger (NCX), simultaneously also promoting mechanical relaxation. Finally, Na^+^ and K^+^ gradients are restored by the Na^+^-K^+^-ATPase. Mathematical models incorporating these ion transport mechanisms, such as the prototypical Luo–Rudy model (34, 59) that serves as the foundation for many cardiac AP models (60), can capture a wide range of experimentally observed cellular electrophysiological properties, suggesting that these channels and transporters account for most ion transport under physiological conditions. However, accumulating evidence suggests important roles for other ion channels, including transient receptor potential (TRP), two-pore domain K^+^ and Cl^−^ channels that have traditionally been considered “background” currents in the genesis of cardiac arrhythmias under disease conditions (61, 62). Detailed characterization of these channels under defined pathophysiological conditions and their subsequent incorporation into models of cardiac cellular electrophysiology are expected to increase our understanding of their role in cardiac arrhythmogenesis.

Cells from the SAN, atrioventricular node, and, to a lesser extent, His–Purkinje system express a different complement of ion currents that controls their spontaneous rhythmic electrical activation due to a diastolic depolarization of the resting membrane potential (automaticity; FIGURE 2, *top*). This depolarization is, in part, due to a coupled system of hyperpolarization-activated cyclic nucleotide-gated channels underlying the “funny current” (*I*_f_; often referred to as the membrane clock) and spontaneous Ca^2+^ release promoting depolarizing NCX current (the Ca^2+^ clock) (63). This system has been extensively studied with computational modeling, as detailed in sect. 2.4.1. In addition, *I*_Na_ has a less prominent role in triggering APs in SAN and atrioventricular nodal cells, in part because of the depolarized resting membrane potential. Instead, the AP upstroke is largely carried by *I*_Ca,L_ in these cells.

#### 2.1.2. Species differences.

Preclinical animal experiments are commonly used to provide insights into disease mechanisms, but despite similarities in depolarizing currents between species, electrophysiological differences in repolarization limit the translation of these experimental findings to humans. For example, small rodents are often used to understand molecular mechanisms of arrhythmias (64), but cardiac electrophysiology differs significantly between rodents and humans. For example, resting heart rate is 10-fold higher in rodents, and significant differences in the expression of cardiac K^+^ channels contribute to major differences in AP morphologies (e.g., shorter AP duration and triangular AP shape in the mouse ventricle) (64).

Rabbits and larger mammal models have electrophysiological properties closer to human. They have been used to better understand inherited and acquired arrhythmic disorders (64), but some interspecies differences remain, especially in K^+^ currents like the transient outward K^+^ current (*I*_to_). Dogs are the animal model with the electrophysiology closest to humans and can help investigate aspects of arrhythmogenesis, but quantitative differences in ion channel responses [e.g., to rapid delayed-rectifier K^+^ current (*I*_Kr_) block] remain (65). An alternative to animal models is the use of human induced pluripotent stem cell-derived cardiomyocytes (hIPSC-CMs), which carry the genetic profile of a human donor and therefore help address the interspecies differences mentioned above. However, the variability in cultured cell lines and the frequently observed electrical, structural, and metabolic immaturity of these cells pose a challenge for the translation to adult human cardiomyocytes (66).

Several studies have provided information on electrophysiological properties and ion currents in diseased (explanted) human hearts as well as healthy donor hearts (2, 67–71). In addition, atrial samples can be obtained from patients undergoing open heart surgery, enabling characterization of electrophysiological properties in adult human cardiomyocytes for various disease conditions (15, 72). These human data are essential for the calibration and validation of in silico human cardiomyocyte models (discussed in sect. 2.2.3) but are relatively rare and only available to a limited number of laboratories or are limited to patients with an indication for cardiac surgery in whom only certain areas of the heart can be sampled (typically the atrial appendages). As such, numerous components relevant for simulating cardiac cellular electrophysiology have only been characterized in animal models. Because of the interspecies differences described above, the translation of these mechanisms to humans remains challenging. Nevertheless, recent modeling studies have made great efforts in gathering experimental human data under control and disease conditions (67, 73). Others have proposed translator approaches (74) predicting human response from animal experimental data. Thanks to their ability to integrate data from different species, computational models therefore emerge as powerful tools to translate findings from animal to human.

### 2.2. Key Concepts in Model Development

#### 2.2.1. Types of cellular cardiac electrophysiology models.

The comprehensive characterization of electrophysiological mechanisms is hindered by limitations in the ability to selectively modulate individual components of the system, e.g., because of imperfect selectivity of pharmacological agents or compensatory effects in response to genetic overexpression or knockdown of proteins in animal models. Furthermore, there are limited options to directly measure multiple outcome variables. For example, patch-clamp experiments can only report on total membrane current (in voltage-clamp configuration) or membrane potential (in current-clamp mode). Similarly, intracellular processes can only be measured indirectly, e.g., relying on fluorescent indicators with variable affinity, limited spatial targeting, and partially overlapping spectra (64). By contrast, computational models offer perfect control over model parameters and the ability to analyze multiple outcome variables over time, making them highly suitable for elucidating mechanisms of normal cardiac electrophysiology and arrhythmogenesis.

Cellular models are developed to reproduce the experimentally observed behavior of real cardiomyocytes. To achieve this goal, model calibration (parameter estimation) and validation are essential components of model development that have received significant attention over the last 20 years (discussed in detail in sect. 2.2.3). Patch-clamp recordings of individual ion currents together with whole cell recordings of electrophysiological properties (APs, Ca^2+^ transients) represent the major sources of experimental data for model calibration. The primary goal of most cardiomyocyte models is simulating a cardiac AP. This is done through a system of nonlinear ordinary differential equations that simulates the change in quantities of interest (“state variables”) over time.

**FIGURE 3.**
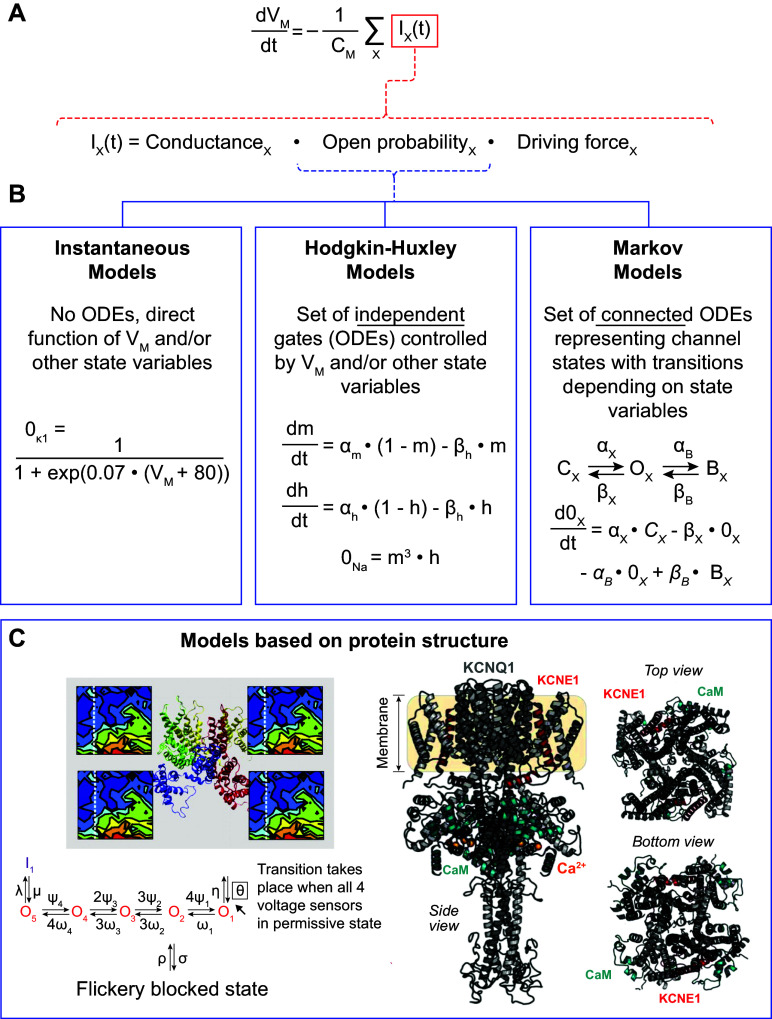
Cardiac cellular electrophysiology modeling approaches. *A*: general equation representing the change in membrane potential (*V*_M_) used to simulate the cardiac action potential as the product of the inverse of membrane capacitance (*C*_M_) and the sum of all ion currents (*I_X_*). The second equation shows the 3 major components determining the magnitude of an ion current *I_X_*. *B*: most commonly used approaches to model the open probability of a cardiac ion channel. Instantaneous models are direct functions of *V*_M_, whereas Hodgkin–Huxley and Markov models are controlled by independent or connected ordinary differential equations (ODEs), respectively. 0_K1_, 0_Na_ and 0_X_ represent the open probability of the inward-rectifier K^+^ channel, Na^+^ channel, and a hypothetical ion channel, respectively, and are determined directly by *V*_M_, by the product of an activation gate (*m*) and an inactivation gate (*h*), or by a set of coupled ODEs connecting closed (C_X_), open (O_X_) and blocked (B_X_) states. *C*: emerging approaches for modeling channel gating based on protein structures. *C* was adapted from Refs. 75 and 76, with permission from *Proceedings of the National Academy of Sciences USA* and *Biophysical Journal*, respectively.

Since different regions of the heart exhibit distinct electrophysiological properties, several families of models have been developed for specific regions (see sect. 2.3), each with its own applications (detailed in sect. 2.4). Typically, these models generate an AP by simulating dynamic changes in the underlying ionic currents, the sum of which determines the change in cardiomyocyte membrane potential (FIGURE 3*A*). On the other hand, phenomenological implementations that only aim to reproduce the AP shape and duration, without explicitly simulating underlying ion channels, have also been proposed (77, 78). These phenomenological models are significantly more computationally efficient, facilitating organ-level simulations or comparisons of numerous simulated conditions, and have been shown to generate spiral wave dynamics comparable to detailed ionic models when parametrized appropriately (77, 78). However, known disease-related ionic remodeling or drug effects cannot be directly incorporated in these phenomenological models, as they do not explicitly represent the components that are altered in disease, potentially hindering mechanistic interpretation.

For mechanistic models with detailed ion current formulations, each individual ion current can be modeled in several ways (FIGURE 3, *B* AND *C*). The most common approaches are *1*) instantaneous currents, modeled as a direct function of membrane potential and/or other physiological quantities captured by the model’s state variables [e.g., Ca^2+^ transient ([Ca^2+^]) or [Na^+^]]; *2*) Hodgkin–Huxley-type models of independent dynamic channel gates, named after the seminal work by Alan Hodgkin and Andrew Huxley (29); and *3*) Markov models representing the transitions between different channel states (typically representing different conformational changes of the macromolecular complex). Each approach relies on several simplifying assumptions that should be considered when developing an ion channel model and interpreting its results.

Instantaneous ion channel models assume that the channel’s kinetics are sufficiently rapid compared with changes in membrane potential (or other relevant state variables controlling channel gating) that any time delays due to channel gating can be ignored. The resulting ion current is a direct function of one or more state variables at that moment in time (i.e., for that specific state vector; FIGURE 3*B*). Instantaneous models are commonly used for inward-rectifier K^+^ currents (e.g., *I*_K1_, acetylcholine-activated *I*_K,ACh_, or adenosine triphosphate-sensitive *I*_KATP_), where the ion current is calculated as the product of the maximal conductance (reflecting the number of channels and single-channel conductance), a voltage-dependent function that describes the channel’s current-voltage relationship observed experimentally, and the driving force. The driving force is typically modeled as the difference between the reversal potential (which can be calculated based on the ionic concentrations inside and outside the cell) and the current membrane potential (FIGURE 3*A, bottom*). In addition, this approach is used for constitutively active background currents, where open probability is equal to 1 and current is determined by maximal conductance and driving force. However, most cardiac ion channels exhibit relevant and potentially complex dynamics, as the channel macromolecular complex undergoes multiple conformational changes in response to changes in membrane potential. These cannot be modeled with instantaneous ion channel models, instead requiring Hodgkin–Huxley or Markov model approaches.

Some channels, such as the ultrarapid delayed-rectifier K^+^ channel or the fast Na^+^ channel, activate within milliseconds, whereas others (e.g., the slow delayed-rectifier K^+^ channel) only fully activate during second-long depolarizing pulses. Similarly, some currents exhibit pronounced inactivation (e.g., *I*_Na_ or *I*_to_), giving rise to a transient current despite sustained voltage-dependent activation, whereas others produce a persistent current. The Hodgkin–Huxley approach models such dynamics as independent processes captured by one or more gating variables. These variables can be visualized as a series of gates. Ions can only enter or leave the cell if all gates are at least partially open. Each gate can transition between two states (e.g., open/closed, activated/deactivated, or available/inactivated), with the forward and backward transition rates typically dependent on membrane potential or intracellular ion concentrations (FIGURE 3*B, middle*). The value of each gating variable ranges between 0 and 1, reflecting the fraction of channels with their gating variable in that specific state. Each gate operates independently, and the channel open probability is given by the product of all gating variables. For example, a Hodgkin–Huxley model with two gating variables reflecting activation and inactivation can only conduct a current if both the activation gating variable is nonzero (representing a fraction of channels that have activated) and the inactivation gating variable is nonzero (i.e., not all channels have inactivated).

The goal of a Hodgkin–Huxley model is to reproduce the biophysical properties of an ion channel characterized by patch-clamp experiments (see sect. 2.2.3). Various approaches can be used to integrate biophysical knowledge into the model structure and facilitate a better correspondence between model and experimental data. For example, for each gate, the forward and backward rates can also be given as steady-state values and time constants. This formulation is mathematically equivalent but enables a much more direct comparison with experimental data, which are often presented as steady-state voltage dependence and time constants of (in)activation rather than rates of change. Similarly, products of the same gating variable can be used to reflect a steep dependence, typically associated with cooperative gating of multiple channel subunits. For example, K^+^ channels often consist of four identical subunits and are therefore sometimes modeled using a fourth-order (*n*^4^) dependence of the primary gating variable (69). Furthermore, additional gating variables can be added to enable independent control of the onset and recovery of a gating process. For example, most Hodgkin–Huxley formulations of the Na^+^ current employ one activation gate (*m*) and two gates to control inactivation and recovery from inactivation (often labeled *h* and *j*). These latter two gates typically have the same voltage-dependent steady state but different time constants. The gate with the fastest time constants at a given membrane potential will largely control the rate of inactivation (as it rapidly approaches 0, decreasing the value of the product of the 3 gates). Although the slowest time constant will take longer to reach a fully inactivated state, it will also take longer to become available again, thus keeping the product of the three gates small for a prolonged period of time and effectively controlling the speed of recovery from inactivation. Thus, by combining different gates with the right open/closing rates, Hodgkin–Huxley models can reproduce the biophysical properties of many ion channels. However, the independent nature of the processes in a Hodgkin–Huxley model precludes incorporation of state-dependent effects (e.g., a drug that can only bind in the open state). Such processes can be simulated with Markov models.

Each Hodgkin–Huxley model can be reformulated as an equivalent Markov model by combining the two states of each gating variable in the Hodgkin–Huxley formulation (79). For example, the Hodgkin–Huxley model with independent activation and inactivation processes can be represented with a four-state square Markov model in which the left-right transitions reflect activation/deactivation and the top-down transitions reflect inactivation/recovery. Because these processes were independent in the original Hodgkin–Huxley model, the top-down transitions are the same for the left and right columns (i.e., the rate of inactivation is the same whether the channel is in the closed or open state). Similarly, the left-right transitions are the same for the top and bottom row of the model. However, Markov models are a generalization of Hodgkin–Huxley models and are therefore able to represent more complex structures in which state transitions are dependent on the current state of the channel. For example, Markov models can readily capture state-dependent drug binding, in which a drug only binds when the channel is in the open state. Nevertheless, this increased versatility also creates challenges with respect to model identification and parameter estimation (discussed in sect. 2.2.3).

In whole cell models, Markov models are typically simulated as deterministic models represented by a system of ordinary differential equations equal to the number of states in the Markov model. The value of each variable represents the fraction of channels in that state at a given moment in time, and the change in each variable is determined by the product of the rates into a given state and the occupancy of the neighboring states, as well as the rates going out of that state (FIGURE 3*B, right*). By definition, each variable must be nonnegative and the sum over all Markov model states must equal 1. In addition, for each loop in the Markov model, the product of the rates in clockwise direction should equal the product in counterclockwise direction (a criterion called microscopic reversibility) based on energetic considerations. This deterministic implementation effectively assumes the presence of a large number of channels, such that the open probability can be determined with arbitrary precision. Alternatively, a Markov model can be used to simulate stochastic single-channel gating of a single or limited number of ion channels. In this case, each state variable reflects the absolute number of channels in that state and stochastic processes determine the number of channels that transition to a different state in a given time step. These stochastic simulations can be compared to experimental single-channel recordings and have been used to simulate the stochastic nature of subcellular Ca^2+^ handling abnormalities (sparks and waves) (80–83) and to investigate the impact of ion channel stochastics on beat-to-beat variation in repolarization of the cardiac AP (83).

Although Markov models can become quite complex, with more than a dozen states and even more parameters controlling state transitions, the aforementioned approaches nevertheless represent a dramatic simplification of the molecular dynamics of cardiac ion channel macromolecular complexes, which involve numerous proteins made up of hundreds of amino acids and in principle an infinite number of conformations in three-dimensional (3-D) space. Crystallography and cryo-electron microscopy, together with advances in computational approaches for protein structure prediction including homology modeling and machine learning approaches such as AlphaFold, have resulted in a growing number of increasingly detailed protein structures (84). These protein structures provide the basis for molecular dynamics (MD) simulations that aim to calculate the position of each atom as a function of time based on physical laws, ultimately reflecting conformational changes (85, 86). Traditional MD simulations are unable to capture the timescales required for simulating large conformational changes such as voltage-dependent channel opening. However, previous work by Silva et al. (75) has employed MD simulations and the Poisson–Boltzmann equation to derive electrostatic energy landscapes as a function of movement of the S4 transmembrane segment of KCNQ1, the pore-forming subunit of the slow delayed-rectifier K^+^ current (*I*_Ks_) channel. This energy landscape and its voltage dependence can then be used in Monte Carlo simulations involving repeated random sampling of trajectories constrained by the energy landscape. The resulting information on gating probabilities serves as input for a Markov model of channel opening (FIGURE 3*C, left*) (75). More recent work has extended this approach by using machine learning approaches or a library of channel conformations and corresponding electrostatic energies of the *I*_Ks_ channel to allow simulation of trajectories of conformational changes at a finer resolution, without assumptions about the tetrameric symmetry of the channel and in the presence of the KCNE1 beta subunit, and use these to determine macroscopic ion currents (FIGURE 3*C, right*) (76, 87). These protein structure-based approaches in theory enable direct mechanistic simulations of the functional effects of mutations in cardiac ion channels (discussed in sect. 2.4.3).

#### 2.2.2. Simulating ionic homeostasis.

Although initial AP models only included electrical (ion channel) components (30, 31), modern models also simulate changes in ion concentrations resulting from ion transport through ion channels, pumps, and exchangers (33). Most models consider intracellular changes in Ca^2+^, Na^+^, and K^+^, with a few, such as the Hund–Rudy model of the canine ventricular cardiomyocyte (88), also incorporating dynamic changes in Cl^−^. Incorporation of changes in ion concentrations greatly augments the temporal dynamics of cardiac electrophysiology models. For example, changes in pacing rate have immediate effects on the cardiac AP because of restitution properties that modulate the number of available ion channels on a beat-to-beat basis depending on the length of the preceding diastolic interval. On the other hand, intracellular Na^+^ accumulation over seconds to minutes contributes to slow accommodation of the simulated cardiac AP (89, 90). Accordingly, long periods of prepacing are often required to achieve steady-state results in modern models incorporating dynamic changes in ion concentrations. Charge conservation (i.e., that every current flowing through the membrane results in a corresponding change in ionic concentrations) needs to be taken into account in these models to ensure convergence to a steady state (91, 92). This requires ensuring that all currents through the membrane, including the stimulus current, are incorporated in the concentration updates (91). Moreover, charge conservation principles enable an algebraic formulation of the membrane potential based on intracellular and extracellular ion concentrations. This approach improves model accuracy and makes explicit the assumptions on charges that modulate membrane potential but are not considered in the model, which are normally hidden in the initial conditions (93). Although perhaps somewhat technical in origin, several studies have shown that these aspects related to simulating ionic homeostasis affect model stability, drift, uniqueness of model predictions, and bias in parameter estimation (91–93), thereby potentially having important implications for the validity of their clinical applications.

To simulate the Ca^2+^-induced Ca^2+^ release process responsible for excitation-contraction coupling, most cardiomyocyte models available to date contain various compartments for different intracellular domains (e.g., the SR, subsarcolemmal space, or junctional space between sarcolemma and SR) with either free diffusion of ions between spaces or ion release and transport via intracellular channels and pumps. Most models only contain a single copy of each of these compartments, reflecting the aggregate behavior throughout the entire cardiomyocyte in a so-called “common pool” structure (37). However, Ca^2+^ handling is a highly localized subcellular process, and common pool models cannot readily capture the strong local feedback processes that result in high gain and gradedness of Ca^2+^-induced Ca^2+^ release. Models that explicitly simulate the local control of this process by dividing the virtual cell into local Ca^2+^ domains with stochastic gating of L-type Ca^2+^ channels and ryanodine receptors more faithfully reproduce experimentally observed Ca^2+^ handling properties and can also simulate the occurrence of proarrhythmic spontaneous SR Ca^2+^ release events (37). Moreover, local alterations in parameters of these spatial Ca^2+^ handling models make it possible to simulate variations in subcellular structure such as the absence or presence of axial or t-tubular structures (82). This approach has been extended to high-resolution reconstructions of subcellular structures based on serial block-face scanning electron microscopy of sheep ventricular samples (94). Such models enable the characterization of the cellular proarrhythmic consequences of subcellular remodeling (see sect. 2.4.1) However, spatial Ca^2+^ handling models have several orders of magnitude higher computational complexity than common pool models. Although this precludes their direct incorporation into multicellular organ-scale models, modeling approaches have been proposed to phenomenologically capture some of the aspects of spontaneous SR Ca^2+^ release events in common pool models, enabling investigations of the interplay between ectopy and reentry in multiscale simulations (95).

#### 2.2.3. Model calibration, validation, and uncertainty quantification.

During model development, it is common to first perform parameter identification for individual ion channels based on whole cell voltage-clamp experiments. These experiments provide well-defined and tightly constrained conditions, making it possible to estimate the parameters of individual ion channel models in isolation. Subsequently, more global parameters that cannot be constrained by a single set of experiments are estimated based on whole cell behavior integrating the effects of all ion currents (e.g., rate dependence of AP duration or Ca^2+^ transient).

Fitting ion channel models remains, however, a complex task. Most traditional approaches compare simulated and experimental time constant, steady-state, or current-voltage curves, as this is straightforward to perform with available data from the literature. However, by comparing four different methods to fit the *I*_Kr_ current, Clerx et al. (96) showed that these traditional methods perform poorly compared with fitting the model to raw traces obtained with different voltage-clamp protocols. Moreover, tailored voltage-clamp protocols consisting of a mix of rapid fluctuations also enabled accurate parameter estimation with much shorter protocols. This interplay between model and experimental data is also important for validation of cellular models. For cell models, most efforts are currently focusing on “general validation” of the model, comparing general output quantities to human experimental data. Recent efforts have strengthened the credibility of electrophysiology models by performing “true” validation using data not employed in model calibration (73). Credibility and generalization capabilities of the model were strengthened by showing that it was consistent with a large range of behaviors observed in experimental studies, despite not being optimized for these specifically. These results highlight the importance of sharing electrophysiological datasets according to FAIR (findable, accessible, interoperable, and reusable) standards to enable better model calibration and validation in the future.

Model calibration and validation also face challenges caused by physiological variability (discussed in sect. 2.2.4), sparsity of data, and species differences. This lack of information, called uncertainty, may arise from various sources such as experimental measurements (patient-specific and species differences, experimental conditions), parameter estimation (identifiability, calibration), structure of the model (level of detail, assumptions), or numerical methods (solver, approximations) (97). Methods to quantify this uncertainty are available, such as Monte Carlo simulations, Latin hypercube sampling, or more novel data-driven approaches, providing a distribution of predictions that accounts for this uncertainty, instead of traditional point predictions (98). The use of more complete datasets to calibrate models was shown to not necessarily reduce the uncertainty in parameter estimation. Conversely, using less complex models allowed a better fit and therefore performance of the model (99). Uncertainty can strongly influence the outcome of the computer models, affecting for example AP and Ca^2+^ transient properties, rate dependence, reentrant wave dynamics (77), or the occurrence of alternans (100). Understanding and quantifying this uncertainty is therefore crucial to ensure credibility and reproducibility of the models (101), especially if used for safety-critical decision making (102). Patient-specific adjustments of the model parameter set are key to ensure patient-specific mechanistic predictions (78). A complete discussion on model calibration and validation is beyond the scope of this review, but the interested reader is referred to more extensive reviews on this topic such as Refs. 103, 104.

#### 2.2.4. Variability and the population-of-models approach.

Variability is present in all biological systems. Even cardiomyocytes from healthy animal models with a relatively homogeneous genetic background show considerable cell-to-cell variability (105), which is further exacerbated in cardiomyocytes from humans with variable genetic background and disease-related remodeling (106). This biological variability contrasts with the traditional computational modeling approach that produces a single average virtual cardiomyocyte. However, an ensemble of average data may not accurately represent any individual cardiomyocyte (107). To address this limitation, the population-of-models approach has become widely used in electrophysiology modeling to incorporate cell-to-cell and interindividual variability (108). By varying a set of model parameters (typically maximal channel conductances), a population of cell models is constructed, each with slightly distinct electrophysiological properties. This population is then calibrated to experimental reference data at the whole cell level (e.g., AP properties) and/or the level of individual ionic currents (109, 110). Other approaches include Bayesian calibration (101) or Gaussian process emulators (111). The resulting cell model population can be used to characterize mechanisms that are less dependent on the specific parametrization of a single model and perform a sensitivity analysis to identify the relative contribution of individual ionic components to a specific output parameter through multivariable regression (112) (discussed in sect. 2.4.1). In addition, populations of models can be used to predict the likelihood of a response by assessing the number of models in a population with a specific phenotype. This approach can help to assess variability in drug response and contribute to risk assessment, e.g., about drug-induced proarrhythmia (107, 108, 113) (discussed in detail in sect. 2.4.4). Accounting for physiological variability with modeling approaches such as populations of models therefore strengthens the generalization of model predictions in the context of drugs and disease and improves our understanding of biological variability.

### 2.3. Major Families of Human Cardiomyocyte Models for Different Cardiac Regions

#### 2.3.1. Sinoatrial node.

**FIGURE 4.**
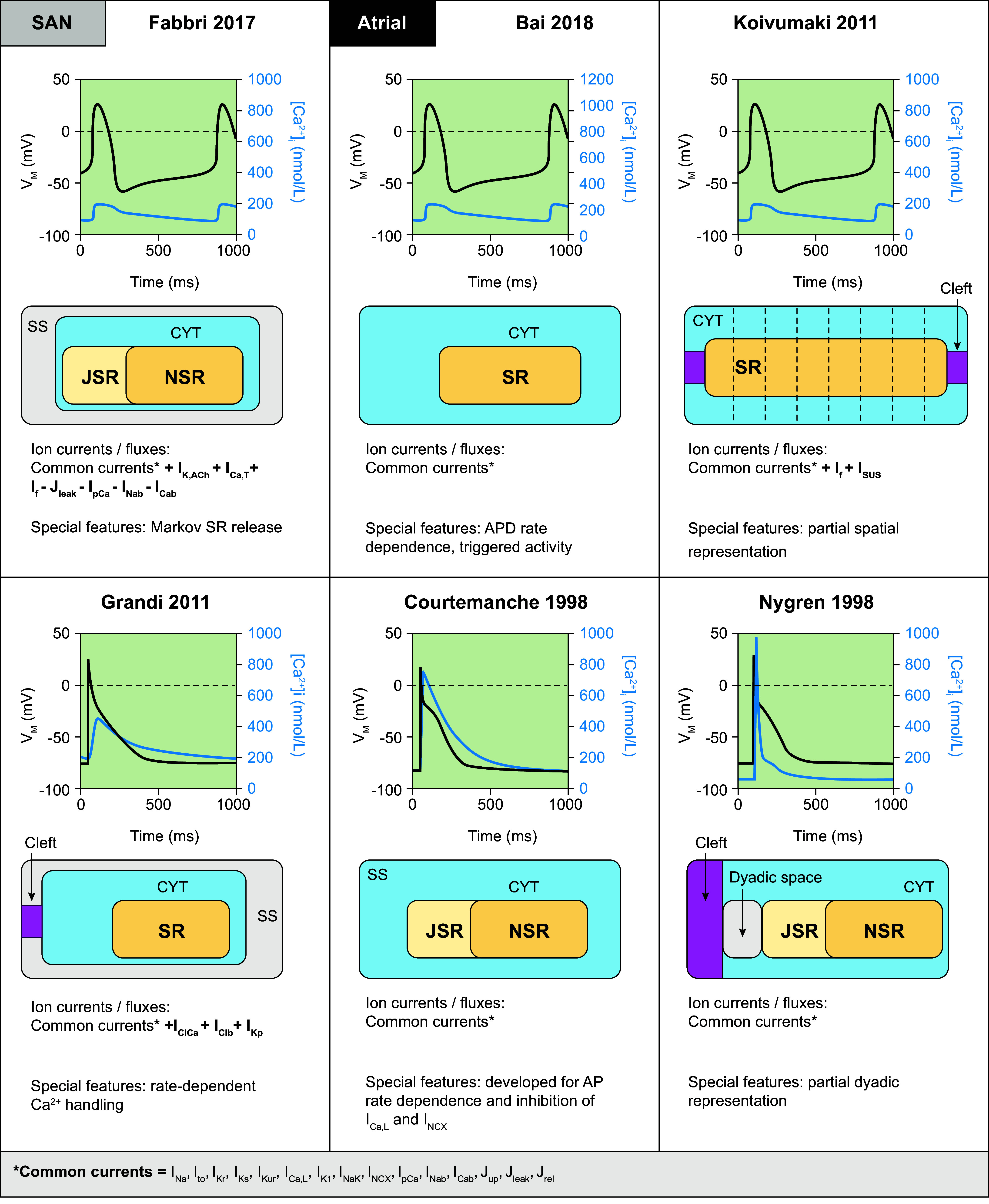
Primary human sinoatrial node (SAN) and atrial cardiomyocyte and sinoatrial node models. Simulated action potentials [transmembrane voltage (*V*_M_)] and Ca^2+^ transients ([Ca^2+^]_i_), model structure, simulated ion currents and fluxes, and special features are highlighted. Traces were simulated using the human SAN cell models from Fabbri 2017 (120), as well as models from Bai 2018 (121), Grandi 2011 (90), Koivumäki 2011 (121,122) Courtemanche 1998 (45), and Nygren 1998 (44, 118). Models were obtained from https://www.cellml.org/ or implemented based on the model equations. AP, action potential; APD, action potential duration; CYT, cytosol; I_Cab_, background Ca^2+^ current; *I*_Ca,L_, L-type Ca^2+^ current; *I*_Ca,T_, T-type Ca^2+^ current; *I*_Clb_, background Cl^−^ current; *I*_ClCa_, Ca^2+^-dependent Cl^−^ current; *I*_f_, hyperpolarization-activated cyclic nucleotide-gated “funny” current; *I*_K1_, basal inward-rectifier K^+^ current; *I*_K,ACh_, acetylcholine-activated inward-rectifier K^+^ current; *I*_Kp_, plateau K^+^ current; *I*_Kr_, rapid delayed-rectifier K^+^ current; *I*_Ks_, slow delayed-rectifier K^+^ current; *I*_Kur_, ultrarapid delayed-rectifier K^+^ current; *I*_Na_, Na^+^ current; *I*_Nab_, background Na^+^ current; *I*_NaK_, Na^+^-K^+^-ATPase current; *I*_NCX_, Na^+^/Ca^2+^ exchange current; *I*_pCa_, plasmalemmal Ca^2+^-ATPase current; *I*_sus_, sustained K^+^ current; *I*_to_, transient outward K^+^ current; J_leak_, Ca^2+^ leak from sarcoplasmic reticulum; J_rel_, Ca^2+^-release flux from the sarcoplasmic reticulum; JSR, junctional sarcoplasmic reticulum; J_up_, Ca^2+^ uptake flux into the sarcoplasmic reticulum; NSR, network sarcoplasmic reticulum; SR, sarcoplasmic reticulum; SS, subspace Ca^2+^ domain.

The sinoatrial node is the intrinsic pacemaker of the heart. Its cells depolarize spontaneously to produce APs, setting the rhythm of the heart under control of autonomic regulation. Hyperpolarization-activated cyclic nucleotide-gated (HCN) channels permeable to sodium and potassium ions contribute under physiological conditions to a depolarizing funny current (*I*_f_). Together with Ca^2+^ influx through T-type and L-type Ca^2+^ channels, this causes the membrane potential to gradually increase, contributing to the autonomous pacemaker activity (voltage clock). Spontaneous Ca^2+^ releases from the SR also gradually increase intracellular Ca^2+^ levels, leading to an increase in membrane potential (through the NCX). This mechanism is referred to as the Ca^2+^ clock (63). The first generic SAN models were developed in the 1980s by modifying the equations of earlier cardiomyocyte models. In the 1990s SAN single-cell models based on animal data were developed, which focused primarily on the HCN-driven membrane clock. Over time, these models evolved toward a coupled clock system, incorporating both HCN-driven and Ca^2+^-driven mechanisms. In addition, various models have implemented the signaling cascades underlying autonomic regulation of heart rate [see Wilders (114) and Ricci et al. (115) for overviews of SAN models from before and after 2007, respectively]. However, until 2017 most experimental data on SAN electrophysiology had been obtained from animals, making the development, calibration, and application of SAN computer models to study pacemaking and sinus node disease in humans difficult. Two models by Seemann et al. (116) and Chandler et al. (117), developed in 2006 and 2009, respectively, were primarily used, but their applicability and similarities with human electrophysiology were limited. In 2016, Pohl et al. (118) incorporated the data on human SAN cells from Verkerk et al. (119) into a rabbit model to study parasympathetic regulation of heart rate. This model reproduced experimental basal cycle length duration and its regulation by acetylcholine but not AP characteristics. In 2017, Fabbri et al. (120) provided, for the first time, a model strictly based on available human cellular electrophysiological data, which has subsequently undergone refinement of intracellular ion homeostasis and refitting of the properties of *I*_f_ (115). The cellular structure and ion channels incorporated in this model are presented in FIGURE 4, along with its AP and intracellular Ca^2+^ transient.

#### 2.3.2. Atrial cardiomyocytes.

Atrial cardiomyocytes exhibit important electrophysiological differences compared with SAN cells. They express fewer HCN channels, a larger inward *I*_Na_, and a prominent *I*_K1_, resulting in a more stable resting membrane potential and rapid conduction. However, atrial *I*_K1_ is nevertheless smaller than in ventricular cells, resulting in an intermediate resting membrane potential and slower phase 3 repolarization (123). Ca^2+^ handling also differs in atrial cells, with increased SERCA2a expression and reduced expression of cardiac ryanodine receptors and calsequestrin compared with ventricular cells. Finally, atrial cells also have less well-developed t tubules than ventricular cardiomyocytes, which leads to a more heterogeneous Ca^2+^ release (124).

Given the clinical relevance of AF and the fact that human atrial tissue is among the most readily available cardiac tissue in patients undergoing open heart surgery, providing a rich experimental dataset on human atrial cellular electrophysiology, many computational models of human atrial cellular electrophysiology have been developed over the years. In 1998, the availability of human data led to the development of the two first human atrial models by Courtemanche et al. (45) and Nygren et al. (44). Both models are common pool models with a homogeneous cytosolic compartment that have provided important insights in the dynamics of atrial electrophysiology and are still widely used, but they did not consider remodeling of intracellular Ca^2+^ handling, which has since then emerged as a major arrhythmogenic mechanism in AF (124). In 2011, the Grandi et al. (90) and Koivumäki et al. (122) models were the first to model atrial Ca^2+^ handling in greater detail. The Grandi model retained a common pool architecture but provided a detailed calibration of rate‐dependent Ca^2+^ handling, including the effects of beta-adrenergic stimulation (90). At the same time, the Koivumäki model provided the first model including a partial spatial representation of the atrial cardiomyocyte (122). More recently, the model by Bai et al. (121) improved AP duration (APD) restitution curves and allowed the simulation of early and delayed afterdepolarizations. The cellular structure and ion channels incorporated in these models are presented in FIGURE 4, along with their APs and Ca^2+^ transients. Of note, although all models employ data from human atrial cardiomyocytes for most ion currents, including several common datasets, these models exhibit notable differences in AP rate dependence, resulting in distinct properties of reentrant activity in tissue simulations (125). Moreover, several modifications have been developed to address specific research questions or enrich the original models after the acquisition of novel experimental data. These models and their specific characteristics are summarized in TABLE 1.

**Table 1.** Overview of modifications to the main human atrial cardiomyocyte models

Model	Innovation from Original Model and Purpose
*Original model: Grandi (90)*	
Bai 2021 (126)	• Addition of *I*_K,ATP_ and *I*_K,ACh_ • Comparison between modified Grandi and Bai models
Colman 2018 (127)	• Calibration with new data (from 1 dataset) • New formulations of *I*_K1_, *I*_hyp_, *I*_to_, *I*_sus_, *I*_CaL_
Ellinwood 2017 (128)	Markov formulation of *I*_Kur_
Onal 2017 (129)	Addition of *I*_NaL_ and CaMKII signaling
Voigt 2014 (130)	Spatial/stochastic gating of RyR2
Voigt 2013 (131)	Na^+^-dependent regulation of *I*_K1_ and *I*_K,ACh_
Schmidt 2015 (62)	Formulation of the K2P3.1 current
*Original model: Koivumäki (122)*	
Skibsbye 2016 (132)	• Reformulation of *I*_Na_ • Adjustment of *I*_to_, *I*_CaL_ • Addition of *I*_KCa_
Koivumäki 2014 (133)	• Reformulation of *I*_CaL_ (increased contribution of Ca^2+^-dependent vs. voltage-dependent inactivation) • Update of SERCA2a parameters
*Original model: Bai (121)*	
Bai 2021 (126)	• Addition of *I*_K,ATP_ and *I*_K,ACh_ • Comparison between modified Grandi and Bai models
*Original model: Courtemanche (45)*	
Bai 2019 (134)	New *I*_NaL_ current based on Grandi 2011 (90)
Colman 2018 (127)	• Calibration with new data (from 1 dataset) • New formulations of *I*_K1_, *I*_hyp_, *I*_to_, *I*_sus_, *I*_CaL_ • Comparison between different models
Vincenti 2014 (135)	• Dependence of *I*_K1_ and *I*_Kr_ currents on [K^+^]_o_ • Improved long-term stability • Addition of *I*_K,ACh_
Li 2007 (136)	Addition of *I*_Anion_ (anionic background current)
Tsujimae 2007 (137)	Additional slow activation process for *I*_Ks_
Colman 2013 (138)	• New formulation of *I*_to_, *I*_Kur_, intracellular calcium handling • Addition of 2 compartments in the SR
Colman 2017 (139)	New *I*_Kur_ formulation
Ni 2017 and 2019 (140)	• Adjustment of *I*_CaL_ baseline conductance to reduce systolic calcium levels • Addition of *I*_Kur_ • Addition of *I*_NaL_
*Original model: Nygren (39)*	
Colman 2018 (127)	• Calibration with new data (from 1 dataset) • New formulations of *I*_K1_, *I*_hyp_, *I*_to_, *I*_sus_, *I*_CaL_ • Comparison between different models
Maleckar 2008 (141)	New repolarization model

CaMKII, Ca^2+^/calmodulin-dependent protein kinase II; *I*_Ca,L_, L-type Ca^2+^ current; *I*_hyp_, hyperpolarization-activated current; *I*_K1_, basal inward-rectifier K^+^ current; *I*_K,ACh_, acetylcholine-activated inward-rectifier K^+^ current; *I*_K,ATP_, ATP-sensitive K^+^ current; *I*_KCa_, Ca^2+^-activated K^+^ current; *I*_Ks_, slow delayed-rectifier K^+^ current; *I*_Kur_, ultra-rapid delayed-rectifier K^+^ current; *I*_Na,L_, L-type Na^+^ current; *I*_sus_, sustained K^+^ current; *I*_to_, transient outward K^+^ current; [K^+^]_o_, extracellular potassium concentration; RyR2, ryanodine receptor channel type 2; SR, sarcoplasmic reticulum.

#### 2.3.3. Purkinje cells.

**FIGURE 5.**
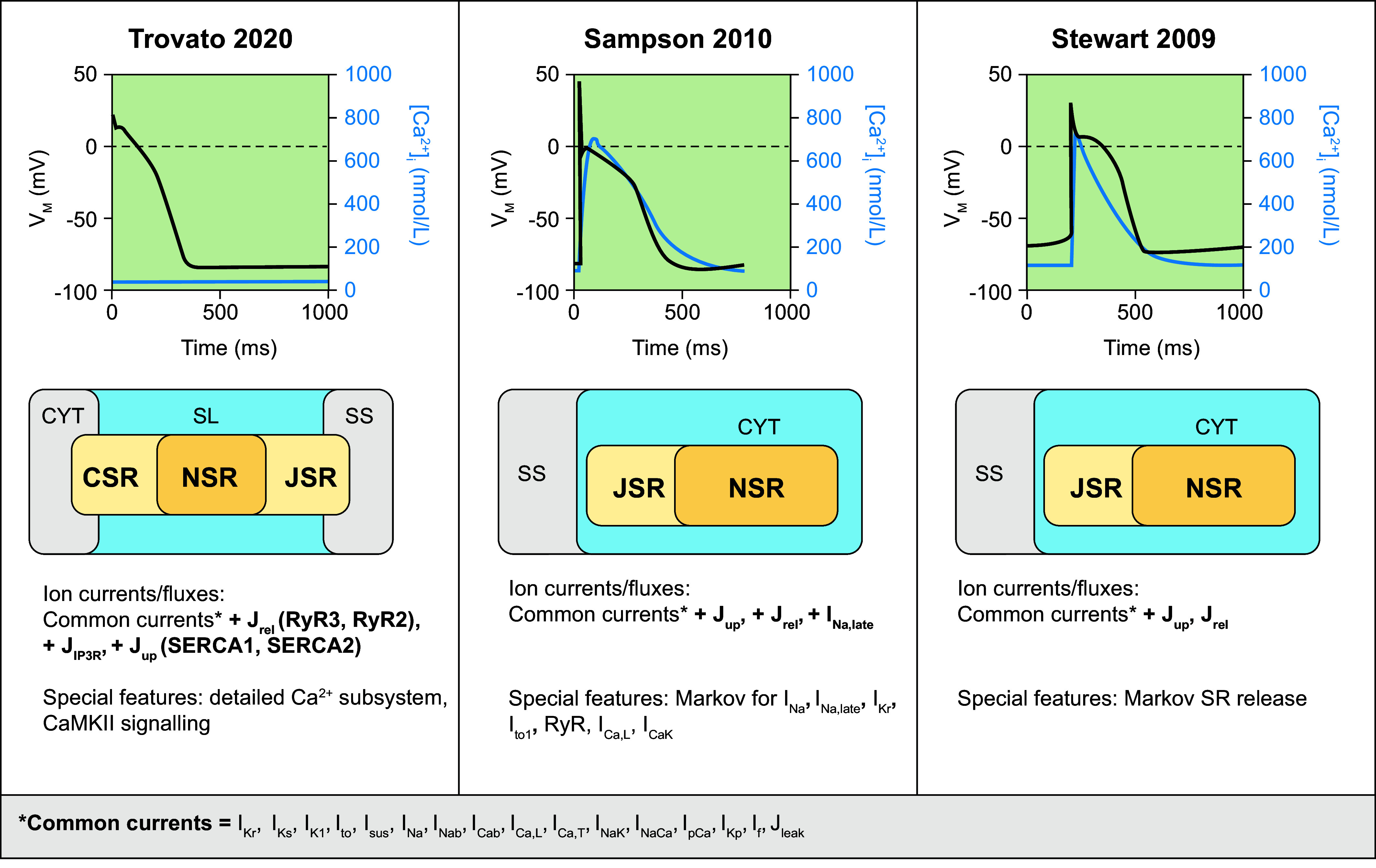
Primary human Purkinje cell models. Simulated action potentials [transmembrane voltage (*V*_M_)] and Ca^2+^ transients ([Ca^2+^]_i_), model structure, simulated ion currents and fluxes, and special features are highlighted. Traces were simulated with human Purkinje cell models from Trovato 2020 (146), Sampson 2010 (145), and Stewart 2009 (143). Models were obtained from https://www.cellml.org/ or implemented based on the model equations. CaMKII, Ca^2+^/calmodulin-dependent protein kinase II; CSR, corbular sarcoplasmic reticulum; CYT, cytosol; *I*_Cab_, background Ca^2+^ current; *I*_CaK_, K^+^ flux through the L-type Ca^2+^ channel; *I*_Ca,L_, L-type Ca^2+^ current; *I*_Ca,T_, T-type Ca^2+^ current; *I*_f_, hyperpolarization-activated cyclic nucleotide-gated “funny” current; *I*_K1_, basal inward-rectifier K^+^ current; *I*_Kp_, plateau K^+^ current; *I*_Kr_, rapid delayed-rectifier K^+^ current; *I*_Ks_, slow delayed-rectifier K^+^ current; *I*_Na_, Na^+^ current; *I*_Na,late_, persistent late Na^+^ current; *I*_Nab_, background Na^+^ current; *I*_NaK_, Na^+^-K^+^-ATPase current; *I*_NaCa_, Na^+^/Ca^2+^ exchange current; *I*_pCa_, plasmalemmal Ca^2+^-ATPase current; *I*_sus_, sustained K^+^ current; *I*_to_, transient outward K^+^ current; J_IP3R_, inositol trisphosphate receptor Ca^2+^ flux; J_leak_, Ca^2+^ leak from sarcoplasmic reticulum; J_rel_, Ca^2+^-release flux from the sarcoplasmic reticulum; JSR, junctional sarcoplasmic reticulum; J_up_, Ca^2+^ uptake flux into the sarcoplasmic reticulum; NSR, network sarcoplasmic reticulum; RyR(2/3), ryanodine receptor (type 2/3); SERCA(1/2), sarco(endo)plasmic reticulum Ca^2+^-ATPase (type 1/2); SL, subsarcolemmal space; SR, sarcoplasmic reticulum; SS, subspace Ca^2+^ domain.

After reaching the AV node, the electrical stimulus propagates rapidly through the His–Purkinje system to ensure a synchronous activation of the ventricles. To the best of our knowledge, there are at present no human-specific computational models for cells from the AV node or bundle of His, likely because of a paucity of experimental data from human hearts from this region. However, a few models exist for Purkinje cells. Purkinje cells exhibit a faster AP upstroke, a more negative plateau (due to a smaller *I*_Ca,L_), and a larger APD (due to slowly inactivating *I*_Na_) compared with ventricular cardiomyocytes (142). In 2009, Stewart et al. (143) developed a Purkinje cell model based on the ten Tusscher model of the human ventricular cardiomyocyte (144), adding two currents: a hyperpolarization-activated and a sustained K^+^ current. In 2010, the Sampson et al. model (145) recapitulated distinct electrophysiological characteristics of Purkinje cells, using Markov formulations for various Purkinje-specific channels. In addition to differences in ion channel expression, Purkinje cells also have a lower density of t tubules compared with ventricular cardiomyocytes, resulting in a large spatial calcium heterogeneity. More recently, the Trovato et al. model (146) incorporated for the first time Purkinje-specific ion currents and detailed Ca^2+^ handling. The model was calibrated based on nondiseased human data. Based on this novel calibration, the magnitude of the Ca^2+^ transient in the Trovato model is significantly lower than in previous models (FIGURE 5). The cellular structure and ion channels incorporated in these models are presented in FIGURE 5, along with their AP and Ca^2+^ transient.

#### 2.3.4. Ventricular cardiomyocytes.

**FIGURE 6.**
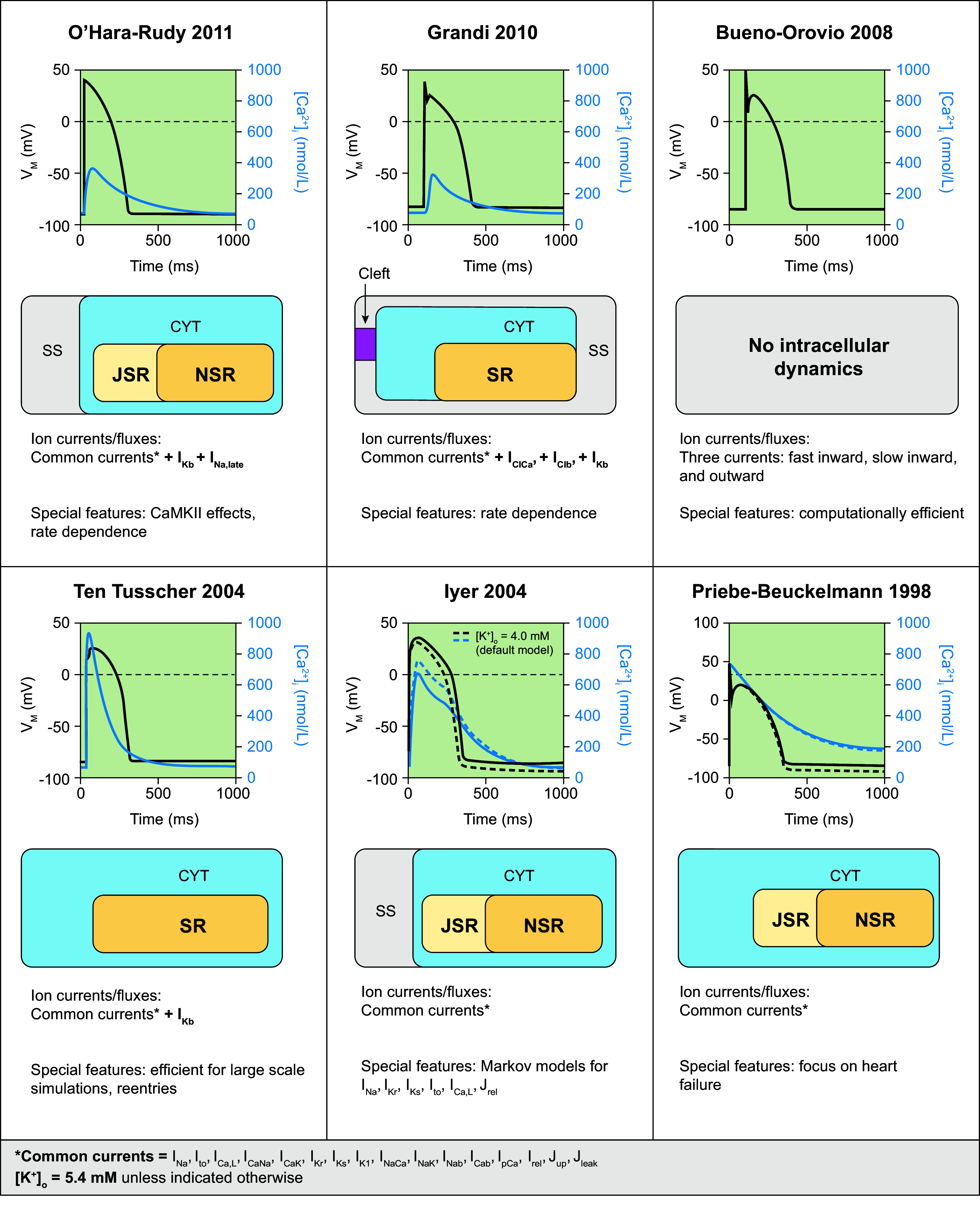
Primary human ventricular cardiomyocyte models. Simulated action potentials [transmembrane voltage (*V*_M_)] and Ca^2+^ transients ([Ca^2+^]_i_), model structure, simulated ion currents and fluxes, and special features are highlighted. Traces were simulated with the human ventricular cardiomyocyte models from O’Hara–Rudy 2011 (67), Grandi 2010 (147), Bueno-Orovio 2008 (77), ten Tusscher 2004 (144), Iyer 2004 (148), and Priebe–Beuckelmann 1998 (77, 149). Models were obtained from https://www.cellml.org/ or implemented based on the model equations. CaMKII, Ca^2+^/calmodulin-dependent protein kinase-II; CYT, cytosol; *I*_Cab_, background Ca^2+^ current; *I*_CaK_, K^+^ flux through the L-type Ca^2+^ channel; *I*_Ca,L_, L-type Ca^2+^ current; *I*_CaNa_, Na^+^ flux through the L-type Ca^2+^ current; *I*_Clb_, background Cl^−^ current; *I*_ClCa_, Ca^2+^-dependent Cl^−^ current; *I*_K1_, basal inward-rectifier K^+^ current; *I*_Kb_, background K^+^ current; *I*_Kr_, rapid delayed-rectifier K^+^ current; *I*_Ks_, slow delayed-rectifier K^+^ current; *I*_Na_, Na^+^ current; *I*_Na,late_, persistent late Na^+^ current; *I*_Nab_, background Na^+^ current; *I*_NaK_, Na^+^-K^+^-ATPase current; *I*_NaCa_, Na^+^/Ca^2+^ exchange current; *I*_pCa_, plasmalemmal Ca^2+^-ATPase current; *I*_to_, transient outward K^+^ current; J_leak_, Ca^2+^ leak from sarcoplasmic reticulum; J_rel_, Ca^2+^-release flux from the sarcoplasmic reticulum; JSR, junctional sarcoplasmic reticulum; J_up_, Ca^2+^ uptake flux into the sarcoplasmic reticulum; [K^+^]_o_, extracellular potassium concentration; NSR, network sarcoplasmic reticulum; SR, sarcoplasmic reticulum; SS, subspace Ca^2+^ domain.

The size of the ventricular myocardium with its extensive perfusion via the coronary arteries has facilitated the isolation of ventricular cardiomyocytes for cellular electrophysiology studies. Together with the significant societal impact of SCD due to ventricular arrhythmias, this has motivated the development of many ventricular cardiomyocyte models. Nevertheless, nondiseased human ventricular cardiomyocytes are difficult to obtain, limiting the number of human-specific ventricular cardiomyocyte models that are available to date. FIGURE 6 summarizes the subcellular structure and ion channels included in the most well-known human ventricular cardiomyocyte models together with their AP and Ca^2+^ transient morphology. Based on these key models, several modifications have been developed to address specific research questions or enrich the original models following the acquisition of novel experimental data. These models and their specific characteristics are described in TABLE 2.

Early work by Priebe and Beuckelmann (149) used the human data available at the time to develop a model for healthy and failing ventricular myocardium. Later, Iyer et al. (148) improved on the simulation of intracellular ion dynamics using Markov chain models for various channels, recapitulating APD frequency dependence and Ca^2+^-frequency relations. In the same year, ten Tusscher et al. (144) developed a model combining a high level of electrophysiological detail with high computational efficacy, allowing large-scale multicellular simulations. Despite some limitations, e.g., related to the balance of repolarizing K^+^ currents, with an overestimation of the role of *I*_Ks_ under basal conditions, this model remains commonly used for organ-level simulations. In 2008, a phenomenological implementation aiming to reproduce the AP shape and duration without explicitly simulating underlying ion channels was proposed by Bueno-Orovio et al. (77). This computationally efficient model facilitated organ-level simulations and was able to generate spiral wave dynamics comparable to detailed ionic models. In 2010, Grandi et al. (147) proposed a model with a different subcellular structure, including subsarcolemmal and junctional compartments. This model recapitulated APD rate dependence and produced a Ca^2+^ transient significantly different from earlier models (FIGURE 6). Finally, in 2011, the O’Hara–Rudy model (67) was published. This study provided new experimental data from nondiseased human ventricular samples that were used to develop a model that recapitulated many electrophysiological findings (including biophysical properties of all major ion channels and AP and Ca^2+^ transient morphology at different pacing frequencies, APD restitution properties, and alternans). Since then, this model has become the consensus base model for many cellular electrophysiological applications, such as the in silico prediction of drug effects (see sect. 2.4.4). A significant number of studies have built upon this model, mainly focusing on further improving the response to pharmacological interventions (156, 157) or issues related to excitability and propagation failure (153) (TABLE 2). In addition, for many modern human ventricular cardiomyocytes (e.g., the ten Tusscher, Grandi, and O’Hara models), both endocardial and epicardial (and sometimes midmyocardial) versions are available, based on experimentally characterized differences in ionic currents between the different transmural layers of the ventricle. Among other things, these model versions facilitate characterization of mechanisms of arrhythmias linked to specific regions, e.g., in the setting of Brugada syndrome, where arrhythmias originate primarily from the right ventricular (RV) epicardium (164).

Despite general similarities in baseline AP duration, these human ventricular cardiomyocyte models show notable differences in AP rate dependence and restitution, as well as conduction velocity, resulting in significant differences in reentrant activity produced by each model (171). As a result, different cellular electrophysiology models are most commonly employed for cellular versus organ-level studies.

#### 2.3.5. hIPSC-derived cardiomyocytes.

**FIGURE 7.**
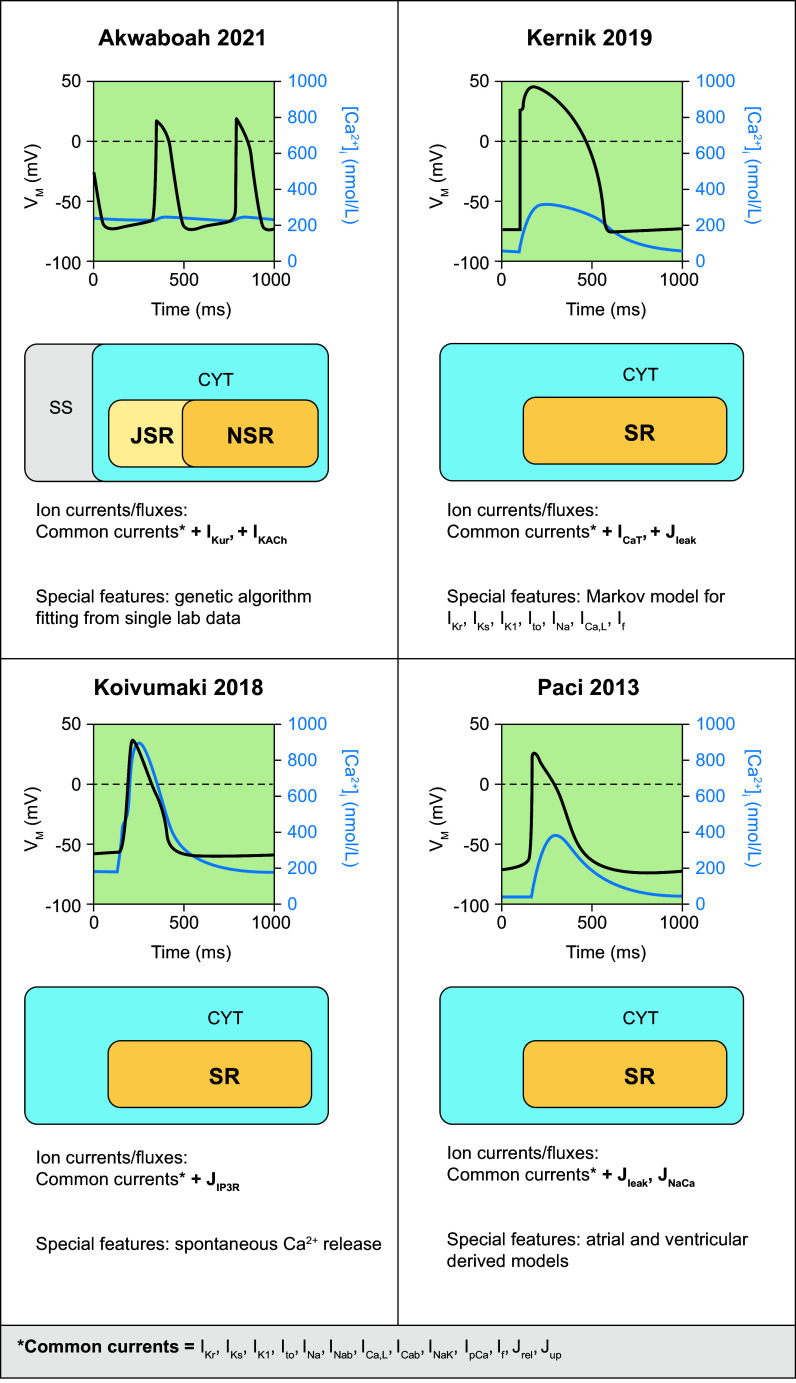
Primary human induced pluripotent stem cell-derived cardiomyocyte models. Simulated action potentials [transmembrane voltage (*V*_M_)] and Ca^2+^ transients ([Ca^2+^]_i_), model structure, simulated ion currents and fluxes, and special features are highlighted. Traces were simulated with models from Akwaboah 2021 (179), Kernik 2019 (178), Koivumäki 2018 (177), and Paci 2013 (174). Models were obtained from https://www.cellml.org/ or implemented based on the model equations. CYT, cytosol; *I*_Cab_, background Ca^2+^ current; *I*_Ca,L_, L-type Ca^2+^ current; *I*_Ca,T_, T-type Ca^2+^ current; *I*_f_, hyperpolarization-activated cyclic nucleotide-gated “funny” current; *I*_K1_, basal inward-rectifier K^+^ current; *I*_K,ACh_, acetylcholine-activated inward-rectifier K^+^ current; *I*_Kr_, rapid delayed-rectifier K^+^ current; *I*_Ks_, slow delayed-rectifier K^+^ current; *I*_Kur_, ultrarapid delayed-rectifier K^+^ current; *I*_Na_, Na^+^ current; *I*_Nab_, background Na^+^ current; *I*_NaK_, Na^+^-K^+^-ATPase current; *I*_NaCa_, Na^+^/Ca^2+^ exchange current; *I*_pCa_, plasmalemmal Ca^2+^-ATPase current; *I*_to_, transient outward K^+^ current; J_IP3R_, inositol triphosphate receptor Ca^2+^ flux; J_leak_, Ca^2+^ leak from sarcoplasmic reticulum; J_rel_, Ca^2+^ release flux from the sarcoplasmic reticulum; JSR, junctional sarcoplasmic reticulum; J_up_, Ca^2+^ uptake flux into the sarcoplasmic reticulum; NSR, network sarcoplasmic reticulum; SR, sarcoplasmic reticulum; SS, subspace Ca^2+^ domain.

Although not necessarily representing a distinct cardiac region, the advent of human induced pluripotent stem cell (hIPSC)-derived cardiomyocytes (hIPSC-CMs) has greatly facilitated studies of patient-specific cardiac cellular electrophysiology based on cells that carry the donor’s genetic information (172). Various protocols for the generation of cells reflecting characteristics of atrial, ventricular, or nodal cardiomyocytes have been developed. Despite significant advances, hIPSC-CMs remain highly heterogeneous with a relatively immature phenotype, exhibiting spontaneous beating, an immature cellular structure and Ca^2+^ handling, as well as electrical behavior more closely resembling the fetal electrophysiology (172, 173). Computer models of hIPSC-CM have therefore been developed to better understand and characterize this immature phenotype and help address the issue of variability within and between cell lines. One of the first hIPSC-CM models is the 2013 Paci et al. model (174), which proposed a ventricular-like and atrial-like model based on experimental hIPSC-CM data. It could recapitulate the spontaneously beating phenotype but was based on fairly limited data on hIPSC‐CM properties. Several updates have subsequently provided improved intracellular Na^+^ and Ca^2+^ handling in this model (175, 176). In 2018, the Koivumäki et al. model (177) added a realistic representation of Ca^2+^ dynamics, allowing better comparison between hIPSC-derived and adult ventricular cardiomyocytes. Shortly thereafter, Kernik et al. (178) proposed a novel model based on data obtained from several different experimental datasets, accounting for phenotypic variability in hIPSC-CM. This issue was also recently addressed by Akwaboah et al. (179), who used genetic algorithm fitting on data from a single center to formulate a more robust hIPSC-CM model. The cellular structure as well as a list of ion channels described by these models are presented in FIGURE 7, along with their APs and Ca^2+^ transients. Thus, although hIPSC-CM models have slightly different applications than computer models of adult human cardiomyocytes, they enable a better characterization of the properties and limitations of hIPSC-CMs, thereby facilitating their applications in translational research on patient-specific arrhythmia mechanisms and antiarrhythmic treatment, making these models a relevant component of this review on computational modeling of cardiac electrophysiology and arrhythmogenesis with a focus toward clinical translation.

### 2.4. Applications of Cardiac Cellular Electrophysiology Models

#### 2.4.1. Elucidating mechanisms of normal cardiac electrophysiology and arrhythmogenesis.

Two common uses of computer models in mechanistic studies are *1*) to assess whether the combined effect of a number of experimentally observed factors is sufficient to explain a certain phenotype and *2*) to determine the relative contribution of individual ion currents to a certain mechanism, often using population-of-models approaches. These approaches have been applied to numerous components of normal cardiac electrophysiology and various arrhythmogenic mechanisms, including mechanisms of pacemaking, regulation of AP rate dependence, cardiac alternans, and formation of early and delayed afterdepolarizations. Moreover, technological advances have enabled a hybrid experimental-computational “dynamic clamp” approach in which a computationally simulated conductance is injected in real time into isolated cardiomyocytes, resulting in changes in membrane potential that are immediately fed back to the model. This bidirectional connection between models and myocytes has, for example, been used to overcome the low expression of *I*_K1_ in hIPSC-CM (180) and to study how specific biophysical properties of an ion channel affect AP morphology and duration (181). Finally, computer models make it possible to explore a wide range of conditions, thereby potentially facilitating the design of better experimental protocols, contributing to the replacement, reduction, and refinement of experimental animal studies (182).

An example of the role of cellular computer models in elucidating mechanisms of normal cardiac physiology is their contribution to the long-standing debate surrounding the mechanisms of cardiac pacemaking. Cardiac automaticity was initially primarily attributed to the funny current (*I*_f_), and computer models showed that complete block of *I*_f_ resulted in loss of cell automaticity (183). The development of more complex SAN models simulating intracellular Ca^2+^ handling helped shape the hypothesis of a Ca^2+^ clock (due to Ca^2+^ release from the SR activating the NCX) and the idea that it works in synergy with the voltage clock (184). Subsequently, several studies have provided insight into the modulation of pacemaking by the autonomic nervous system (185), the structural determinants of pacemaker function in situ (186–188), and the effects of ion channel mutations on cardiac pacemaking (120).

Cell-level computer models have also provided insight into the mechanisms underlying proarrhythmic events such as afterdepolarizations or alternans. Early modeling work proposed recovery and reactivation of *I*_Ca,L_ as mechanisms underlying the formation of EADs and highlighted the role of *I*_Kr_ in rate dependence of these EAD events (189). Other models elucidated the role of repolarization reserve, the redundancy in repolarization resulting from the interaction between different K^+^ channels, in the genesis of EADs (190) as well as the quantitative requirements for EADs to propagate in tissue (191). With the development of models with more detailed subcellular structure and calcium handling, the role of loss of Ca^2+^-dependent inactivation of *I*_Ca,L_ in generating EADs was also shown. Similarly, models including a detailed description of intracellular Ca^2+^ handling have provided insight into the role of CaMKII and altered SR Ca^2+^ release in the occurrence of alternans in HF and other diseases (192, 193). Finally, local control models with spatial Ca^2+^ handling have provided insight into the subcellular determinants of DADs, which are challenging to study experimentally because of the absence of tools to alter ion channel distribution or subcellular structure in a controlled manner. These models have shown that the subcellular localization and heterogeneous distribution of ryanodine receptors (82), SR Ca^2+^-ATPase (194), and NCX all modulate the likelihood of DADs (195). These computational modeling results have important implications for future experimental characterization of disease-related Ca^2+^ handling remodeling, emphasizing a need for spatially resolved analyses.

The relative contribution of individual ionic currents to a certain mechanism can also be explored with populations of ionic models. This is typically done by generating a large population of models, selecting those models with or without a certain trait, and comparing their underlying ionic profile. This approach has, e.g., provided insight into the ionic determinants of EADs and repolarization failure due to hypertrophic cardiomyopathy (HCM)-associated electrical remodeling (153). In this study, a population of 30,000 cellular models was generated, resulting in a calibrated population of 9,118 models that were consistent with experimental data on human AP properties. Previously reported electrical remodeling associated with HCM was subsequently imposed through relative changes in expression of individual ion channels. The 752 out of 9,118 configurations with HCM-associated remodeling that showed EADs or repolarization failure exhibited markedly reduced *I*_Kr_ and larger *I*_Ca,L_ than the models with normal repolarization. Subsequent simulations artificially impeding the reactivation of L-type Ca^2+^ channels consistently eliminated all repolarization abnormalities in all 752 models, positioning *I*_Ca,L_ reactivation as primary causal driver of EADs in HCM. A similar approach has been used to generate calibrated populations of human atrial AP models in sinus rhythm versus chronic AF based on three different human atrial AP models (108). All AF populations showed similar variations in *I*_K1_, *I*_Kur_, and *I*_to_, consistent with experimentally observed AF-related remodeling. Subsequent analyses identified that intersubject variability in *I*_K1_ and Na^+^-K^+^-ATPase current (*I*_NaK_) determines variability in APD at 90% repolarization (APD_90_), variability in *I*_Kur_, *I*_Ca,L_, and *I*_NaK_ determines variability in APD_50_, and combined variability in *I*_to_ and *I*_Kur_ determines variability in APD_20_ (196). In a third example, populations of models have been used to identify the ionic determinants of different types of alternans (197), showing that “eye-type” alternans (i.e., alternans that disappears upon faster pacing) is associated with larger *I*_Ca,L_. This larger *I*_Ca,L_ promotes Ca^2+^ loading at fast rates to ensure that SR Ca^2+^ levels are sufficient to generate a normal Ca^2+^ transient during the next beat, despite short diastolic intervals. Recent work has expanded these population-of-models analyses to include variability in intracellular signaling controlling the posttranslational regulation of multiple ion channels (43).

#### 2.4.2. Predicting the proarrhythmic effects of ion channel mutations.

Mutations in genes coding for ion channels may have proarrhythmic consequences. However, understanding how these mutations lead to changes in protein levels and/or impaired ion channel function and their downstream clinical phenotypes is challenging. The biophysical consequences of a mutation have traditionally been studied in isolation with patch-clamp experiments in heterologous expression systems expressing the wild-type (WT) channel, the mutated channel, or a combination of both. However, these experiments cannot reveal the effect of a mutation on the AP or cellular proarrhythmic indicators. Computer modeling can help translate the biophysical effects of the mutation at the level of the ion channel to alterations in AP properties and ECG, revealing potential proarrhythmic mechanisms.

Several studies have modeled biophysical characteristics of Na^+^ channel mutations related to clinical syndromes like long QT syndrome (LQTS) type-3 (LQTS3). For example, the rate-dependent effects of the ΔKPQ mutation in *SCN5A* on AP properties were investigated with a Markov model of the cardiac Na^+^ channel with parameters optimized to reflect the behavior of WT or ΔKPQ Na^+^ channels (152, 198). Incomplete penetrance of these mutations was further investigated through the development of populations of models (199). In particular, the same mutation-induced electrophysiological changes in *I*_Na_ were applied to 1,000 model instances with different baseline electrophysiological profiles (but all producing APs within the experimental range) for three different LQTS3 mutations. Of the 1,000 models, 580 showed no phenotype (defined as EAD formation or excessive APD prolongation) with any of the mutations, 135 showed a LQTS phenotype with all three mutations, whereas the remaining models were susceptible to one or two mutations. Models with an appropriate balance of repolarizing currents, typically via increased *I*_Ks_ or *I*_K1_, were less susceptible, but the most critical repolarizing current was mutation specific.

Other studies have modeled mutations in potassium channels such as *KCNQ1* (underlying *I*_Ks_) and *KCNH2* (underlying *I*_Kr_), providing mechanistic understanding of the occurrence of arrhythmic events in LQTS1 (200) and LQTS2 (201, 202), short QT syndrome (168, 203), or familial AF (204, 205). Similarly, models have described more indirect alterations (not studied through patch-clamp experiments in heterologous systems) such as the biophysical effects of mutations in cardiac RyR2 channels and CSQ2, providing insight into the subcellular mechanisms of diseases like catecholaminergic polymorphic ventricular tachycardia (CPVT) (206). In addition to single channelopathies, mutations may affect multiple channels, and computer models can elucidate the combined effects. For example, Tyan et al. (207) showed that mutations in the CAV3 gene, encoding the ubiquitous scaffolding protein caveolin-3, produce mutation-specific changes in multiple ion currents, which lead to different primary causes of APD prolongation in the O’Hara–Rudy (ORd) model. Similarly, computer models can help integrate the effects of compound mutations in different ion channels. For example, Hu et al. (208) showed that a loss of function in *I*_Na_ coupled with a gain of function in *I*_CaL_, caused by mutations in *SCN5A* and *CACNB2b*, may underlie the development of cardiac conduction disease without Brugada syndrome.

Cell-level computer models are also highly suitable to investigate how mutations may interact with external factors such as electrolyte imbalances (150, 209), sympathetic stimulation [a proarrhythmic factor in patients with LQTS1, LQTS5, and CPVT (38, 210, 211) but a protective factor in LQTS3 and BrS (212)], or pharmacological treatments (discussed below). Recent advances in hIPSC-CM have allowed the study of genetic variants in a patient-specific context, partially reducing the need for computational models for extrapolating the biophysical effects of genetic mutations. However, computer models provide a more mature electrophysiological phenotype and enable a more comprehensive characterization of the role of external factors, as well as providing the opportunity to assess the consequences for arrhythmogenesis at the organ level (discussed in sect. 3). In addition, computer models may help interpret results from hIPSC-CMs. For example, computer models of hIPSC-CM cells have been used to evaluate proarrhythmic risk in phenotypically variable populations harboring mutations in *KCNQ1* (213).

Developments in artificial intelligence (AI) and machine learning can help to predict the proarrhythmic effects of ion channel mutations, but models focusing on associations between a genetic variant and clinical phenotype are hindered by incomplete penetrance and overlap syndromes, calling for an integration of functional in vivo studies, computational modeling mechanistic studies, and machine learning techniques. However, at present, even the prediction of the biophysical effect of a genetic variant at the level of the ion channel is challenging because of reliance on sparse datasets obtained under heterogeneous conditions that are biased toward disease-causing mutations (214). The development of novel machine learning tools to translate amino acid sequences to 3-D protein structure (84) and the ability to simulate channel gating over physiologically relevant timescales based on such structures (75, 87) (see sect. 2.2.1) is expected to help bridge the current genotype-phenotype knowledge gap, potentially opening up opportunities for personalized antiarrhythmic drug treatment.

**Table 2.** Overview of modifications to the main human ventricular cardiomyocyte models

Model	Innovation from Original Model and Purpose
*Original model: O’Hara–Rudy 2011 (67)*	
Bartolucci 2020 (150)	Capture the inverse dependence of APD on extracellular Ca^2+^ and APD rate dependence at 4 mM extracellular K^+^Changes to: • sensitivity of Ca^2+^-dependent inactivation of *I*_CaL_ • reparameterization of calcium handling parameters
Gando 2020 (151)	Replacement of fast and late Na^+^ current by Markov model (152)
Passini 2016 (153)	Improve reproduction of experimental data through: • I_to_ increase • extracellular concentrations set as experiments • modified K^+^ equilibrium potential • modified *I*_Na_ steady state inactivation • modified current stimulus
Tomek 2020 (73)	• Improve behavior of O’Hara–Rudy 2011 (67) for AP plateau, APD accommodation in response to heart rate acceleration, *I*_Na_ block. Main changes: reevaluation of *I*_CaL_ and *I*_Kr_ • Explicit validation of drug effects with an independent experimental dataset
Lee 2017 (154)	• Addition of the Heijman (41) beta-adrenergic stimulation model • *I*_Ks_ formulation modified to account for Ca^2+^ dependence of *I*_Ks_ (Markov *I*_Ks_)
Whittaker 2017 (155)	• Modified *I*_Na_ formulation [from Luo–Rudy (34)] • Markov formulation of *I*_Kr_
Li 2017 (CiPA) (156)	Dynamic hERG drug-binding model for *I*_Kr_
Dutta 2017 (157) (CiPA)	Scaling of *I*_Kr_, *I*_Ks_, *I*_K1_, *I*_CaL_, *I*_NaL_ to fit published APD rate dependence experimental data and response to drug block [based on Li 2017 (156)]
Romero 2015 (158)	Markov formulation of *I*_Kr_ based on Fink 2008 (159)
Trenor 2013 (160)	New formulation of *I*_NaL_ (conductance fitted to experimental data)
*Original model: Grandi (147)*	
Carro 2011 (161)	• Reformulating of *I*_CaL_ [fast and slow inactivation gate as in ten Tusscher (144)] and *I*_K1_ • Redefinition of *I*_Na_ and *I*_NaK_ to improve APD restitution curve shape, slope, and APD response to heart rate changes
Trenor 2012 (162)	New formulation of *I*_NaL_ [based on ten Tusscher (144)], in the setting of heart failure
Asakura 2014 (163)	Inclusion of calcium-induced calcium release as in Hinch model of CaRU: local control of RyR by L-type calcium channel
*Original model: ten Tusscher (144)*	
Xia 2006 (164)	• Inclusion of *I*_NaL_, *v*_max_ • Modification of *I*_NaCa_, *I*_to_, *I*_Ks_ based on recent human experimental data
ten Tusscher 2006 (165)	Reduced model to improve computational efficiency [like Bernus (166)]
Grandi 2009 (167)	New formulations of the Ca^2+^ dependence of *I*_Ks_, *I*_Kr_, and *I*_CaL_ to capture APD dependence on extracellular calcium
Fink 2008 (159)	Updated K^+^ currents: *I*_K1_ (accounting for the blocking effects of intracellular magnesium and spermidine on this potassium conductance), *I*_Kr_ (Markov model)
Adeniran 2017 (168)	Markov model for *I*_Ks_
*Original model: Bueno-Orovio (77)*	
Kienast 2017 (169)	Effect of temperature (cooling-induced effect on AP)
Bueno Orovio 2012 (170)	Capture APD adaptation dynamics (by adaptation of model parameters)
*Original model: Priebe and Beuckelmann (149)*	
Bernus 2002 (166)	Reformulation to be computationally efficient (2-D), 6-variable model

AP, action potential; APD, AP duration; CiPA, Comprehensive in vitro Proarrhythmia Assay; 2-D, 2-dimensional; CaRU, Ca^2+^ release unit; *I*_Ca,L_, L-type Ca^2+^ current; *I*_K1_, basal inward-rectifier K^+^ current; *I*_Kr_, rapid delayed-rectifier K^+^ current; *I*_Ks_, slow delayed-rectifier K^+^ current; *I*_Na_, Na^+^ current; *I*_Na,L_, L-type Na^+^ current; *I*_to_, transient outward K^+^ current; RyR, ryanodine receptor.

#### 2.4.3. Antiarrhythmic drug treatment.

Despite significant advances and a growing importance of catheter ablation for the treatment of cardiac arrhythmias (discussed in sect. 3), antiarrhythmic drugs (AADs) remain a key component of antiarrhythmic therapy, particularly in patients with AF (215). However, currently available AADs have imperfect efficacy and are associated with proarrhythmic side effects (215, 216). These issues are in part due to the pleiotropic effects of most AADs, which differ between cardiac regions (atrial vs. ventricular vs. nodal cells) and depend on disease-related electrophysiological remodeling, making the prediction of the combined electrophysiological effects and their pro- or antiarrhythmic consequences challenging. There remains a need for novel safer, more effective AADs. Cardiac cellular electrophysiology models have emerged as a useful tool for investigating the integrative effects of AADs and characterizing the potential antiarrhythmic effects of novel compounds on the basis of their experimentally observed affinities for different ionic targets.

Traditionally, the inhibitory effect of AADs on ion channels was simulated as a reduction in maximal conductance, typically based on a sigmoidal Hill function of the drug concentration (217–219). This approach can provide relevant insights into the combined electrophysiological effects of targeting multiple ion channels, e.g., revealing the synergistic antiarrhythmic effect of targeting multiple K^+^ channels at fastV versus slow pacing rates using experimentally calibrated populations of virtual human atrial myocyte models (220). Moreover, by evaluating the electrophysiological effects of a compound in several models representing different species with and without disease-related remodeling, computational models can provide insight into species (219)- and disease (221)-related differences in antiarrhythmic effects that hinder the translation to clinical application (222). Moreover, the limited computational complexity of implementing AAD effects in this manner enables multiscale investigations of their antiarrhythmic effects for acute cardioversion and long-term sinus rhythm maintenance (223, 224).

However, many AADs have state-specific affinities for an ion channel, e.g., only binding to the channel in the inactivated state. These state-dependent interactions are particularly pronounced for Na^+^ channel blockers. Markov models allow incorporation of state-specific drug effects by adding drug-bound states only to specific parts of the model. These models have been used to study the interaction between channel gating and drug effects of different subtypes of Na^+^ channel blockers in the absence or presence of mutation-induced changes to channel gating (151). For example, Clancy et al. (225) showed that mexiletine (which primarily blocks the channel in the open state) and lidocaine (which primarily acts on the inactivated state) differentially affect AP upstroke and the occurrence of afterdepolarizations. Similarly, Markov model-based computational modeling approaches have been used to assess the balance between Na^+^ channel and *I*_Kr_ inhibition by ranolazine (226) and to optimize the AF selectivity of *I*_Kur_ inhibition (128). So far, very few studies have incorporated these detailed models of drug-channel interaction into multicellular organ-level models to assess the potential clinical effectiveness of different types of AAD treatment.

Dynamic clamp makes it possible to subsequently study the electrophysiological effects of selective alterations in channel gating (which might not yet be achievable with currently available AADs) in the native environment of the cardiomyocyte. For example, dynamic clamp studies suggested that altering the gating of the L-type Ca^2+^ channel to reduce its sustained component would reduce the occurrence of EADs (227, 228). This result was subsequently confirmed by a fully experimental approach with a novel pharmacological compound (229).

#### 2.4.4. Cardiac safety pharmacology.

Drug-induced proarrhythmia is a major adverse effect limiting the clinical application of currently available AADs (215, 216). More in general, presumed cardiac safety liabilities are a major driver of attrition in drug development (230). Traditional cardiac safety pharmacology featured a prominent role for in vitro assays characterizing the affinity of a compound to block Kv11.1 (also known as hERG) underlying *I*_Kr_ (231, 232). This approach was based on the prominent role of *I*_Kr_ in repolarization of the human ventricular cardiomyocyte and the association between excessive repolarization prolongation and the genesis of ventricular tachyarrhythmias (notably torsades des pointes, TdP). However, the association between *I*_Kr_ inhibition and drug-induced proarrythmia has modest specificity, potentially leading to drug candidates that otherwise have desirable properties being incorrectly discarded (232). This motivated the “Comprehensive in vitro Proarrhythmia Assay” (CiPA) initiative developed in collaboration by academic scientists, the pharmaceutical industry, and the US Food and Drug Administration (233). The CiPA initiative is, among other things, based on a more extensive characterization of the effects of a drug candidate on multiple ion channels and the subsequent integration of this information with computational models.

**FIGURE 8.**
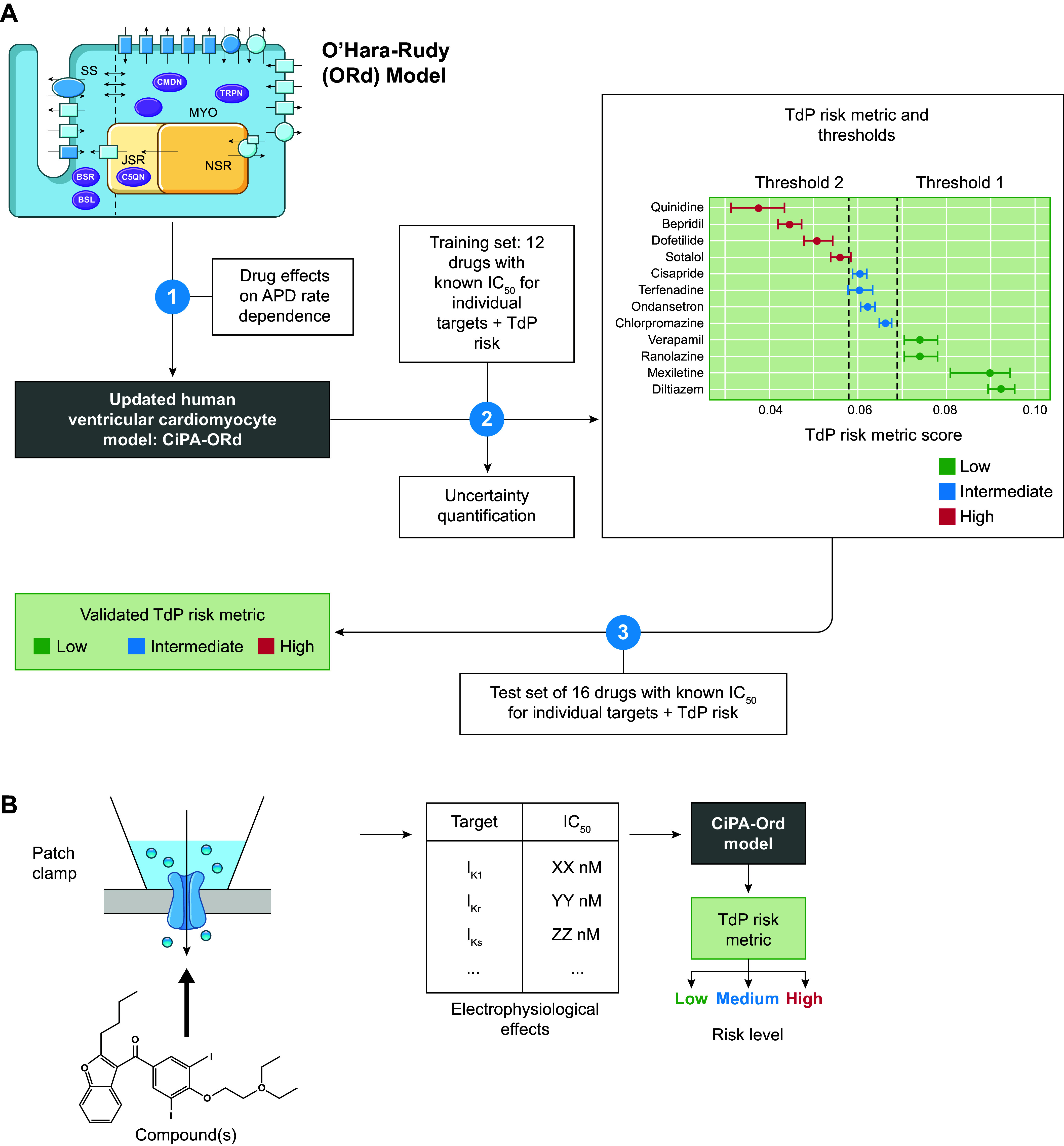
Use of cardiac cellular electrophysiology models in cardiac safety pharmacology: the Comprehensive in vitro Proarrhythmia Assay (CiPA) initiative. *A*: an updated version of the O’Hara et al. (67) model of the human ventricular cardiomyocyte by Dutta et al. (157) (CiPA-ORd) was used to simulate the effects of a compound on individual ion channels (*step 1*). A torsade des pointes (TdP) risk metric score was calculated, and thresholds for separating low-, medium-, and high-risk drugs were established based on a training set of 12 drugs with known half-maximal inhibitory concentrations (IC_50_ values) for different ion channels and known clinical TdP risk (*step 2*). The model performance was validated in an independent set of 16 drugs (*step 3*). *B*: the model can be employed to assess the risk of new compounds based on their experimentally characterized effects on different ion channels. APD, action potential duration; *I*_K1_, basal inward-rectifier K^+^ current; *I*_Kr_, rapid delayed-rectifier K^+^ current; *I*_Ks_, slow delayed-rectifier K^+^ current.

The ORd model of the human ventricular cardiomyocyte (67) was chosen as the consensus base model for the in silico prediction of drug effects, which were simulated by scaling the maximal conductances of individual ion channels. This model was subsequently updated by Dutta et al. (157) to more closely reproduce experimentally observed drug-block effects and capture dynamic *I*_Kr_ drug-binding kinetics (FIGURE 8*A*, *step 1*). The updated model was used to calculate a qNet score that could separate 12 predefined CiPA training drugs into their three clinical risk categories (low, medium, high risk of drug-induced TdP) (157) (FIGURE 8*A*, *step 2*). Given the potential important implications of these model-based decisions, uncertainty quantification was performed on this population with the nonparametric bootstrap method and a Bayesian inference approach propagated through AP simulations (234). This was followed by a detailed prespecified validation procedure on an independent set of 16 drugs with known TdP risk (FIGURE 8*A*, *step 3*). Model-based risk prediction showed a median area under the receiver operating characteristics curves close to 0.90 (235). Other studies similarly showed that human in silico drug trials exhibit higher accuracy than animal models in predicting clinical drug-induced proarrhythmia (236, 237). Recently, this approach has also been applied to combinations of drugs, revealing highly nonlinear effects on qNet score (and thus predicted risk of TdP) of different drug combinations (238). Together, these studies show that cardiac cellular electrophysiology models can accurately assess the risk of drug-induced TdP and can support real-world decisions with significant financial and safety implications (FIGURE 8*B*). Future work may integrate these models with molecular models of cardiac ion channels to predict binding sites and affinities to generate an integrated in silico cardiotoxicity prediction pipeline, as has recently already been done for the inhibition of Kv11.1 by dofetilide and moxifloxacin (239).

## 3. ORGAN-LEVEL COMPUTATIONAL MODELING OF CARDIAC ELECTROPHYSIOLOGY: UNDERSTANDING HUMAN DISEASE AND PAVING THE ROAD TO CLINICAL APPLICATIONS

### 3.1. Construction of Whole Heart Computational Models

#### 3.1.1. Model geometry, fiber orientations, and computational meshes.

Whole heart models integrate information from multiple scales ranging from cell-level ionic properties to whole organ disease remodeling. Here we review the fundamental concepts of constructing whole heart models, including methods of model personalization using clinical data. Typically, models are either ventricular (240) or atrial (241) rather than four-chamber models, with a few exceptions (242, 243). The reason for the separate applications of atrial and ventricular models in cardiac electrophysiology lies in the somewhat different types of arrhythmias in the lower and upper chambers in the heart, resulting in different clinical approaches to arrhythmia management in patients (244–246).

The majority of published atrial and ventricular models, at least since the year 2000, are individualized, at least in terms of geometry and anatomical structure, thus enabling the examination of differences in arrhythmogenic behavior between different patients or individual animal models (the latter used in the exploration of heart disease and how it imparts arrhythmogenicity). Information about geometry and anatomical structure of the heart has been predominantly acquired by nondestructive means (i.e., medical imaging), although examples of geometry reconstruction executed painstakingly from histological sectioning are available (247). Two major imaging modalities that can be used for model personalization are cardiovascular magnetic resonance imaging (MRI) and computed tomography (CT).

**FIGURE 9.**
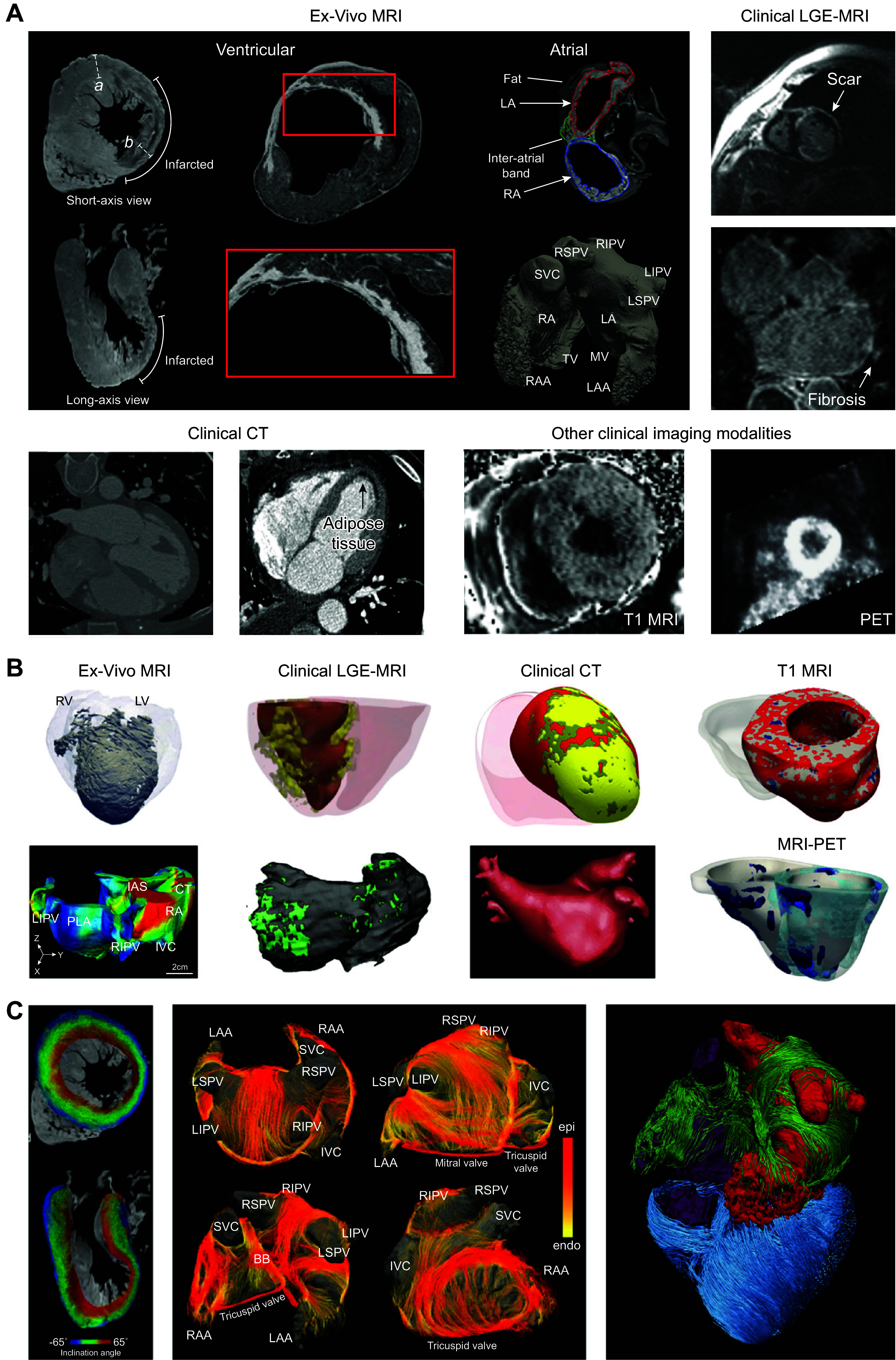
Computational models of the heart. *A*: imaging modalities used in whole heart modeling. Ex vivo images reproduced from Ref. 254, with permission from *Journal of Cardiovascular Magnetic Resonance* (*left*), and Ref. 256 (*center*) and Ref. 255 (*right*), with permission from *Circulation: Arrhythmia and Electrophysiology*. *B*: personalized atrial and ventricular models reconstructed from various imaging modalities. Images of ex vivo ventricular and atrial models reproduced from Ref. 256 (*top*), with permission from *Circulation: Arrhythmia and Electrophysiology*, and Ref. 257 (*bottom*), with permission from *Journal of American Heart Association*. Images of late gadolinium enhancement (LGE)-magnetic resonance imaging (MRI)-based ventricular and atrial models reproduced from Ref. 258 (*top*) and Ref. 259 (*bottom*), with permission from *Nature Biomedical Engineering*. Computed tomography (CT) images reproduced from Ref. 260 (*top*), with permission from *Circulation: Arrhythmia and Electrophysiology*, and Ref. 261 (*bottom*), with permission from *Progress in Biophysics and Molecular Biology*. Image of T1-MRI-based model reproduced from Ref. 262, with permission from *eLife*. Image of MRI-positron emission tomography (PET) model reproduced from Ref. 263, with permission from *Science Advances*. *C*: fiber orientations mapped to personalized heart models. Images reproduced from Ref. 254 (*left*), with permission from *Journal of Cardiovascular Magnetic Resonance*, and Ref. 255 (*center*), with permission from *Circulation: Arrhythmia and Electrophysiology*. BB, Bachmann’s bundle; CT, crista terminalis; IAS, interatrial septum; IVC, inferior vena cava; LA, left atrium; LAA, left atrial appendage; LIPV, left inferior pulmonary vein; LSPV, left superior pulmonary vein; LV, left ventricle; PLA, posterior left atrium; RA, right atrium; RAA, right atrial appendage; RIPV, right inferior pulmonary vein; RSPV, right superior pulmonary vein; RV, right ventricle; SVC, superior vena cava.

In addition to providing information about heart shape, MRI imparts excellent heart tissue characterization, principally through the use of gadolinium-based contrast agents, which accumulate in scar and fibrotic tissue (248). Areas on MRI with late gadolinium enhancement (LGE) correspond to areas of scar and fibrotic remodeling, with the increase in image intensity typically signifying a transition to deep scar (249). Both ex vivo MRI and clinical MRI scans have been used in heart model construction; the ex vivo MRI models were of explanted animal or human hearts and used to reconstruct both heart geometries and tissue scarring (250–253). The highest MRI resolution scans of a large heart (with a clinical bore MRI) for the purpose of characterizing whole heart structure and whole heart model construction were published as a series of submillimeter ex vivo MRI studies (8, 254–256) of human atria and ventricles, as well as animal (swine) hearts with myocardial infarction (scarring). Examples of the images used in modeling studies are shown in FIGURE 9*A, left*; acquisition of the images characterizing the infarct was done with LGE. Such images have allowed for the development of high-resolution computational models of ex vivo ventricles and atria, examples of which are shown in FIGURE 9, *A* AND *B*, *left*.

In contrast, the resolution of in vivo clinical LGE-MRI is a challenge for computational model generation; the current clinical standard LGE-MRI is a 2-D sequence with spatial resolution of 1- to 2-mm in-plane resolution and 6- to 8-mm slice thickness (FIGURE 9*A, top right*). Nonetheless, several impactful translational atrial and ventricular computational modeling approaches have been developed (258,259, 264) utilizing the clinical scans, as reviewed below in this article. Recent developments in 3-D imaging allow for the acquisition of high-resolution LGE images with reduced partial volume averaging (1- to 2-mm in-plane resolution and 2- to 4-mm slice thickness), potentially providing better information regarding the spatial organization of disease-induced tissue remodeling. Examples of atrial and ventricular models reconstructed from higher-resolution (3-D) clinical scans that incorporate scar/fibrosis distributions are shown in FIGURE 9*B,* second from *left*. An additional challenge in using clinical MRI, specifically for ventricular model generation, is that patients with a history of ventricular tachyarrhythmia (VT) typically have implantable cardioverter-defibrillators (ICDs), which pose major challenges to acquiring ventricular geometry and substrate characterization, as the physical proximity of the ICD generator to the heart can result in image artifacts that obscure visualization of the myocardium (265). These artifacts can be reduced (though not completely eliminated) by using a modified wideband pulse sequence (266). Ventricular modeling studies have utilized both MRI scans with artifact and the wideband sequence scans in model generation with limited success (258).

Cardiac CT has some distinct advantages over MRI for heart model generation. Most importantly, cardiac CT has superior spatial resolution (0.5- to 2-mm^3^ isotropic resolution) compared with MRI (FIGURE 9*A*, *bottom left*). In addition, the artifact from the ICD device is minimal. CT has been widely used in the reconstruction of atrial geometrical models (55, 261, 267); see an example of such atrial model reconstruction in FIGURE 9*B,* second from *right*. The main issue with the utilization of CT scans in image-based personalization of ventricular models is the lack of contrast agent that can visualize scar and fibrosis in the heart. Although delayed-enhancement CT with iodinated contrast has been attempted, the relative gray tone differences in tissue radiopacity between scar and normal tissue are small compared with LGE-MRI (268). Consequently, the presence of myocardial fibrosis has been inferred by using surrogate measures such as regional left ventricular (LV) wall thinning (269, 270) and used in ventricular computational modeling (271). Recently, penetrating adipose tissue has been recognized as a potential substrate for ventricular arrhythmias in addition to scar/fibrosis (272). A recent study created and utilized the first ventricular model with infiltrating adiposity (FIGURE 9*B,* second from *right*), using contrast-enhanced CT scans with hypoattenuation (FIGURE 9*A, bottom left*) visualizing adipose tissue distribution (260); subsequently, hybrid LGE-CT ventricular models were developed in a prospective patient study (273), exploring the combined contribution of scar and penetrating adiposity to ventricular arrhythmogenesis in ischemic cardiomyopathy.

Finally, other imaging modalities have also been used in heart model construction. Postcontrast T1 mapping, a parametric MRI modality, visualizes diffuse fibrosis (274, 275) (FIGURE 9*A*, *bottom right*). As T1 maps are typically acquired for a single slide through the ventricles, they have been used together with LGE-MRI scans to provide imaging input into ventricular models representing both scar and diffuse fibrosis, such as those of hypertrophic cardiomyopathy (262) (FIGURE 9*B*, *right*). Finally, a combination of LGE-MRI and positron emission tomography (PET) scans, the latter visualizing the distribution of inflammation in the heart (276) (FIGURE 9*A*, *bottom right*), was used to create fusion ventricular models (FIGURE 9*B*, *right*) of patients with cardiac sarcoidosis (263), an inflammatory heart disease.

In addition to atrial or ventricular geometry and heart disease pathology implicated in arrhythmias (scar, fibrosis, penetrating adipose tissue, inflammation), the 3-D fiber orientation in the myocardium is an important component in anisotropic propagation (277). Thus, both atrial and ventricular models incorporate fiber orientations, ensuring realistic conduction patterns. At the organ level, fiber orientations can be acquired in a nondestructive manner by using diffusion-tensor (DT) MRI, which visualizes the fiber tracts in the myocardium (250, 251, 254, 255). DT-MRI requires very long scan times to achieve resolution informative about fiber orientations; thus it has been performed in ex vivo hearts (278, 279) and used for organ-level modeling studies of electrophysiology and arrhythmias (280).

Recent studies have successfully acquired fiber orientations in explanted large animal and human hearts on a clinical scanner (254–256), thus providing valuable information about human atrial and ventricular structural organization, including fiber orientations in the zone of infarct (FIGURE 9*C*). Acquisition of fiber orientation has also been attempted in patients (281); however, the resolution is very low and not applicable for modeling studies. Human heart modeling studies have used the acquired fiber orientation information in different ways. In ventricular applications, rule-based approaches (282, 283) have been developed that allow, given a ventricular geometry, assignment of fiber orientations specific to the geometry that follow rules extracted from the available fiber imaging information (the atlas). Briefly, the Laplace–Dirichlet method was used to define transmural and apicobasal directions at every point in the ventricles, and then bidirectional spherical linear interpolation was applied to assign fiber orientations based on a set of rules. These rule-based approaches are now the state-of-the art methodology in assigning fiber orientations in clinical translational projects with personalized simulations of patient ventricular heart electrophysiology (53, 258, 284–294). Atrial fiber orientations are significantly more complex and not readily amenable to developing automatic rule-based approaches. Atrial patient-specific models have used either a manual reconstruction of fiber orientations based on general principles (295, 296) or, recently, the explanted atrial fiber orientations shown in FIGURE 9*C* (297).

Reconstruction of atrial and ventricular model geometry requires segmenting the chambers of the heart and the remodeled tissue (scar, fat, inflammation, and potentially the respective border zones). For personalized 3-D ventricular model generation, the ventricular myocardium is typically segmented semimanually from LGE-MRI or CT and reconstructed with variational implicit function interpolation (258, 260, 263, 264, 285, 286, 291, 298, 299), although recent studies have proposed fully automatic deep-learning approaches to ventricular segmentation (300). Recent studies have also used PET in personalized ventricular modeling (263); however, segmentation of the ventricular myocardium directly from PET is not feasible because of low image resolution. PET segmentation can instead be achieved by registration of PET images with segmentations from a higher-resolution imaging modality (e.g., LGE-MRI), and regions of active inflammation can be identified from PET by thresholding of the body weight-standardized uptake value (263).

Multiple semiautomatic methods have been proposed for fibrosis and border zone identification from LGE-MRI (301, 302). One such method is the signal threshold to reference mean method, in which the myocardium is binarized into regions of high and low intensity and the mean of the lower-intensity region is selected as the reference mean intensity of normal myocardium. Regions of border zone and scar are then identified by signal thresholding with respect to the reference mean, but the optimal threshold is controversial (302–304), and reproducibility is lower for this method compared with others (301). Another proposed method, full width of half-maximum (FWHM), has been shown to have higher reproducibility (301) and has been used in recent simulation studies (258). In FWHM, the myocardium is similarly binarized into low- and high-intensity regions and then the high-intensity region is further divided into border zone and scar by defining scar as signal exceeding 50% of the maximum intensity (305).

Another remodeled tissue type, LV infiltrating adipose tissue (inFAT), represented in personalized ventricular modeling studies (260, 273) was identified as contrast-enhanced computed tomography (CE-CT) signal intensity in the range −180 to −50 HU (306–308). Previous studies have included hypoattenuated voxels with intensities greater than −50 HU as part of adipose tissue (307, 309), but these likely represent an admixture of adipose and myocardial tissues (310). Thus, tissues with intensity from −50 to −5 HU were distinguished separately from inFAT and termed fat-myocardium admixture, analogous to gray zone identified on LGE-MRI (260).

Segmentation of clinical images for ventricular model construction (such as heart geometry and scar distribution from LGE-MRI) is a laborious manual task, and, naturally, AI approaches have been developed and deployed to accelerate this process and potentially remove the intraobserver bias associated with manual image segmentation. The first attempts in this direction were not robust to varying image acquisition quality (311, 312). Popescu et al. developed an anatomically informed deep-learning approach to LGE-MRI ventricular image segmentation and clinical feature extraction (300). This fully automated technology applies a cascade of deep neural networks to ensure anatomical accuracy of segmentations that is robust to different scar/fibrosis distributions. A similar approach was subsequently applied to segmentation from CT scans, including the distribution of inFAT.

Atrial segmentation is a much harder task, as the walls of the atria are much thinner and the images are harder to acquire (313). A number of studies have instead chosen to use a surface model of the atria (314–317). Delineation of atrial fibrosis is a particularly onerous task, with scar from atrial ablation lesions better visible on the atrial LGE-MRI than native fibrosis (318, 319). The threshold for fibrosis segmentation is controversial; thus multiple methods have been proposed for identifying and quantifying the patient-specific fibrosis distributions in patients with AF (259, 313, 320–323). The Utah scoring method (313, 321, 322), applied only to the left atrium (LA), uses the distribution of the myocardial intensities to define the normal. The CEMRG tool (320) uses a maximum-intensity projection method where myocardial voxels are identified as fibrotic if the intensities are 3 standard deviations above mean blood pool (BPM) intensity. The image intensity ratio (IIR) identifies LA fibrosis with myocardial intensities that are >1.22 times the BPM intensity (323). The IIR approach has been specifically designed to mitigate the uncertainty in threshold-based methods in segmenting LGE-MRI scans (324), since it uses ratiometric values instead of raw voxel intensities (323). As most of the modeling studies have been on the LA, only a few studies have attempted to identify fibrosis in the right atrium (RA). Boyle et al. (259) adapted the IIR metric for RA fibrosis while using the patient-specific LA BPM as the reference. AI approaches to segmentation of atrial geometry and fibrosis distribution have also been developed (325, 326).

For both animal and human ventricular and atrial models, finite-element ventricular meshes are generated from the segmented images (327, 328); the equations for electrical wave propagation are solved on these meshes. An average resolution of these meshes is 350 μm; this choice of finite element size is dictated by the need to resolve wavefront propagation in the simulations while simultaneously minimizing computational expense (329). Fiber orientations in the mesh are assigned on a per-element basis. In meshes where fiber orientations are assigned from an atlas (i.e., not using a rule-based method), this is typically achieved by using either diffeomorphic mapping techniques (330, 331) or the universal coordinate system approach (297, 332, 333) to transform the relevant conductivity tensors from the geometry of an atlas mesh into the patient-specific geometry. After fiber orientation is assigned to the elements in the ventricular mesh, the corresponding “masks” of areas with structural remodeling are superimposed. Finally, atrial and ventricular LGE-MRI scans have also been used to assess conduction velocity in the heart, to be used in computational modeling (334, 335).

#### 3.1.2. Governing equations for electrical activity in the myocardium.

The bidomain equations represent the mathematical description of electrical wave propagation through cardiac tissues composed of both intracellular and extracellular spaces in which electrical current flows (336). The intracellular (φ_i_) and extracellular potentials (φ_e_) in these spaces are coupled through the transmembrane current density *I*_m_, which represents the ionic exchange through ion channels, pumps, and other transporters. The mathematical equations consist of a system of partial differential equations with respect to space and time (*Eqs. 1–4*):

(*1*) $$\nabla \times \sigma_{i}\nabla \varphi_{i}=\beta I_{m}$$

(*2*) $$\nabla \times \sigma_{\text{e}}\nabla \varphi_{e}=-\beta I_{m}$$

(*3*) $$I_{m}=C_{m}\frac{\partial V_{m}}{\partial t}+I_{\mathrm{ion}}\left(V_{m},\eta \right)-I_{s}$$

In the equations, *V*_m_ represents the transmembrane voltage and the difference between φ_i_ and φ_e_. σ_i_ and σ_e_ are the intracellular and extracellular conductivity tensors, respectively. β is the ratio between the membrane surface area and the volume. *I*_s_ represents an external stimulus applied across the membrane. *C*_m_ is the membrane capacitance per unit area, *I*_ion_ is the transmembrane ionic current density, and η represents the gating variables that govern the kinetics of the different ionic currents.

The monodomain equations are a simplification of the bidomain equations and are derived by assuming either a proportionality between σ_i_ and σ_e_ or that the extracellular space is infinitely large (337). With this assumption, the bidomain system above (*Eqs. 1–3)* is simplified into a single partial differential equation, a reaction-diffusion equation (*Eq. 4*):

(*4*) $$\nabla \times \sigma_{m}\nabla V_{m}=C_{m}\frac{\partial V_{m}}{\partial t}+I_{\mathrm{ion}}\left(V_{m},\eta \right)-I_{s}$$

In the above formulation, σ_m_ is the effective monodomain conductivity that relates σ_i_ and σ_e_. In electrophysiological simulations, monodomain equations are often used in place of the bidomain system because of the enormous savings in computational costs (338).

#### 3.1.3. Representation of electrophysiological properties in whole heart models.

The ionic current *I*_ion_ in the above equations encompasses the membrane dynamics and is the sum of all the currents through the membrane and subcellular compartments during the generation of the cardiac AP. In cardiac computational modeling, electrophysiological properties of individual cardiac cells, whether in health or disease, are represented by the components of the ionic current, and the first part of this article reviewed all models of human cardiac electrophysiology that have been developed and utilized in cardiac computational research with a clinical translation focus. In organ-level modeling, each element of the 3-D computational mesh is assigned ionic properties, i.e., an AP model. An organ-level computational mesh could have all of its elements defined with the same action potential model or could have regional differences in the AP models throughout the 3-D geometry. These regional differences could represent differences in chamber electrophysiological properties, apicobasal and transmural gradients in properties, or regional heterogeneities due to disease remodeling. In such cases, parameters in the chosen baseline (human) AP model are modified on a regional basis.

In this part of the review, we focus on whole heart models with (potential) clinical significance; to execute these in a reasonable time frame and with reasonable computational resources, only a portion of the human ionic models described in the first part of the review have been utilized in whole heart models. The typical goal has been to execute a simulation of electrophysiological activity in the whole heart over a few minutes in a few hours on a high-performance computational resource. Having such a simulation run for days (or weeks in some cases) makes it difficult to obtain meaningful results in any clinical workflow time frame or to conduct a research study that involves a number of personalized heart models rather than a single one or two. Clearly, individual research teams have made choices regarding the trade-off between fidelity of electrophysiological phenomena at the cell level in the whole heart models and speed of execution of the simulations, depending on the specific goals of the research projects. Overall, simulation studies that have focused on the nonlinear dynamics of the animal or human heart predisposing to arrhythmogenesis (339–343) have utilized simpler formulations, as these nonlinear phenomena, such as alternans, are mostly dependent on the interaction of activation and repolarization in the context of cardiac restitution properties (344). Similarly, studies exploring arrhythmogenesis of the structurally remodeled substrate in a patient heart, reviewed below, have also utilized simpler sets of membrane kinetics equations because these typically require simulations of pacing-induced activity from many sites and over longer periods of time (minutes in real time). In these studies, structural remodeling such as fibrosis or penetrating adipose tissue has been incorporated via regional alterations of electrophysiological properties. Areas of fibrosis have been represented in atrial models as electrical conduction disturbances (lower conductivity, edge splitting, or percolation), remodeling of ion channels (e.g., induced by transforming growth factor-β1), electrical myocyte-fibroblast coupling, discrete microstructural alternations in gap junction connectivity, and combinations of these (345–349). Selection of fibrosis modeling methodology is critical, as the specific representation of fibrosis has a significant effect on rotor dynamics (350, 351). Quantification of the uncertainty related to imaging, fibrosis detection, and fibrosis representation and incorporation of this uncertainty in model predictions is an important current avenue of research. Finally, a number of organ-level computational studies have opted to incorporate more complex cell models in order to explore how (drug induced) ion channel kinetics or subcellular phenomena (e.g., spontaneous Ca^2+^ releases) are manifested at the level of the whole heart (95, 352–355).

#### 3.1.4. Personalization of electrophysiological properties.

In computational modeling of arrhythmias at the organ level, personalization is typically done through the creation of individualized geometry as described above. In addition to assigning distinct electrophysiological properties in models in different regions based on remodeling obtained from the images, studies have also used electrical or optical measurements that can be obtained experimentally in ex vivo studies for model personalization (356, 357). Clinical measurements have provided noninvasively or invasively acquired data to personalize heart models. In clinical applications, model electrophysiological parameters have been calibrated with intracardiac electrograms measured during ablation procedures to better reflect patient-specific electrophysiology (358–362). Electrograms are measured locally at locations throughout the endocardial or epicardial surface. This information has typically been used to estimate conduction velocity and regional APD, which have subsequently been used for model calibration (363, 364). Additionally, in clinical applications studies have used either the 12-lead ECGs or electrocardiographic imaging (ECGI; Refs. 365, 366) data to calibrate model electrophysiological parameters from noninvasive measurements (290, 367–372). However, it remains nontrivial to determine which model parameters need to be changed to reflect the optical or electrical measurements, as multiple combinations of ion channel parameters can give the same APD (373).

Advances in machine learning could allow the use of currently available noninvasive measurements of cardiac electrical activity such as QRS duration extracted from noninvasive 12-lead ECG to adjust conduction velocity in patient-specific models (374, 375). Zettinig et al. (2014) (376) used polynomial regression to calibrate three diffusivity parameters of a phenomenological model by using the QRS duration and electrical axis features. A complete personalization of a biophysically detailed model would require more computational resources, as a large quantity of forward model simulations would be required to train most machine learning algorithms.

#### 3.1.5. Simulating electrical activity of the heart at the organ level.

In the heart, myocytes are electrically connected via low-resistance gap junctions. Current can flow from cell to cell via this pathway, in addition to the current exchange between intracellular and extracellular spaces through cell membrane proteins, as described above. Propagation in the heart is typically modeled with spatially continuous models that are viewed as resulting from a local spatial homogenization of behavior in tissue compartments (membrane, intracellular, and extracellular spaces). Simultaneous solution of the monodomain reaction-diffusion partial differential equation over the organ volume, accounting for the conductivity tensor fields, together with the set of ionic model equations at the nodes of each finite element (whether the same throughout or regionally different ionic model) represents simulation of electric wave propagation in the heart. The solution of the organ-level monodomain equation together with the ionic model equations defined at each node of the organ mesh is a representation of the multiscale simulation approach in cardiac electrophysiology. In a multiscale simulation, one could observe behavior at the organ level (such as wavefront propagation) but also could “dive down” at a specific location and examine, for instance, what the Ca^2+^ transient is at this location within the organ. For detailed information on the various numerical approaches used in solving this system of equations, we direct the reader to comprehensive reviews on the subject (329, 377, 378). There are currently several solver packages used by researchers in the field; there has been recently a push toward open-sourcing these packages. An excellent example is openCARP (https://opencarp.org/) (379), which has served the community broadly. A newcomer package is life^X^ (380).

#### 3.1.6. Additional developments in whole heart modeling, including ECG calculation.

In recent years advances have been made in modeling the functioning of the entire four-chamber heart (243), using CT scans to reconstruct the chambers’ geometry with biophysical representation of cell mechanics and cellular electrophysiology, although the applications have been predominantly geared toward heart mechanical activity. Modeling the heart extracellular potentials, such as electrograms on the epi- or endocardial surface (381), and body surface potentials and ECGs under normal and diseased conditions (382, 383) is also an important development with a significant potential for clinical utility. Electrograms and ECG signals are calculated with either a bidomain model or a simplified modeling approach that involves the integral solution to Poisson’s equation (384). The calculation of ECG using a multiscale organ-level model represents the addition of another spatial scale (the torso level) to the computational model.

### 3.2. Applications of Whole Heart Modeling in Arrhythmia and Electrophysiology

**FIGURE 10.**
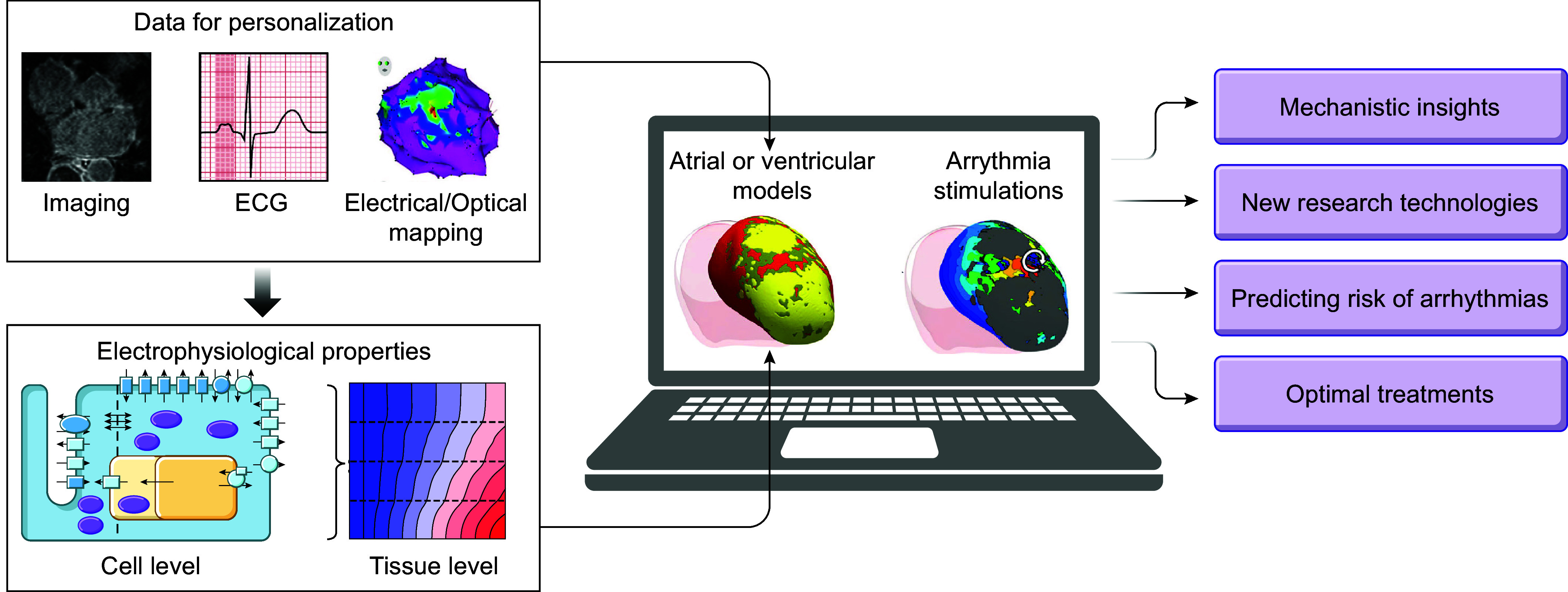
Use of atrial and ventricular organ-level modeling in arrhythmia and electrophysiology applications. A computational model of the ventricles and/or atria integrates multiple clinical and experimental data to provide an individualized virtual representation of the organ. Simulations using virtual hearts are used in arrhythmia and electrophysiology research with the 4 applications covered in this review.

The applications of whole heart modeling in arrhythmia research and clinical translation are wide-ranging and reflect the transformation of computational modeling of the heart from a basic science research tool, auxiliary to animal research, to a stand-alone methodology with significant contributions to novel approaches in the diagnosis and treatment of human arrhythmias and management of patients with heart rhythm disorders. In the sections below we structure the exposition in a manner that reflects this progression. FIGURE 10 summarizes the utilization of atrial and ventricular organ-level models in arrhythmia and electrophysiology applications. With models constructed as described in sect 3.1, computational modeling at the organ level has been used in four main areas: *1*) proving mechanistic insights, *2*) advancing and supporting new research technologies, *3*) predicting risk of arrhythmias and SCD, and *4*) guiding optimal treatment of heart rhythm disorders. The sections below review these four areas.

In these applications, there are several basic arrhythmogenesis concepts that we outline here. In the heart, arrhythmias are often caused and maintained by focal excitations, also referred to as premature atrial or ventricular complexes (PACs and PVCs, respectively) (385). When a vulnerable substrate is present, characterized by regional differences in refractoriness and/or excitability [due to disease-induced electrophysiological remodeling or as during the T wave of the electrocardiogram when some areas of the ventricle have repolarized but others have not (386)], a focal excitation can serve as the trigger of a reentrant activity. Whereas in the classical literature reentries have been classified as anatomical (around an obstacle such as scar) and functional (due to dispersion of electrophysiological properties), the contemporary understanding of clinical arrhythmias is that they incorporate both an anatomical and a functional component (387–389). Both PACs/PVCs and reentries result in activation of the heart with a higher frequency than normal sinus rhythm, underlying tachycardias. Studies have shown that a reentrant activity can break up into multiple rotational wavefronts through a variety of mechanisms (390), resulting in disorganized activity and fibrillation dynamics. The organizing centers of a reentrant activation, where the activation and repolarization waves meet, represent phase singularities, which can be determined by different approaches (391, 392). These concepts constitute the basic understanding of the important features of all arrhythmias in atria and ventricles alike and are referred to in most of the text that follows.

#### 3.2.1. Using computational modeling to elucidate the mechanisms of ventricular arrhythmias.

Whole heart modeling studies have revealed important mechanistic aspects of ventricular arrhythmias that could not have been established via experimentation alone. These studies rest on initial developments and insights obtained with simpler models, such as fibers, and tissue simulations in two and three dimensions, which have been reviewed previously (see extensive reviews in Refs. 16, 377, 393). Here we provide an overview of the arrhythmia mechanisms revealed predominantly by realistic-geometry computational modeling of the ventricles.

##### 3.2.1.1. elucidating the functional arrhythmia substrates in the ventricles.

Electrical alternans, i.e., beat-to-beat changes in APD, when occurring discordantly (out of phase in different regions of the heart), have long been recognized as a precursor to the development of lethal arrhythmias, as they set up a distribution of repolarization heterogeneity throughout the organ. Throughout the years, the restitution curve slope has been recognized as the main factor in both the onset of arrhythmias following the development of discordant alternans [see recent review by Qu et al. (386) for a detailed exposition of the mechanisms] and the dynamic destabilization of reentrant waves, leading to both the initiation of triggers and the transition of VT into VF (394, 395). Simulation studies using whole heart models have made important contributions to ascertaining the intricate set of mechanisms, including the effect of heterogeneous restitution, leading to these adverse events (394, 396–399). Ventricular computational studies conducted in the last decade have also represented the changes in Ca^2+^ handling associated with HF (400), leading to discordant alternans. Bayer et al. (340) elucidated the formation of reentrant waves and VF through the discordant alternans mechanism, where the steep repolarization gradients formed throughout the ventricles lead to arrhythmia initiation following pacing with a decreasing cycle length.

**FIGURE 11.**
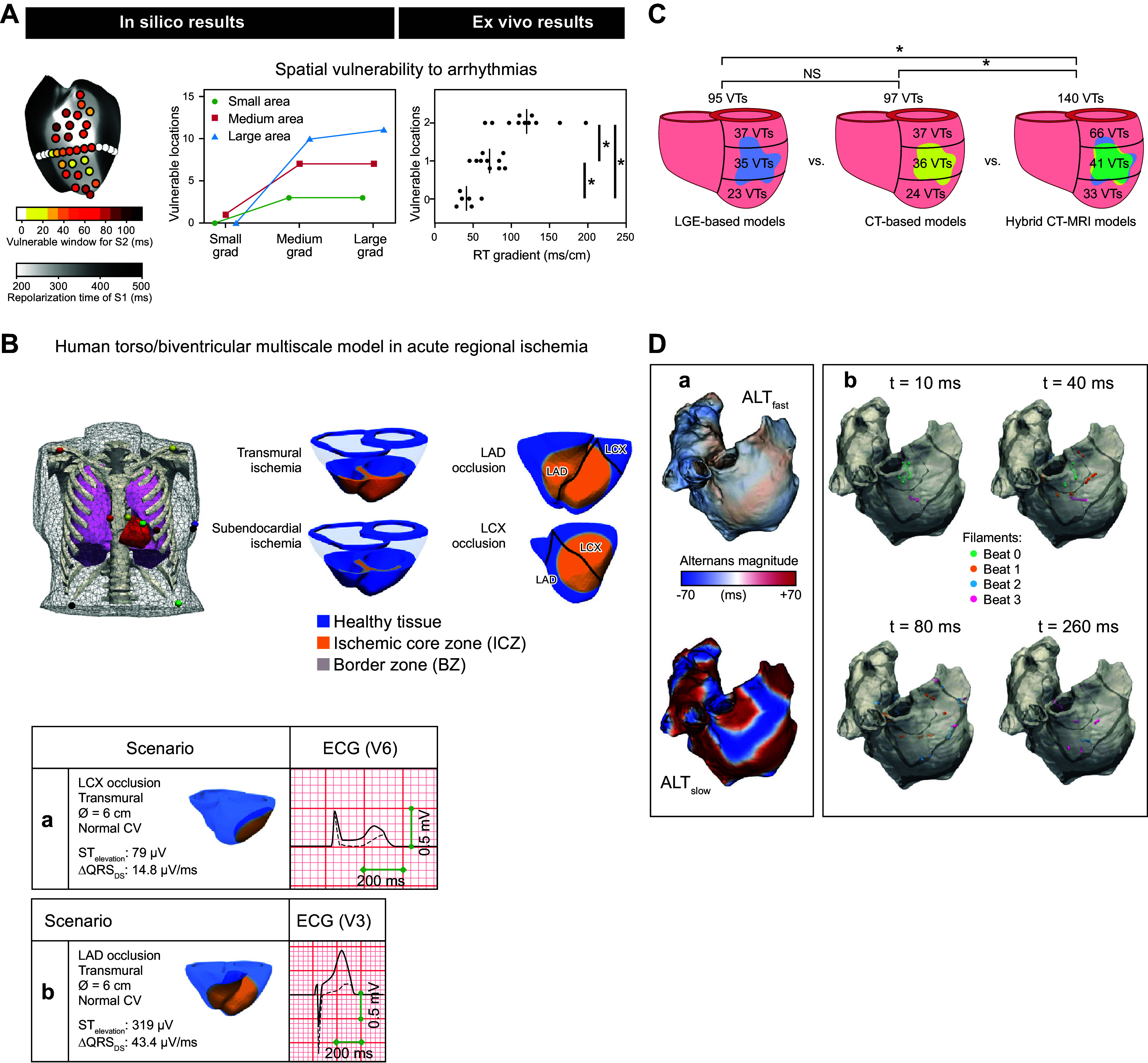
Arrhythmogenic mechanisms in the human ventricles and atria. *A*: vulnerability for arrhythmias increases with steeper repolarization time gradients and larger early-repolarization regions. *Left*: arrhythmia inducibility is tested for various locations; the color reflects the size of the temporal vulnerable window (white indicates that no arrhythmia could be induced). *Right*: this was tested for 9 combinations of small, medium, or large gradients and small, medium, or large early-repolarization time (RT) areas. The computational model demonstrates that the spatial vulnerable region increased with a larger size of the early-RT area. In explanted hearts, there was an increasing number of inducible pacing locations at the early-RT region with steeper repolarization time gradients. *P < 0.05. Modified from Ref. 401, with permission from *Science Translational Medicine*. *B*, *top*: human torso/biventricular electrophysiology model in acute regional ischemia for ECG and arrhythmia simulations. *Bottom*: ECG alterations caused by acutely ischemic regions, for left circumflex (LCX; *a*) vs. left anterior descending (LAD; *b*) artery occlusion. CV, conduction velocity; QRS_DS_, QRS downslope. Modified from Ref. 402, with permission from *Scientific Reports*. *C*: arrhythmogenicity of penetrating adipose tissue (inFAT) versus scar, shown with 3 different heart models. Number and distribution of ventricular tachycardias (VTs) across the 3 models are shown. LGE, late gadolinium enhancement. NS, not significant. Reproduced from Ref. 273, with permission from *Nature Cardiovascular Research*. *D*: arrhythmogenesis arising from calcium-driven alternans in a model of human atrial fibrillation. Shown are discordant action potential duration (APD) alternans and filaments during fast pacing. *a*: APD alternans magnitude in ALT_fast_ (*top*) and ALT_slow_ (*bottom*) atrial models during pacing at 270-ms cycle length. The 2 atrial models use 2 different membrane kinetics: ALT_fast_, with single-cell alternans occurring only at fast pacing rates (cycle length ≤250 ms) and ALT_slow_, with single-cell alternans occurring at slower pacing rates (≤400 ms). *b*: nodal surfaces (black) and filaments (color) in ALT_slow_ during 260-ms cycle length pacing. Snapshots show filament locations at different times after a paced beat from sinoatrial node region. Filaments from the first 4 beats are shown in different colors on the same snapshot for comparison. Reproduced from Ref. 403, with permission from *Scientific Reports*.

Repolarization abnormalities leading to idiopathic VF were examined by Cluitmans et al. (401) in a study that combined computational modeling with experiments and ECGI of survivors of idiopathic VF. FIGURE 11*A* shows a model of the human ventricle from this study with different gradients in repolarization time. The study found, importantly, that arrhythmias could only be induced from early repolarized regions. It also found that vulnerability to idiopathic VF increased with the steepness in repolarization time gradient and with the size of areas of early repolarization. Repolarization heterogeneity has also been implicated in the generation of polymorphic ventricular arrhythmias, such as torsades de pointes. Computational studies have explored the role of afterdepolarizations in propagation and the maintenance of these arrhythmias (404–406) as well as the risk factors modifying repolarization and leading to arrhythmogenesis in the setting of long QT syndrome. Specifically, Rivaud et al. (407) characterized repolarization duration and heterogeneity in relation to polymorphic VT (pVT) in the setting of LQTS. The study found that pVTs were inducible within a critical range of repolarization times and repolarization heterogeneities and are maintained by reentry wandering along the repolarization gradient. Liu et al. (408) offered a unified view of the mechanisms of spontaneous initiation of pVT in LQTS, suggesting that R-from-T was the likely common mechanism in LQT1, LQT2, and LQT3. In all three syndromes, PVCs always originated spontaneously from the steep repolarization gradient region and manifested on ECG as R-on-T (408). The authors referred to this mechanism as R-from-T, to distinguish it from the classic explanation of R-on-T arrhythmogenesis in which an exogenous PVC coincidentally encounters a repolarizing region. In R-from-T, the PVC and the T wave are causally related, where steep repolarization gradients combined with enhanced *I*_Ca,L_ lead to PVCs emerging from the T wave. Since enhanced *I*_Ca,L_ was required for R-from-T to occur, suppressing window *I*_Ca,L_ effectively prevented arrhythmias in all three genotypes.

Organ-level multiscale computational studies have also addressed some of the electrocardiographic aspects of this subject. Sadrieh et al. (409) explored the role of variation in the levels of ionic conductances that contribute to repolarization reserve in long QT syndrome and related those to features of the surface ECG. Epicardial dispersion of repolarization, consisting of regions of early and late repolarization, has been found to produce ECG waveforms exhibiting the typical Brugada syndrome morphology (410). Models have also been used to examine electrocardiographic signs of aberrant propagation patterns (411, 412) such as left bundle branch block (413), as well as to detect instabilities in the QT interval (414) and to investigate how variability in activation sequence and passive conduction properties translates into clinical variability in QRS biomarkers (383). Combined with an image-based torso representation, the human heart models have also evaluated the effect of heart position on ECG morphology in patients with HF and the signatures of LV hypertrophy in the ECG (415, 416).

Electrophysiological heterogeneities and arrhythmogenesis have also been shown to arise from microdomain-specific modulation of L-type Ca^2+^ channels (LTCCs) in human HF (355). t-Tubule loss in HF leads to altered LTCC function and early afterdepolarizations, which in turn propagate and initiate reentrant arrhythmias, as demonstrated by a complex subcellular-to-organ human ventricle computational model. The electrophysiological consequences of somatic mosaicism, the occurrence and propagation of genetic variation in cell lineages after fertilization, was investigated in the study by Priest et al. (417) of a 10-day-old infant with long QT syndrome. Rapid genome sequencing suggested a variant in the Na^+^ channel occurring in only 8% of the patient’s cells (417). A computational model of the infant’s heart that incorporated the Purkinje system demonstrated that this mosaicism resulted in 2:1 AV block and arrhythmia. Finally, computational modeling of the ventricles demonstrated that *I*_Na_ reduction precipitates source-sink mismatch, creating a potent substrate for sustained arrhythmia induction by PVCs originating near regions of ventricular wall expansion, such as the RV outflow tract (418).

##### 3.2.1.2. arrhythmogenesis due to commotio cordis and acute ischemia. role of the purkinje system in arrhythmogenesis.

Commotio cordis, mechanical induction of heart rhythm disturbances in the absence of corresponding structural damage (419, 420), is another case in which an arrhythmogenic substrate results from repolarization heterogeneity. In this case, the heterogeneity arises from mechano-electric feedback, and particularly from the recruitment of stretch-activated ion channels by the impact (421), which is particularly arrhythmogenic when it occurs during the T wave of the ECG. Organ-level computational models (422) have demonstrated how the region of impact is characterized by different types of cellular responses, including generation of a new action potential, shortening, or lengthening of repolarization, resulting in the initiation of a reentrant wave in the ventricles. By similar mechanisms, a VT can be terminated by a precordial thump (423); computational modeling has also provided mechanistic insight into precordial thump VT termination mechanisms and its reduced clinical utility in patients with global myocardial ischemia (424).

Acute myocardial ischemia arises from a mismatch between supply and consumption of oxygen due to narrowing/block of a coronary artery. The first 10–15 min, or phase 1A, of acute myocardial ischemia is particularly proarrhythmic (425) because of heterogeneity of repolarization and conduction (426, 427), allowing for functional reentries to arise. Specifically, ischemic regions in humans exhibit APD shortening, elevation of resting potential, prolonged postrepolarization refractoriness, and decreased conduction velocity, as demonstrated by extensive research (428–432). Ventricular models have been used to examine the arrhythmogenic properties of the acute ischemia functional substrate and the corresponding ECG patterns (402, 433–435). Among the findings was that Na^+^ channel availability increases the probability of ectopic beats in ischemia (434) and that there are two distinct mechanisms for subendocardial and transmural ischemic distributions: macroreentry around the region of transmural ischemia and microreentry in the ischemic border zone in subendocardial ischemia (402). In the latter study, a human torso/ventricular model was used to also examine the changes in the ECGs for left circumflex (LCX) and left anterior descending (LAD) artery occlusion (FIGURE 11*B*).

The Purkinje system is responsible for the rapid electric conduction in the ventricles. The Purkinje system has been implicated in the genesis of VT and VF as a source of ectopic beats, as well as in the reentry circuitry (436). Simulation studies have successfully incorporated a representation of the Purkinje system in whole heart and heart/torso models (437–439). Specifically, modeling has demonstrated that the structure of the Purkinje network and Purkinje-ventricular junctions could establish arrhythmogenic properties under diverse conditions (440). Purkinje system topology and the density of the Purkinje-ventricular junctions have been found to affect reentry dynamics (441). Purkinje-ventricular junctions have been found, in simulations, to be responsible for focal subendocardial activations during complex tachyarrhythmias (442) and for affecting reentry dynamics (441). EADs, which more readily originate in Purkinje fibers, have been shown to contribute to arrhythmia formation (443). Purkinje fibers have been shown to anchor transmural scroll waves and modulate repolarization heterogeneity (444, 445). Finally, as the Purkinje system is highly individualized, methodologies using simulations and clinical recordings have been developed to inversely estimate the Purkinje network from a patient’s electroanatomical map (446).

##### 3.2.1.3. arrhythmogenesis in ischemic and nonischemic cardiomyopathy.

Image-based computational heart models have emerged as important tools to investigate arrhythmia mechanisms arising from scar distribution in hearts with ischemic remodeling; a number of studies have focused on assessing the conducting channels in the scar and their involvement in VT. Simulations have provided insights beyond the structural analyses conducted on LGE-MRI images (which visualize scar distribution) and have allowed comprehensive evaluation of the ensuing VT circuits and the underlying mechanisms (447). Organ-level models have also explored the role of different cell types, such as myofibroblasts, in infarct-related arrhythmogenesis (447)

The structural characteristics of the scar surrounding the VT circuit were explored in ex vivo organ-level models constructed from submillimeter-resolution (0.25 × 0.25 × 0.5 mm^3^) ex vivo LGE-MRI scans (256). The VT conduction pathway was characterized by quantifying the local distance from the surrounding scar. Simulation results demonstrated the involvement of a subendocardial tissue layer of varying thickness in the majority of VT pathways. Importantly, the research revealed that VT pathways are most frequently established within thin surviving tissue structures of thickness ≤2.2 mm surrounding the scar, thus contributing toward a better understanding of infarct-related VT. Campos et al. (448) used individualized models of the LV to examine the electrophysiological factors that promote block and reentry in infarcted hearts, concluding that conduction velocity slowing in the infarct border zone was the dominant factor promoting arrhythmogenesis, with a contribution from the prolongation of APD in these regions. These results are consistent with the findings of Lopez-Perez et al. (449), who used a model constructed from a postinfarction patient scan and demonstrated that reduced conduction velocity and heterogeneity in APD in the border zone are the main factors promoting reentrant activity. The mechanisms of conduction block in the infarct border zone were investigated in a recent study (450), demonstrating that subthreshold DADs lead to conduction block in narrow isthmuses where electrotonic load is lessened by the nonconducting scar. Recently O’Hara et al. (451) demonstrated that prolongation of repolarization in regions adjacent to the infarct may prevent reentry and postinfarction VT. Finally, high-resolution models reconstructed from ex vivo data have investigated the effect of image resolution on the VT pathways in human (clinical) models by downsampling the high-resolution images to lower-resolution images and then simulating and comparing the corresponding VTs (284). Simulation results demonstrated that all models, regardless of image resolution, accurately predicted the VT circuit location, paving the way for clinical applications.

In patient-specific computational models of infarction, where clinical LGE-MRI is used, the infarct border zone is identified in the images as gray zone, a tissue with an intermediate signal intensity between scar and noninjured myocardium. Simulations found that arrhythmia activity was primarily concentrated in the gray zone and was largely dependent on the morphology and size of these remodeled tissue regions rather than on variations in the structural heterogeneity of the gray zone (328, 452). Increased gray zone was found to destabilize VT circuits (453), with some geometric configurations being more arrhythmogenic than others (454). Conducting channels in the infarct were found to have three distinct phenotypes: I-type, T-type, and functional-type channels, depending on the VT circuit and the critical channel morphology (455).

inFAT, also called lipomatous metaplasia, has been found to penetrate the myocardium and colocalize with scar in patients with older infarcts, >3 yr of age; there is also an increased infarct-related arrhythmogenesis at that time after infarction (456, 457). However, because inFAT is intermingled with scar, the specific role of inFAT in ventricular arrhythmia propensity is difficult to discern. Clinically, inFAT is identifiable on CE-CT (458). Using personalized computational heart models reconstructed from both LGE-MRI and CE-CT, as well as the combination of the two imaging modalities, Sung at al. (273) explored arrhythmia induction in the different model types (FIGURE 11*C*). Importantly, using these models and clinical measurements of electrophysiological abnormalities, the study demonstrated that inFAT, rather than scar, is a primary driver of arrhythmogenic propensity at that age of the infarct and is frequently present in critical regions of the arrhythmia circuit. These computational findings implicated inFAT as a dominant player in infarct-related arrhythmias, challenging existing paradigms. Computational predictions have been recently verified in clinical studies (308, 459).

Finally, computational research has also explored arrhythmia mechanisms in nonischemic cardiomyopathy, which arises from the disease-induced structural and electrophysiological remodeling of the substrate. The pattern of fibrosis, together with the degree of gap junction uncoupling, has been shown to determine the level of organization of ventricular arrhythmias (460). Specific cardiac diseases associated with ventricular arrhythmias that have been investigated by LGE-MRI-based ventricular models include myocarditis, repaired tetralogy of Fallot (rToF), cardiac sarcoidosis (CS), HCM, as well as arrhythmogenic (right ventricular) cardiomyopathy (ARVC or ACM) (262, 263, 275, 285, 286, 461). Computational models of rToF and CS included not only the left but also the right ventricle, often ignored in ventricular computational studies as its thinner walls are often difficult to reconstruct from LGE-MRI studies. The CS study also explored the electrophysiological effect of inflammation (as visualized on PET scans), using hybrid ventricular models reconstructed from PET and LGE-MRI images, and how inflammation combines with fibrosis distribution to promote arrhythmogenesis.

**FIGURE 12.**
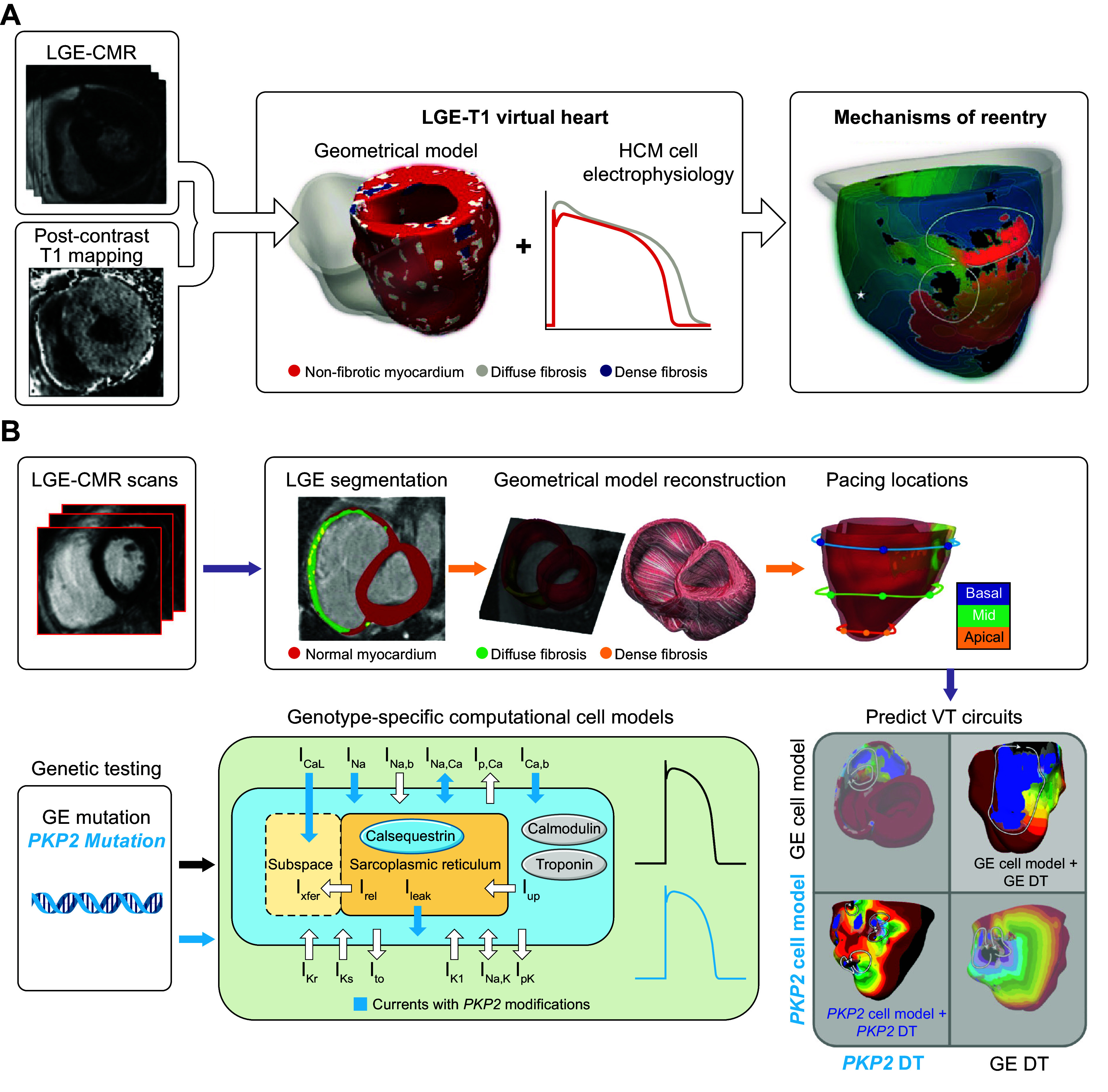
Exploration of arrhythmogenic mechanisms in nonischemic cardiomyopathy. *A*: flowchart summarizing the study of ventricular arrhythmias in hypertrophic cardiomyopathy (HCM) patients. A combination of late gadolinium enhancement (LGE)-cardiac magnetic resonance (CMR) imaging and postcontrast T1 mapping is used to construct personalized left ventricular (LV) geometrical models with diffuse and dense scar. Incorporating HCM-specific electrophysiological properties (action potential kinetics, conduction velocity) completes the generation of each ventricular model, which is then used to study arrhythmia mechanisms. Modified from Ref. 262, with permission from *eLife*. *B*: flowchart summarizing the workflow of using a genotype patient-specific biventricular model, i.e. “digital twin” (Geno-DT) to understand arrhythmogenesis in arrhythmogenic cardiomyopathy in 2 genotype groups, plakophilin-2 (PKP2) and gene-elusive (GE). Orange blocks refer to clinical data, which include genetic testing results and LGE-CMR images for each patient in the cohort. Patient-specific geometrical heart models were reconstructed from the LGE-CMR (*top*, purple, *left* and *center* images). Genotype-specific cell models (green) developed here were incorporated into each heart model based on the patient’s genetic testing result. The integrated multiscale patient-specific Geno-DT models were used to understand the role of remodeling in arrhythmogenesis and to predict ventricular tachycardia (VT) circuits (*bottom*, gray). Modified from Ref. 461, with permission from *eLife*. *I*_Ca,b_, background Ca^2+^ current; *I*_Ca,L_, L-type Ca^2+^ current; *I*_K1_, basal inward-rectifier K^+^ current; *I*_Kr_, rapid delayed-rectifier K^+^ current; *I*_Ks_, slow delayed-rectifier K^+^ current; *I*_leak_, Ca^2+^ leak from sarcoplasmic reticulum; *I*_Na_, Na^+^ current; *I*_Na,b_, background Na^+^ current; *I*_Na,K_, Na^+^-K^+^-ATPase current; *I*_Na,Ca_, Na^+^/Ca^2+^ exchange current; *I*_p,Ca_, plasmalemmal Ca^2+^-ATPase current; *I*_rel_, Ca^2+^ release flux from the sarcoplasmic reticulum; *I*_to_, transient outward K^+^ current; *I*_up_, Ca^2+^ uptake flux into the sarcoplasmic reticulum; *I*_xfer_, transfer of Ca^2+^ from subspace to cytosol.

In the HCM study (262), both diffuse fibrosis, as visualized on T1 mapping, as well as focal scar from LGE-MRI were incorporated in the hybrid LGE-T1 ventricular models (FIGURE 12*A*). These multiscale ventricular computational models incorporated regional HCM ionic remodeling based on cellular experimental recording from septal resection preparations as well as remodeling of the gap junctions in the regions of fibrosis. Interestingly, the approach used the T1 mapping to incorporate additional personalization information regarding the patient-specific fibrosis segmentation thresholds. This research demonstrated that the presence of diffuse fibrosis, which is rarely assessed in these patients, increases arrhythmogenic propensity.

Finally, in the ARVC study Zhang et al. (461) developed a novel genotype-specific ventricular modeling approach to investigate the role of pathophysiological remodeling in sustaining VT and to predict the VT circuits in ARVC patients of different genotypes (FIGURE 12*B*). This approach incorporated genotype-specific cellular electrophysiological properties, with the multiscale character of the model emphasized in FIGURE 12*B*. By comparing the predicted VT circuits to the clinical observations, the study revealed that the underlying VT mechanisms differ among ARVC genotypes. In genotype-elusive patients fibrotic remodeling was found to be the primary contributor to VT circuits, whereas in *PKP2* patients slowed conduction velocity and altered restitution properties of cardiac tissue, in addition to the structural substrate, were directly responsible for the formation of VT circuits. When the genotype of the patient was switched, the model could no longer correctly predict the VT circuits (see FIGURE 12*B, bottom right*). This is the first study to include personal genetics in the patient-specific heart models and to demonstrate its importance in the arrhythmogenic substrate in nonischemic cardiomyopathy.

##### 3.2.1.4. defibrillation mechanisms.

Ventricular models incorporating realistic geometry have also been used to examine the mechanisms by which a defibrillation shock induces arrhythmia and defibrillates (terminates VF) in the heart. As a matter of fact, bidomain models of the ventricles were some of the first biophysically detailed organ-level models developed (462), helping to develop and establish the so-called “virtual electrode hypothesis for defibrillation” (463, 464), according to which the defibrillation shock establishes regions of positive and negative polarization in the myocardium that determine the postshock activity in combination with the distribution of transmembrane potentials in the myocardium (465). A series of ventricular computational studies determined the mechanisms by which a defibrillation shock fails or succeeds in terminating VF. A ventricular model (466, 467) was used to determine the mechanisms, including those that are a function of geometry, by which virtual electrode polarization is induced after a defibrillation shock and those by which postshock activations originate (466–469). In studies of arrhythmogenesis with external monophasic shocks, Rodriguez et al. (470, 471) demonstrated that shock outcome and the type of postshock arrhythmia induced by the shock depend on the location of the intramural postshock excitable area formed by shock-induced deexcitation of previously refractory myocardium. Ashihara et al. (472) extended these findings, suggesting that the mechanism that underlies the quiescent period (this period is termed the isoelectric window) following strong shocks, of strength near the upper limit of vulnerability or the defibrillation threshold, is attributable to “tunnel propagation” of postshock activations through intramural excitable areas in the 3-D ventricles. This theory was extended to explain the mechanisms responsible for the existence of the isoelectric window following ICD shocks delivered to the fibrillating heart (473). The simulation results demonstrated that the nonuniform field created by ICD electrodes, combined with the fiber orientation and complex geometry of the ventricles, resulted in a postshock excitable region always located in the LV free wall, regardless of preshock state. The mechanisms underlying initiation of postshock arrhythmias with electrical shocks under the conditions of global ischemia have also been studied (474). Computational modeling has also been used to propose new types of defibrillation delivery, such as low energy or high frequency (475–478).

#### 3.2.2. Using computational modeling to elucidate the mechanisms of atrial arrhythmias.

The growing burden of AF, the most common human arrhythmia, and the health care expenditures associated with its treatment have provided a massive impetus to computational modeling of the atria, in an attempt to provide understanding of how functional and structural remodeling result in the turbulent propagation associated with AF (23, 124, 479). Atrial computational models have made major contributions in revealing how intrinsic atrial structural and electrophysiological heterogeneities predispose to atrial arrhythmias.

##### 3.2.2.1. intrinsic electrophysiological heterogeneities in the atria and arrhythmogenesis.

Even in the structurally and electrophysiologically normal atria, the complex geometry of the chambers, with distinct structural features such as orifices and discrete bundles, anisotropic conduction in structures like the crista terminalis, as well as intrinsic differences in electrical properties (e.g., APD), has been found by a number of atrial computational models (16, 23, 480) to present a substrate that predisposes to arrhythmogenesis (16, 23, 481). Organ-level computational research (481, 482) has demonstrated that because electrotonic coupling transverse to the crista terminalis is weak, high-frequency pacing in the vicinity results in a reduced safety factor, leading to unidirectional block and generation of reentry. Additionally, the muscular sheath of the coronary sinus was found to act as a pathway for reentry (52). The complex myocyte arrangement in the posterior LA was found to contribute to dispersion in activation times and to increased vulnerability to arrhythmia (483, 484). The openings of the inferior and superior venae cavae (52) and of the tricuspid valve (485) were shown to anchor atrial flutter. The arrhythmogenic conditions associated with nonuniform vagal stimulation were also explored (486), as well as the role of the ganglionic plexuses in initiating AF (487).

Further contributions include investigating the role of pulmonary vein triggers in initiating paroxysmal AF (488), establishing the mechanisms for atrial alternans (403, 489), and revealing the contribution of the electrical uncoupling between the endocardial and epicardial layers (293). An example of atrial arrhythmogenesis arising from Ca^2+^-driven atrial alternans is shown in FIGURE 11*D*, where discordant (out of phase) alternans result in the formation of filaments (organizing centers of reentry). Furthermore, with the use of models with both idealized and realistic atrial geometry, atrial wall thickness heterogeneity has been demonstrated to have a significant impact on AF reentrant drivers’ trajectory and localization (490, 491). Genetic mutations, particularly in KCNH2, KCNQ1 and KCNJ2 genes, shorten APD; atrial models incorporating gain-of-function mutations in these genes have exhibited greater inducibility of reentry and increased life span of reentrant drivers (492–494). Additionally, atrial stretch is believed to participate in the onset and maintenance of AF, as acute atrial stress is associated with conduction slowing and complex signal formation and prolongation of atrial refractory periods (495, 496). Computational modeling has been used to integrate the cell-level electrophysiological alterations induced by atrial stretching with the distribution of stretch in the atria. Higher strain has been found to occur in areas adjacent to the mitral valve annulus, rim of the appendage, pulmonary vein trunks, and Bachmann’s bundle, regions where atrial arrhythmias are most likely to occur (497). Finally, populations of ionic models calibrated to recordings from patients with AF have been used in 3-D geometry models (498, 499) to provide further insights in the mechanisms of AF maintenance. The same inhibition of ionic currents was able to produce different effects on AF dynamics in atrial simulations using cells modeled to have the same variability as human experimental data (483). The next frontiers in development of cohort-specific model populations are to develop model populations calibrated to different AF subtypes according to AF burden (i.e., paroxysmal, persistent, and long-standing persistent AF) and use them in simulations to gain subtype-specific mechanistic insights and to incorporate biomarkers that further refine model selection by describing more specific disease phenotypes.

##### 3.2.2.2. structural remodeling in the human atria and arrhythmogenesis.

An important advancement in the understanding of AF mechanisms was the discovery of fibrotic remodeling in the atria of AF patients (321, 500). LGE-MRI studies have revealed that every patient with AF presents some degree of fibrotic remodeling (501, 502). Atrial modeling studies strongly support that the extent and distribution of atrial fibrosis are critical determinants of AF initiation, maintenance, and reentrant driver dynamics during AF. In a sensitivity analysis of simulations using realistic atrial geometry, the extent and distribution of fibrosis had a greater impact on reentrant driver localization over alterations in tissue wavelength (315). Although presence of a certain amount of fibrosis is sufficient for initiation of AF in simulations, patient-specific fibrosis distribution determines reentrant driver dynamics (54, 503, 504). In two separate studies using patient-specific atrial geometry and fibrosis distribution derived from LGE-MRI, the reentrant drivers that occurred during AF localized in the boundary zones between fibrotic and nonfibrotic atrial myocardium (505, 506). These zones had a highly specific fibrosis spatial pattern, characterized by high fibrosis density and entropy, and corresponded to atrial areas with a high degree of intermingling between fibrotic and nonfibrotic atrial myocardium (506).

However, the relatively low resolution of in vivo LGE-MRI scans results in the segmentation of atrial fibrosis from LGE-MRI as well as the delineation of the thin atrial wall being fraught with uncertainty and is an area of intense research. Ex vivo studies provide additional information with submillimeter-resolution data and histological analysis. The association between patient-specific fibrosis distribution and reentrant driver dynamics has been experimentally validated in a single ex vivo atrial preparation from a patient with long-standing persistent AF. A 3-D atrial computational model based on histologically validated LGE-MRI and panoramic optical mapping at submillimeter resolution allowed for integration of detailed atrial anatomy as well as functional data (regional APD, conduction velocity, and rate-dependent behaviors); the results indicated that atrial structural fingerprints, consisting of a specific combination of intermediate wall thickness, intermediate fibrosis, and twisted myofiber orientation, can be used to identify AF drivers (257). Future studies, however, are needed to better ascertain the association between reentrant driver dynamics and fibrosis as well as the contribution of reentrant drivers to AF pathophysiology, as it remains controversial (507). Finally, a computer model of human AF that represented only epicardial fibrosis provided a potential explanation and demonstration of endo-epicardial dissociation and epicardial breakthroughs (294).

#### 3.2.3. Using computational modeling to develop and advance new antiarrhythmia technologies.

Whole heart models are an effective tool to test hypotheses and explore new research technologies. Using computational modeling to explore different scenarios while testing new technologies is an inexpensive way to make advancements and to improve the technology. Here, we review the role of computational heart modeling in advancing two new technologies, namely regenerative therapies and cardiac optogenetics.

Organ-level computational models have been used to assess arrhythmogenicity of biological substrate modification therapies. Specifically, cell-based cardiac regenerative therapies, a promising treatment to reverse cardiac remodeling in the postinfarct heart, have been found to be arrhythmogenic. However, why these newly engrafted cells can be arrhythmogenic is poorly understood. To better elucidate these mechanisms, a computational modeling framework to simulate the consequences of different cell-based therapy modalities was developed (287). Patch engraftment in the ventricles was simulated and the subsequent arrhythmogenicity evaluated. Several unprecedented arrhythmogenic mechanisms of stem cell engraftment were revealed by the simulation results. For hIPSC-CM injection, parameters such as injection location, cell dosage, and engraftment spatial distribution were found to be decisive in the occurrence of ectopic propagations. In hIPSC-CM cell sheet transplantation, computational models showed that engraftment location and its impact on substrate heterogeneity primarily determined VT inducibility. Assessing the arrhythmogenic effects of stem cell-derived cardiomyocyte engraftment in models with patient-specific fibrotic distributions (508), computational research found that arrhythmias arising from engraftment were likely to be from reentrant, not focal, activity and that the location of the patch engraftment relative to the patient-specific fibrotic distribution was important in determining arrhythmogenicity. Ventricular heart models were also used to explore the mechanisms of biological substrate modification by exosome injection for the prevention of recurrent ventricular tachyarrhythmias (509).

Cardiac optogenetics is an emergent research area involving the delivery of light-sensitive proteins (opsins) to excitable heart tissue to enable optical modulation of cardiac electrical function, in the hope of achieving a selective regional electrophysiological modulation. Recent applications of optogenetics include contactless assays for quantification of electrophysiological properties; optogenetic perturbation of cardiac tissue to unveil mechanistic insights on the initiation, perpetuation, and termination of arrhythmia; and potential translational innovations such as light-based pacemaking and defibrillation (510–512). Organ-level computational modeling has also made significant contributions to advancing optogenetics applications exploring the feasibility of optogenetics-based termination of AF (513, 514), as well as the potential for optogenetic defibrillation (515, 516).

#### 3.2.4. Clinical translation of applications of heart modeling in arrhythmia and electrophysiology.

In the last decade, a significant advancement in computational modeling of arrhythmias and electrophysiology has taken place, in which heart models have transitioned to utilization in clinical applications (16, 17, 517, 518). This development is of significant pride to the computational research community, marking a level of sophistication of the human heart models that makes them suitable for clinical applications. Below we review two major clinical applications of computational modeling: the prediction of SCD due to arrhythmias in various diseases and the use of computational modeling to improve arrhythmia treatment by catheter ablation.

##### 3.2.4.1. assessment of risk of sudden cardiac death due to arrhythmias in different diseases.

The incidence of SCD is increasing globally. A large number of SCDs result from ventricular arrhythmias (SCDA), particularly among patients with prior heart disease. The cornerstone for primary prevention of SCDA is ICD implantation (519). Although the survival benefit is indisputable, ICD therapy is resource intensive, and there are risks of complications and morbidity (520). Accurate SCDA risk assessment before device deployment is crucial. Currently, this decision is based on a single metric, LV ejection fraction (LVEF) < 35% (521), which is insensitive (522). Many patients are exposed to ICD risks without deriving any health benefit (the majority of ICDs implanted are never utilized), whereas others are not protected, often dying suddenly in the prime of their life (523, 524). Accurate individualized SCDA risk assessment remains a major unmet need.

Mechanistic computational modeling of the heart has made major strides in predicting personalized risk of SCDA in patients with ischemic and nonischemic cardiomyopathies. These were the first studies in the field of cardiac computational modeling in which a relatively large number of patient-specific models were reconstructed. The landmark study by Arevalo et al. (2016) (264) was a retrospective study of 41 postinfarct patients with reduced LVEF (<35%), all of whom underwent ICD implantation. Multiscale computational models were reconstructed from the clinical LGE-MRI images and electrophysiological abnormalities (altered ionic models) and gap junction remodeling included in regions of structural remodeling (i.e., gray zones, etc.), resulting in global electrophysiological heterogeneities throughout the ventricles. Pacing protocols adapted from clinical procedures were then applied to induce VT in these virtual patients’ hearts. The outcome of these virtual electrophysiological studies, i.e., whether arrhythmia occurred or not in the infarct-remodeled ventricular substrate, was then used to establish a patient’s arrhythmic risk. This noninvasive approach to SCD risk stratification was termed the virtual heart arrhythmia risk predictor (VARP). The study demonstrated that virtual heart inducibility was more predictive for risk of arrhythmia than existing standard clinical metrics. Patients with inducible virtual hearts were more likely to correspond to those who suffered arrhythmic outcomes than patients with noninducible virtual hearts. This study highlighted how the virtual heart approach could be used to determine a patient’s arrhythmia risk. In a separate proof-of-concept study, VT risk was investigated in a small cohort of myocardial infarction patients (*n* = 4) with preserved ejection fraction (525). Even though the patients in this cohort were not candidates for ICD placement, one patient had a VT history and the personalized simulation results matched the clinical result, indicating the generalizability of using virtual electrophysiological studies to assess arrhythmia risk.

The virtual heart approach has also been utilized for SCDA risk prediction in patients with nonischemic cardiomyopathies, similarly accounting for structural and, accordingly, regional electrophysiological remodeling. In a pediatric cohort with acute myocarditis (*n* = 12) (286), computational modeling to predict SCDA correctly predicted high risk for arrhythmia and outperformed the clinical metrics. The main issue with using LGE-MRI for myocarditis model construction is that it cannot distinguish acute (edema) and chronic (scar or fibrosis) states of myocardial injury from myocarditis. A similar approach was taken for patients with rTOF (285), i.e., patients who underwent surgical intervention in their childhood. These patients are at higher risk for arrhythmia due to scarring from the surgery. In a cohort of seven patients, all of whom were deemed to be at low risk according to the clinical guidelines (prolonged QRS duration), simulations revealed that two of these patients had a high propensity to arrhythmia. Indeed, it turned out that these two patients had subsequent clinically detected VT.

HCM, a common genetic disease characterized by thickening of cardiac muscle, is also associated with high SCDA risk arising from the proliferation of fibrosis in the heart. Current clinical risk stratification criteria inadequately identify at-risk patients in need of primary prevention of VT. The studies by O’Hara et al. (262, 275) developed a personalized strategy to forecast risk of ventricular arrhythmias in these patients. The authors combined LGE-MRI and T1 mapping data to construct fusion heart models (*n* = 26) that represent the patient-specific distribution of both focal and diffuse fibrosis. In forecasting future arrhythmic events in HCM patients, the computational approach significantly outperformed current clinical risk predictors. In a different HCM study (526), medical images were integrated with ECG data to classify, with machine learning, patients into different HCM phenotypes and evaluate the risk associated with each phenotype. In that study, computational modeling was used to understand the mechanisms underlying the abnormal ECG patterns.

Another nonischemic cardiomyopathy associated with high SCDA risk is CS, an inflammatory disease. Shade et al. (263) presented a two-step prediction approach, combining computational modeling with machine learning. First, a patient’s arrhythmogenic propensity arising from CS-induced ventricular remodeling was assessed with a novel fusion model, based on LGE-MRI and PET, and incorporation of electrophysiological heterogeneities in the regions of inflammation and fibrosis. The results from simulation of arrhythmia inducibility were fed, together with a set of imaging and clinical biomarkers, into a supervised classifier. In a retrospective study of 45 patients, the technology outperformed clinical metrics, highlighting its potential to transform CS risk stratification.

**FIGURE 13.**
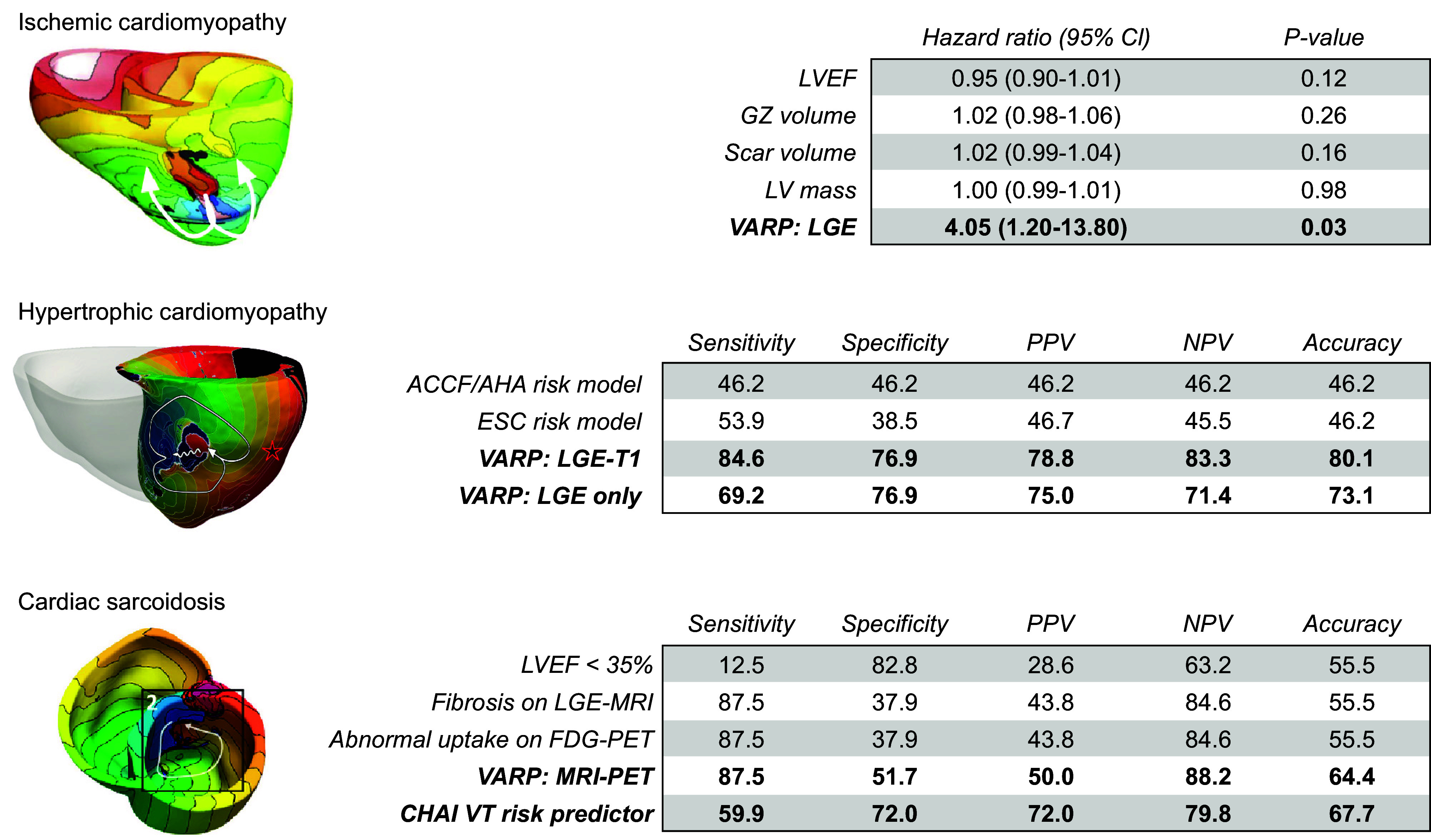
Predicting sudden cardiac death from ventricular arrhythmias (SCDA) risk in patients with different arrhythmogenic heart diseases. Arrhythmias in the different models are shown on *left* for each heart condition. White arrows indicate the ventricular tachycardia (VT) reentrant circuit in each model. ACCF/AHA, American College of Cardiology Foundation/American Heart Association; CHAI, Computational Heart and AI risk predictor; CI, confidence interval; ESC, European Society of Cardiology; FDG, fluorodeoxyglucose; GZ, gray zone; LGE, late gadolinium enhancement; LVEF, left ventricular ejection fraction; NPV, negative predictive value; PPV, positive predictive value; VARP, virtual heart arrhythmia predictor. Modified with permission from Ref. 264, with permission from *Nature Communications*; Ref. 262, with permission from *eLife*; and Ref. 263, with permission from *Scientific Advances*.

FIGURE 13 summarizes the predictive capabilities of ventricular computational models for arrhythmogenesis in different heart diseases. The tables in FIGURE 13 combine comparisons between the model predictions and common clinical risk predictors, as presented in the original publications; the superior performance of the modeling approach is highlighted in bold.

These novel SCDA risk assessment approaches using mechanistic computational modeling of the heart have achieved excellent results, outperforming clinical metrics by broad margins, and thus have the potential to prevent SCDA and help deploy primary prevention appropriately in patients with different heart diseases. The downside of these approaches is that they are computationally intensive, limiting the applicability to small patient populations (527). Applications of AI approaches for SCDA prediction (528) and use of computational modeling for providing interpretability and mechanistic insight as to how the AI-identified features contribute to SCDA might a be a successful pathway forward.

##### 3.2.4.2. computational assistance and guidance for ablation of ventricular tachycardias.

Catheter ablation is a major component of contemporary management of VTs. This minimally invasive procedure involves the use of catheters that are maneuvered into the cardiac chambers. To terminate ventricular arrhythmias, radiofrequency energy is delivered to specific areas. Identification of these specific locations in the ventricles is, however, not straightforward and requires characterization of the arrhythmogenic substrate through the laborious process of acquiring electrical signals on the surfaces of the ventricle, i.e., electroanatomic mapping (EAM). Ablation lesions are then delivered at sites of abnormal electrical signals according to the EAM, although what defines an abnormal electrical signal remains a subject of dispute. The ablation procedure is thus time consuming; importantly, it does not guarantee VT termination, as ablation targets can be inaccurate or new (emergent) VTs can arise in the modified-by-ablation substrate at different time points after ablation. Patient-specific computational heart modeling can aid in improving ablation precision by proposing ablation targets, providing noninvasive localization of abnormal electrical signals, and/or identifying VT exit sites on the cardiac surfaces.

**FIGURE 14.**
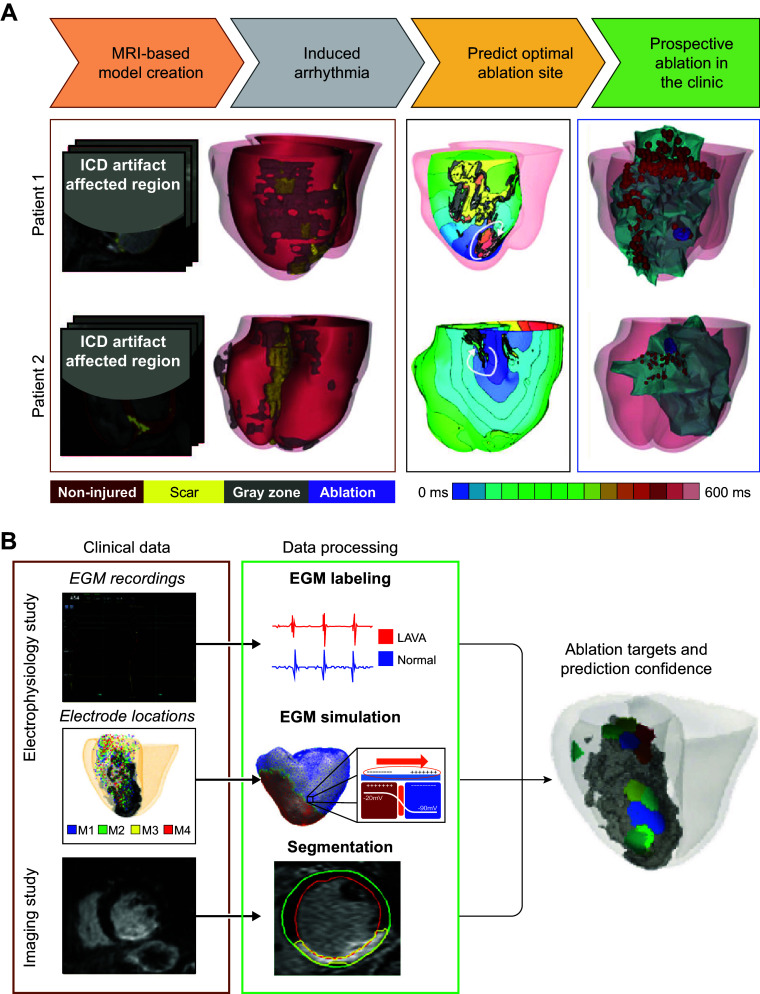
Computational assistance in assessing reentrant circuits and guiding ventricular ablation. *A*, *top*: a flowchart summarizing the protocol (arrowed steps) and the retrospective and prospective studies. *Bottom*: results from the prospective human study, showing simulation-guided ablation for 2 patients with implantable cardioverter defibrillators (ICDs). *Left*: reconstructed ventricular models with different remodeled regions. *Center*: activation maps corresponding to the 2 ventricular tachyarrhythmia (VT) morphologies induced in the 2 patient heart models. White arrowheads depict the direction of propagation of the excitation wave. The color scale indicates activation times, and black indicates tissue regions that did not activate. *Right*: simulation-predicted ablation targets for the 2 VT morphologies. Coregistration of the simulation-predicted targets (purple) with the CARTO 3 endocardial surface (green). The red dots correspond to locations of the tip of the catheter during ablation. The left ventricular endocardial surface is shown in green, and the total infarct region is shown in gray. Noninjured and scar tissues are shown in red and yellow, respectively. Modified from Ref. 258, with permission from *Nature Biomedical Engineering*. *B*: model-based feature augmentation for ablation target learning from images. Modified from Ref. 260, with permission from *Circulation: Arrhythmia and Electrophysiology*. The pipeline shows the clinical data and the data processing, feature extraction, and learning stages. It shows electrogram (EGM) labeling (local abnormal ventricular activities, LAVA) used in the training process. Both simulated and image features are fed into the random forest algorithm for training. Ablation targets are predicted with the confidence in prediction provided. Modified from Ref. 529, with permission from *IEEE Transactions on Biomedical Engineering*.

In terms of identifying optimal ablation targets to terminate VT, a retrospective study employing patient-specific models from 13 postinfarct patients who underwent ablation showed that ablation targets from simulations were consistent with the regions targeted clinically (53). This work was extended into the first prospective study that used computational modeling to predict the ablation targets and guide the ablation in patients with ischemic cardiomyopathy (258); see FIGURE 14*A* for the predicted VT circuits, and the executed lesions, shown in the EAM, guided by the computational model. In addition to the 5 prospective patients, the study included 21 retrospective patients, where predicted targets were compared to the clinical targets, as well as animal studies. This work highlighted the enormous potential for virtual heart technology to impact the clinical management of ventricular arrhythmias. Further sensitivity analyses where electrophysiological properties were varied in the models demonstrated that the predictions of the ablation targets were rather robust (530).

Patient-specific heart models reconstructed from clinical images have also been used to better understand VT circuit morphology (449, 531). Sung et al. (532) demonstrated that inclusion of repolarization gradients, both transmural and apicobasal, altered VT circuit morphologies, with minimal change of the ablation targets. MRI-based virtual heart simulations of VT circuits have also been combined with an automated ECG-based localization algorithm (533) to predict VT exit sites, highlighting a potential synergism between the two methodologies. Computational studies have also evaluated heterogeneity in APD as a VT susceptibility metric. A technique called reentry vulnerability index (RVI) has been studied (534), which pinpoints the slow-conducting and abnormal repolarized sites; this technique is particularly useful in arrhythmias that are not scar related. In a porcine heart model, RVI identified ablation targets and endocardial electrogram features (381). A recent study further developed and validated the approach in patient-specific computational models of postinfarct VTs, demonstrating that RVI mapping allows localization of multiple regions susceptible to reentry (535).

Patient-specific computational models have also provided noninvasive characterization of the electrical substrate to aid in preprocedural ablation planning. Monaci et al. (536) provided a framework for comparing whole heart simulations of pace mapping, a clinical electrophysiological technique used during substrate mapping to localize the VT exit sites, with clinically recorded electrograms to aid preprocedural planning. Efforts have also been made to reproduce with patient-specific simulations the intracardiac electrograms that would be recorded during substrate mapping. In one such study, patient-specific heart models successfully reproduced the abnormal patterns in intracardiac electrograms, which could represent targets for ablation (537). The authors further advanced this methodology by combining this biophysically detailed modeling approach with machine learning to accurately distinguish between normal and abnormal electrogram signals, in an important advancement in the clinical translation of computational heart modeling approaches (529); FIGURE 14*B* presents a schematic of this approach. In the clinic, multiple wavefront pacing (MWP) and decremental pacing (DP) are two EAM strategies that have emerged to better characterize the VT substrate. A recent patient-specific computational modeling study on 48 patients assessed how well MWP, DP, as well as other techniques used in clinical studies improve identification of electrophysiological abnormalities on EAM that reflect infarct remodeling and critical VT sites (538). The study found that EAM with MWP is more advantageous for substrate characterization in hearts with less remodeling. During substrate EAM, MWP and DP should be combined and delivered from locations proximal to a suspected VT circuit to optimize identification of the critical VT site.

In addition to LGE-MRI, CT has also been used to construct virtual heart models for preprocedural assessment of the arrhythmogenic substrate (271). In a study of five postinfarction patients, the scar was modeled with reduced conduction speed as a function of myocardial wall thickness. This computational workflow was designed to be efficient and integrated into clinical workflows as a noninvasive, intraoperative mapping tool for ablation therapy. Infiltrating adipose tissue has also been identified as potential proarrhythmic substrate based on intensity values on CT and has been combined with virtual heart technology to predict VT ablation targets in a retrospective cohort of 29 postinfarct patients who underwent VT ablation (260). The study provided examples of predictions showcasing the advantages of personalized computational modeling, as the model predicted not only the targets on index ablation but also the ablation targets on a redo procedure in patients as late as 4 yr after index ablation. Overall, the ablation targets predicted by the virtual hearts were consistent with the clinical ablation targets and encompassed much smaller lesion volumes. Since CT is accessible to a broad range of clinical centers, such technologies could be readily deployed prospectively to improve VT ablation. Finally, the virtual heart approach can also be used to assess the efficacy of emerging clinical technologies, such as the success of augmented reality-based catheter navigation systems (539), and has demonstrated how the augmented reality system could improve ablation targeting.

##### 3.2.4.3. computational assistance and guidance for ablation of atrial arrhythmias and atrial fibrillation.

Perhaps the most exciting aspect of personalized models of AF is the ability to simulate, test, and plan different AF ablation strategies prospectively and even predict a patient’s risk of AF recurrence. Following the seminal work of Haissaguerre et al. that led to pulmonary vein (PV) isolation (PVI) becoming a cornerstone of clinical rhythm control therapy for AF (540), several atrial computational modeling studies have tested the effectiveness of different ablation strategies (541, 542). Using CT-based atrial models, Hwang et al. (261) concluded that the most effective ablation would be PVI plus posterior box isolation and anterior line ablation. This hypothesis was evaluated in a prospective clinical trial with 108 persistent AF patients who were randomly selected to receive either standard-of-care ablation or in silico guided ablation (55). However, the proposed in silico ablation consisted of more extensive lesion sets and failed to achieve significant improvement compared with standard-of-care, denoting the need for further work. Using a single patient-specific electromechanical model, Gerach et al. (543)analyzed the impact of five commonly used ablation strategies (PVI, a mitral isthmus line, an anterior line, a roof line, and a posterior box lesion) on the pumping function of the heart, demonstrating that atrial ablation also has acute effects on ventricular performance. Ablating interatrial connections to isolate the atria has also been shown to be successful strategy in a cohort of 12 personalized atrial models (544).

**FIGURE 15.**
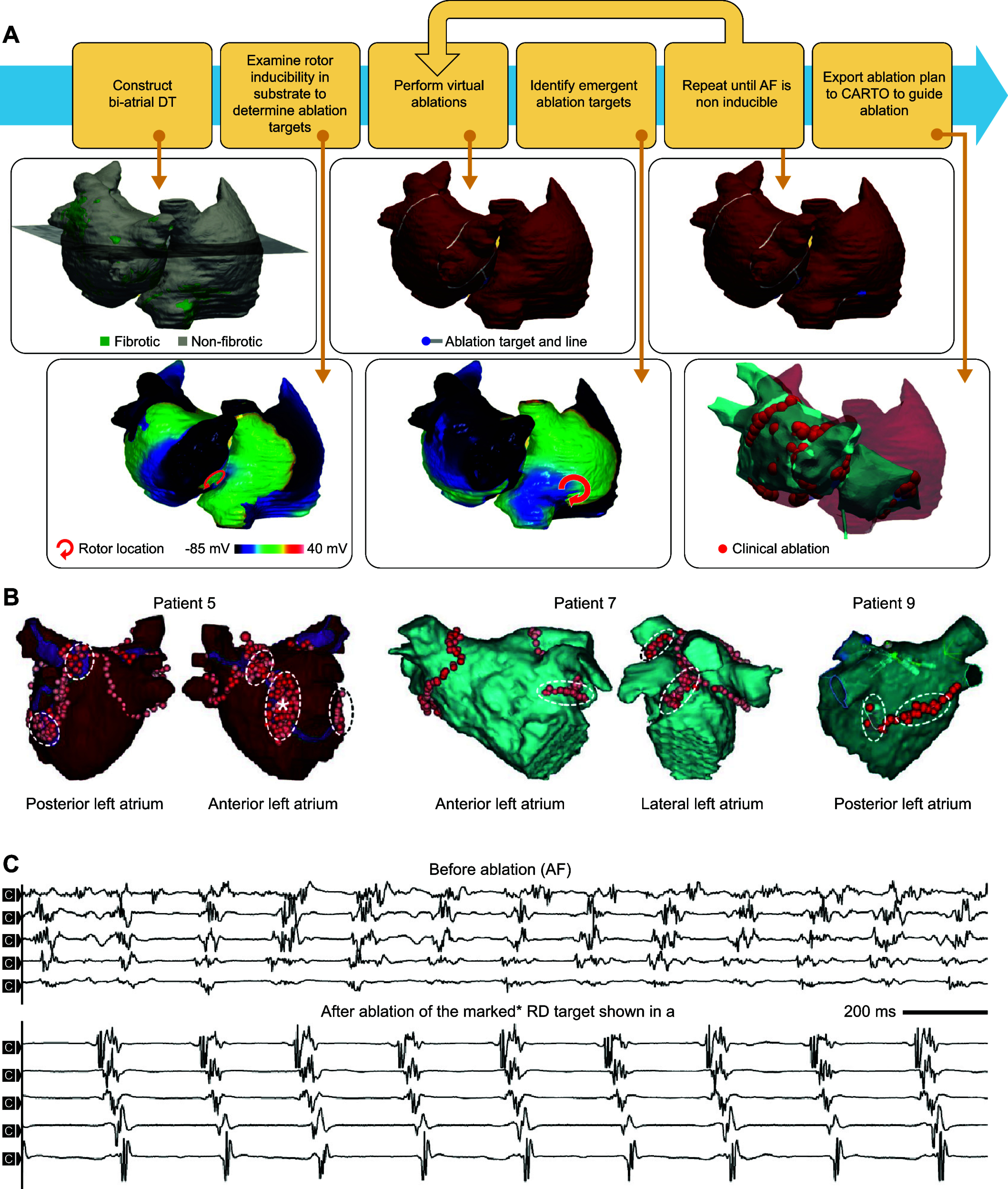
Computational guidance of atrial fibrillation (AF) ablation and assessment of the fibrotic substrate. *A*: the OPTIMA approach. The individual steps are illustrated with the model of 1 of the participants in the study. Late gadolinium enhancement scans were used to construct the patient’s biatrial geometry and fibrosis distribution digital twin (DT). After a baseline inducibility test, 1 location at the left atrial anterior septal wall was determined to have a high likelihood of sustaining a rotor (*bottom left*). After virtual ablations targeting the detected location, a repeat inducibility test identified an emergent rotor location at the right atrial posterior region, which was then ablated. The final optimal ablation targets resulted in a complete arrhythmia noninducibility of the substrate. The proposed targets were imported to the CARTO system to guide ablation procedure. *B*: sites of ablation delivery (with catheter tip locations marked by red dots) in the left atrium, as rendered by the CARTO intracardiac mapping system at the end of the clinical ablation procedure in 3 patients. *Marked RD targets. Modified from Ref. 259, with permission from *Nature Biomedical Engineering*. *C*: bipolar electrogram recordings from a decapolar catheter placed in the coronary sinus of *patient 5*. In *patient 5*, ablation of the marked anterior left atrial target (shown in *B* by an asterisk for *patient 5*) resulted in a transient change from AF (*top* set of 5 electrograms) to an organized atrial tachycardia or flutter (*bottom* set of 5 electrograms). Modified from Ref. 259, with permission from *Nature Biomedical Engineering*.

The discovery of fibrosis as a substrate for AF reentrant drivers has resulted in a large body of research utilizing personalized computationally driven ablation strategies targeting the fibrotic substrate (54, 316, 317, 348, 545, 546). In these studies, ablations targeting AF and atrial flutter drivers restored sinus rhythm in the models. Boyle et al. (259) pioneered a prospective ablation study for patients with persistent AF and fibrosis on LGE-MRI driven entirely by personalized atrial simulations. In this study, termed OPTIMA (OPtimal Target Identification via Modeling of Arrhythmogenesis), 10 patients were enrolled. From the simulations of AF inducibility, the locations of reentrant drivers sustaining AF were determined, ablation performed virtually in the models, and the inducibility process repeated until a set of optimal ablation targets resulting in a complete arrhythmia noninducibility in the substrate was achieved. The proposed ablation targets were then used to steer patient treatment, eliminating not only the clinically manifested AF but also any potential emergent AF drivers (see FIGURE 15*A* for a flowchart showcasing the individual steps in the approach). FIGURE 15, *B* AND *C*, show three patients with CARTO lesions and acute termination of AF. Although this study was very successful, its efficacy is now being tested in a larger cohort. Azzolin et al. (547) used different combinations of imaging (LGE and CT) and EAM data from 29 persistent AF patients to create patient-specific atrial models that were used to determine personalized ablation targets. The results from this study showed that PVI had the lowest acute success rate and that personalized fibrosis-targeted ablation resulted in success rates as high as 90.9%, following repeated inducibility tests and virtual ablations. To determine targets, the study analyzed the dominant frequencies in the EAM, which rendered the prediction requiring invasively obtained data (547). Finally, atrial models have been used, often in combination with machine learning or other technologies, to predict AF recurrence (288, 548, 549).

The use of computationally driven ablation targeting has also been compared with other approaches to determine the locations of AF drivers, such as focal impulse and rotor mapping (FIRM) or ECGI (501, 550, 551). Additionally, personalized atrial models have helped develop better approaches for clinical assessment of the fibrotic substrate and AF propagation patterns (552–556). In an interesting study, Bifulco et al. (557) identified with modeling patients with embolic stroke of undetermined source (ESUS). The study examined the absence of arrhythmia in ESUS despite the presence of putatively proarrhythmic fibrosis. MRI-based atrial models were reconstructed for 45 ESUS and 45 AF patients, and no differences in the fibrotic substrate were found between these patient groups, leading to the conclusion that ESUS patients with fibrotic atria are spared from AF because of an absence of arrhythmia triggers. ESUS and AF models had a statistically similar pattern of inducibility rates, and stimulation from the posterior wall was approximately two times more likely to induce reentrant drivers in AF models than in ESUS models. This suggests that triggered activity in the posterior wall may be more likely to initiate reentrant arrhythmia in AF patients compared with ESUS patients.

## 4. GENERAL OUTLOOK

Employing multiscale computational modeling of the heart, as reviewed in this article, could bring about a fundamental change in the approach to heart rhythm disorder diagnosis, prognosis, and treatment, should it be successfully implemented in the clinical workflow and incorporated in clinical decision making. Computational modeling of the heart (often called digital twinning of the heart) could therefore become a disruptive approach, as it leverages robust physics-based mechanistic insights and is capable of encoding pathophysiological complexity across multiple scales. As demonstrated here, models allow in silico reconstruction of quantities of interest and biomarkers acquired from multiple sources of clinical data, such as MRI, CT, ECGs, and EAMs. Using a computational approach in assessing arrhythmogenesis propensity or electrophysiological disturbances could thus minimize the invasiveness and number of clinical evaluations on patients. Many researchers in the field are developing new algorithmic approaches, methods, and tools for cardiac modeling that will promote a swift uptake in daily clinical practice and clinical translation. Because of the complex interactions among different physical processes and their feedback interactions, novel numerical methods for multiphysics and multiscale couplings are now aiming at containing the computational burden of simulations without compromising the accuracy and stability. In addition, these computational advances are expected to enable more comprehensive models that capture the full complexity of cardiac pathophysiology. For example, many current models simulate only atrial or ventricular electrophysiology. Future work employing full four-chamber models including realistic representation of the conduction system may, for example, help to characterize mechanisms underlying AF-induced tachycardiomyopathy or other conditions in which atrioventricular interactions play a significant role. Likewise, this may enable integration of detailed local control models that can simulate subcellular Ca^2+^ handling abnormalities into organ-level simulations to study the multiscale determinants of ectopic activity (e.g., in catecholaminergic polymorphic VT), or complex Markov models of individual ion channels to accurately simulate organ-level electrophysiological consequences of state-dependent binding of antiarrhythmic drugs. There is also an increased emphasis on model validation with experimental and retrospective clinical studies, as reviewed here, which enables improved personalization of heart models and the gradual development of trust leading to prospective studies utilizing computational modeling in patient treatment and prognostication.

A novel development in the progression of computational modeling of the heart has been the increased synergy with AI approaches in precision cardiology (526, 548, 558–561). The combination of AI models learned from data and mechanistic heart modeling can help furnish mechanistic underpinning and explainability of AI models by incorporating machine learning-uncovered features in computational modeling to understand their role in arrhythmogenesis and electrical dysfunction of the heart. This synergistic inductive and deductive reasoning could thus furnish accurate predictions based on the underlying arrhythmia causes and suggest novel pathways to restore heart rhythm. Physics-based (mech anistic) computational models and the results of simulations also could constitute a solid basis for training AI models, thus allowing the integration of clinical and simulated data to efficiently generate patient-specific responses.Furthermore, it could be expected that the computational expense of mechanistic models will prohibit their application in a predictive capacity in large patient populations, where AI is likely to provide the needed prognostication clinical decision support. However, personalized computational models could provide the interpretation of the AI prediction that applies to the individual patient.

Computational modeling has already transformed traditional areas of physics and engineering. In this review we demonstrate how it could also transform cardiovascular medicine and serve as a vehicle in ushering a plethora of personalized approaches to heart health. In addition to its value in precision cardiology, computational modeling is expected to provide significant value in general biomedical discovery, as data from multiple sources can be combined in a heart model to uncover patterns and features governing the behavior of multiple interacting organ systems and long-term outcomes. We expect that the methodological innovation and predictive capabilities of the computational modeling technology will provide impetus to other such approaches in biomedical discovery, will expand our knowledge of the pathophysiology of human disease, and will inspire new studies and the formulation of novel hypotheses.

## 5. CONCLUSIONS

Multiscale computational models of the heart, if successfully adopted in clinical practice, would result in a paradigm shift in the management of patients with heart rhythm disorders. Such robust noninvasive approaches for individualized arrhythmia risk assessment and guidance of rhythm disorder therapies could save human life, could lead to optimized therapy delivery and reduction in health care costs, and will have a dramatic personal, medical, and economic impact on society.

## GRANTS

This research was supported by National Heart, Lung, and Blood Institute Grants R01HL166759 and R01HL142496 and a grant from the Leducq Foundation to N.A.T., as well as by The Netherlands Organization for Scientific Research NWO/ZonMW Vidi 09150171910029 and the Dutch Heart Foundation (grant no. 01-002-2022-0118, EmbRACE consortium) to J.H.

## DISCLOSURES

No conflicts of interest, financial or otherwise, are declared by the authors.

## AUTHOR CONTRIBUTIONS

N.A.T., A.L., J.S., and J.H. conceived and designed research; prepared figures; drafted manuscript; edited and revised manuscript; and approved final version of manuscript.
